# Kinetics of Electrophilic Fluorination of Steroids and Epimerisation of Fluorosteroids

**DOI:** 10.1002/chem.202001120

**Published:** 2020-08-25

**Authors:** Neshat Rozatian, Antal Harsanyi, Ben J. Murray, Alexander S. Hampton, Emily J. Chin, Alexander S. Cook, David R. W. Hodgson, Graham Sandford

**Affiliations:** ^1^ Chemistry Department Durham University South Road Durham DH1 3LE UK

**Keywords:** epimerisation, fluorination, fluorosteroid, kinetics, stereoselectivity

## Abstract

Fluorinated steroids, which are synthesised by electrophilic fluorination, form a significant proportion of marketed pharmaceuticals. To gain quantitative information on fluorination at the 6‐position of steroids, kinetics studies were conducted on enol ester derivatives of progesterone, testosterone, cholestenone and hydrocortisone with a series of electrophilic N−F reagents. The stereoselectivities of fluorination reactions of progesterone enol acetate and the kinetic effects of additives, including methanol and water, were investigated. The kinetics of epimerisation of 6β‐fluoroprogesterone to the more pharmacologically active 6α‐fluoroprogesterone isomer in HCl/acetic acid solutions are detailed.

## Graphical abstract

**Fluorosteroid pharmaceuticals** are prepared industrially by electrophilic fluorination. Quantitative insights into the synthesis of 6‐fluorosteroids could lead to more efficient processes. Here, kinetic and analytical studies upon a range of fluorinations of steroid enol esters, and subsequent epimerisation towards the α‐epimer are described. X‐ray crystallography is exploited to deliver unambiguous assignments of absolute configurations.

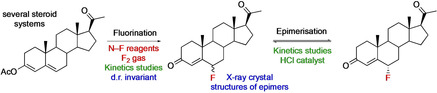

## Introduction

**Figure 1:**
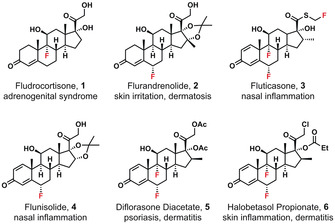
Examples of biologically‐active fluorinated corticosteroid drugs currently on the market.

In the history of the development of fluorine‐containing drug substances, fluorosteroids made significant early contributions when Fried and Sabo discovered that the introduction of a single fluorine atom into a corticosteroid **1** increased its potency tenfold.^1^ Since this observation, numerous fluorinated steroids have been marketed for the treatment of various disease classes, including cancers and inflammation.^2^ In particular, fluorosteroids bearing a fluorine atom at the 6‐position, such as flurandrenolide **2** and fluticasone **3**, continue to be commercially significant (Figure 1). Fluticasone therapeutics were ranked in the top 200 drugs prescribed in the USA in 2017.^3^ The introduction of a fluorine atom at the 6‐position is normally carried out by reaction of a steroid enolate derivative with an electrophilic fluorinating agent. Early examples of this transformation included the use of perchloryl fluoride (ClO_3_F)^4^ and trifluoroacetyl hypofluorite (CF_3_COOF),^5^ but due to the hazardous natures of these reagents, they were not suitable for large scale use. In recent years, several electrophilic fluorinating reagents of the N−F class, such as *N*‐fluoropyridinium salts,^6^ NFSI,^7^ Selectfluor™,^8^ and Accufluor™^9^ have been used for the fluorination of steroid enolate derivatives. Within the N−F class of reagents, Selectfluor™ has been used in larger scale applications, such as in the manufacture of fluticasone **3**.^10^ Indeed, an estimated 80 % of commercially available fluorosteroids are synthesised using Selectfluor™.^11^, ^12^, ^13^, ^14^

The introduction of a fluorine atom using an N−F reagent has also been achieved at other positions within the steroid; for example, Lectka et al. recently described the photocatalytic fluorination of steroids at the 15‐position using Selectfluor™.^15^ 6‐Fluorosteroids are generally formed as mixtures of 6α‐ and 6β‐isomers, where the former is usually pharmacologically active.^16^, ^17^ The ratio depends upon the fluorinating reagent employed, steroid structure, temperature and timescale of the reaction. These factors were explored by Herrinton et al.^18^ using several N−F reagents, where Selectfluor™ was determined to be the most efficient fluorinating agent.

To our knowledge, there have been no kinetics studies on the electrophilic fluorination of steroidal enolate systems, despite their therapeutic and commercial importance. However, recent studies by Nelson et al.^19^ reported the kinetics of fluorination of a related tetralone system using Selectfluor™. These studies established the mechanistic pathway of fluorination using Hammett correlations, concluding that an S_N_2 reaction occurred rather than SET. Furthermore, the kinetics and mechanisms of acid‐mediated epimerisations of fluorosteroid mixtures to their more pharmacologically active α‐isomers remain underexplored.

**Figure 2:**
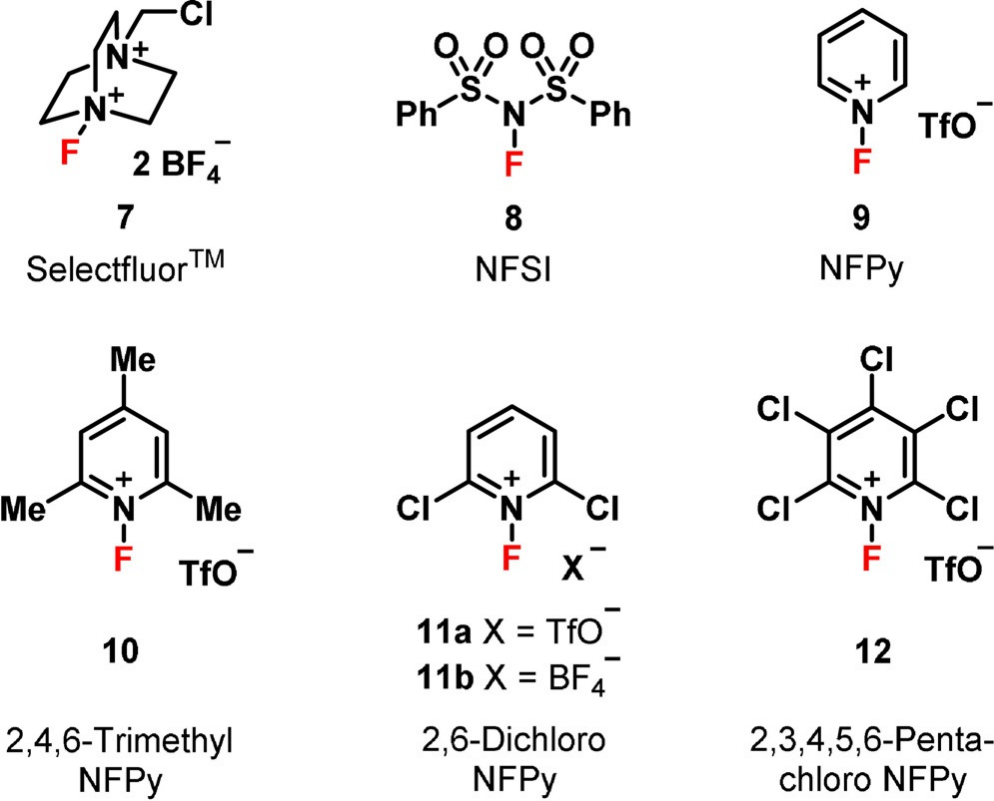
Fluorinating reagents of the N−F class investigated in this study (NFPy=*N*‐fluoropyridinium).

General studies on the mechanisms and reactivities of N−F reagents towards carbon nucleophile systems have also recently been performed. Mayr et al.^20^ used a heterogeneous range of C‐nucleophile systems to provide evidence for an S_N_2 mechanism and quantitative electrophilicity values, based on the Mayr–Patz scale, for five N−F fluorinating reagents. We used a homologous series of enolic *para*‐substituted 1,3‐diaryl‐1,3‐dicarbonyl derivatives to report a quantitative reactivity scale for ten electrophilic N−F fluorinating reagents (Figure 2) and deliver Hammett correlations that support S_N_2 fluorine transfer.^21^ We recently extended our studies to explore the factors affecting difluorination of our 1,3‐dicarbonyl compound series, concluding that solvent effects dramatically enhance enolization rates, thus promoting difluorination.^22^

**Scheme 1:**
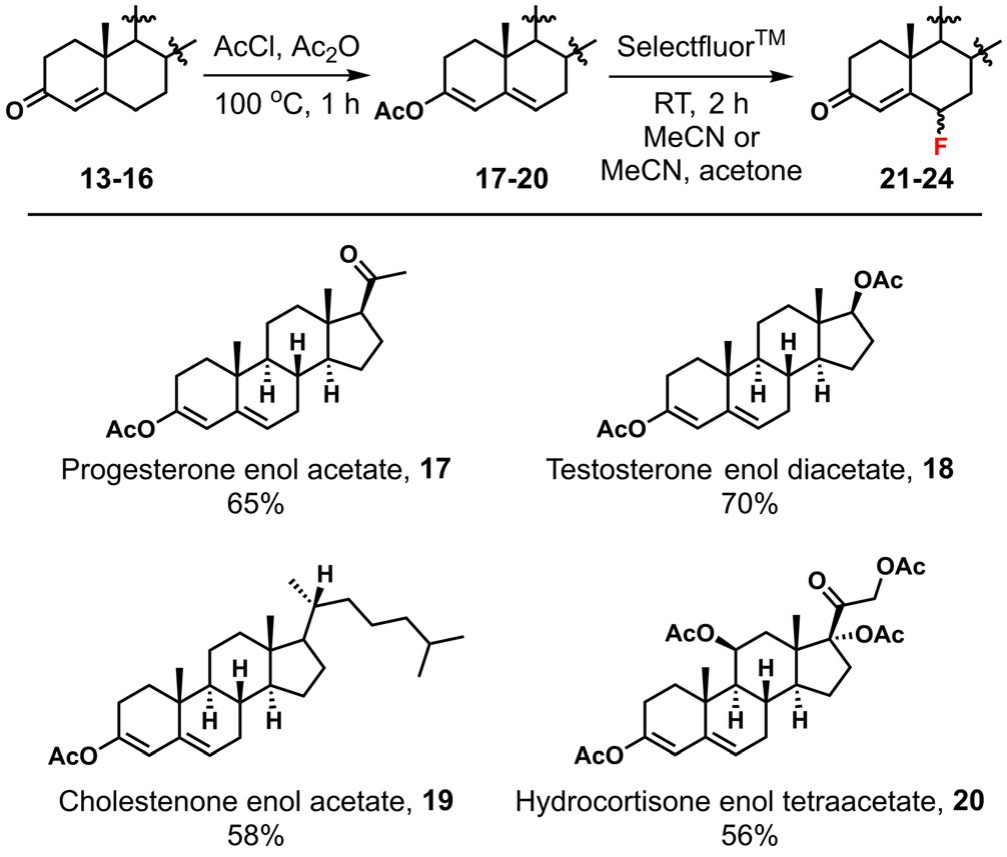
Synthesis of enol acetates **17**–**20** from progesterone **13**, testosterone **14**, cholestenone **15** and hydrocortisone **16**, respectively. Fluorination of enol acetates **17**–**20** using Selectfluor™ to obtain the corresponding 6‐fluorosteroids **21**–**24** as mixtures of β and α isomers.

Quantitative approaches offer the possibility of matching the reactivities of nucleophilic and electrophilic partners, allowing reaction rates to be estimated and rapid, but controlled, processes to be designed.^23^ Given the pharmaceutical importance of 6‐fluorosteroidal systems, we sought to quantify the nucleophilicities of enol equivalents of four main classes of steroids, namely; progesterone **13** (a progestogen), testosterone **14** (an androgen), cholestenone **15** (a cholesterol precursor) and hydrocortisone **16** (a corticosteroid). Each of these steroids contains an enolisable α,β‐unsaturated ketone system that can direct fluorination to their 6‐positions via the corresponding enol ester systems **17**–**20** (Scheme 1), which are readily synthesised in one step.^24^, ^25^

We explore a range of fluorinating reagents, including N−F reagents and fluorine gas, and the factors that affect stereochemical outcome. We also perform kinetic studies upon epimerisation processes that are used to redress the balance between the kinetically favoured β‐isomers that arise from fluorination processes and the pharmaceutically desired α‐isomers.

Most electrophilic fluorinating reagents are synthesised from fluorine gas (F_2_), however, there have been no reports of the corresponding fluorinations of steroidal enol ester systems at the 6‐position using fluorine gas itself, although there are several reports of fluorination of tertiary C−H positions in steroid substrates using F_2_.^26^, ^27^ Since selective direct fluorination of steroids by fluorine gas could provide a more cost‐effective, greener route to commercially important 6‐fluorosteroid derivatives, we used progesterone enol acetate **17** to study direct fluorination using fluorine gas.^28^

## Results and Discussion

### Preparation of materials and stereochemical characterisation

In order to assess kinetics of enol acetate fluorination and subsequent epimerisation of fluorosteroids, we prepared a series of enol acetate substrates and isolated α‐ and β‐fluorosteroid isomers. The facial selectivities of a range of reagents were determined by NMR spectroscopy. The absolute configurations of the fluorosteroid products were, in some cases, confirmed crystallographically.

**Scheme 2:**
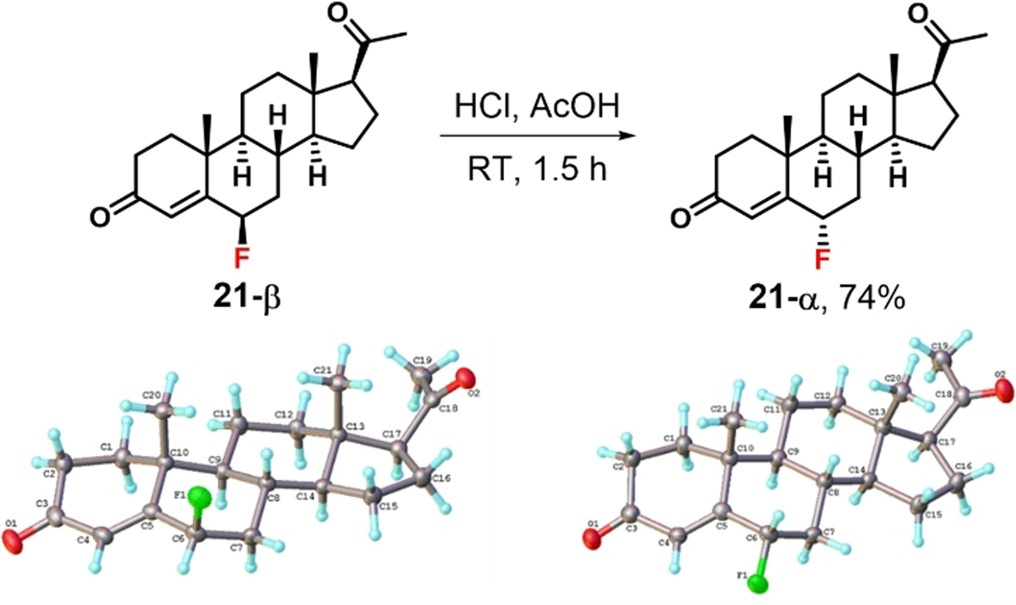
Epimerisation of 6‐fluoroprogesterone **21‐β** to **21‐α** in HCl/AcOH and X‐ray crystallographic structures for each isomer.

Progesterone enol acetate **17** was synthesised in 65 % yield following a modified literature procedure (Scheme 1).^24^ Spectroscopic analyses were in agreement with previous reports^25^ and the structure was further confirmed by X‐ray crystallography (see Supporting Information Section 2.1). Fluorination of **17** was conducted using Selectfluor™ (Scheme 1) to obtain a mixture of α and β isomers of 6‐fluoroprogesterone **21**.^8^ The fluorination proceeded cleanly, with 100 % conversion as determined by ^1^H and ^19^F NMR spectroscopy, and 96 % yield of the α/β isomer mixture, where the α and β isomers were present in a 34:66 ratio. The isomer mixture was resolved using column chromatography to afford isolated yields of 19 % α‐isomer and 46 % β‐isomer. The structures of the isomers were assigned by X‐ray crystallography (Scheme 2).

Testosterone enol diacetate **18**, cholestenone enol acetate **19**, hydrocortisone enol tetraacetate **20** and their fluorinated derivatives **22**–**24** (total yields of 69–78 % for both isomers) were synthesised and isolated using the same procedures as progesterone. X‐ray crystallographic analyses of testosterone derivatives **18** and **22** were also obtained (see Supporting Information Section 2.1).

In the case of fluoroprogesterone **21**, the β‐isomer was converted to the more thermodynamically stable α‐form by epimerisation of the crude product mixture. We used the procedure reported by Ringold,^29^ where the crude mixture of isomers of **21** was dissolved in acetic acid, and dry HCl gas was bubbled through the solution for 1.5 h (Scheme 2). Following evaporation of solvents and recrystallisation from MeOH, **21‐α** was obtained in 74 % yield.

The α:β isomer ratios of each fluorinated steroid crude reaction mixture, prepared using N−F reagents, are summarised in Table 1. When Selectfluor™ was used as the fluorinating reagent, testosterone enol diacetate **18** resulted in the highest levels of the desired α isomer compared with the other steroidal nucleophiles. The crude α:β isomer ratios for fluorinations of progesterone enol acetate **17** by seven N−F reagents were determined, and the reaction with Selectfluor™ gave the highest proportion of the α isomer. The stereoselectivity does not follow the trend in reactivities of the N−F reagents. As we have previously reported,^21^ diCl‐NFPy TfO^−^/BF_4_^−^ **11 a/b** are 4‐fold less reactive than Selectfluor™ and show the lowest selectivities for the α isomer. NFSI **8**, NFPy TfO^−^ **9** and triMe‐NFPy TfO^−^ **10** are 4—6 orders of magnitude less reactive than Selectfluor™, and pentaCl‐NFPy TfO^−^ **12** is 1 order of magnitude more reactive, yet, all lead to similar stereoselectivities.

**Table 1:** Ratios of 6α‐ to 6β‐fluorosteroids **21**–**24** formed upon fluorination of **17**–**20** by N−F reagents, as determined by ^1^H and ^19^F NMR spectroscopies in MeCN‐*d*_3_.

Steroidal nucleophile	N−F reagent	Ratio of 6α:6β fluorosteroids
progesterone enol acetate **17**	Selectfluor™ **7**	34:66
	NFSI **8**	23:77
	NFPy TfO^−^ **9**	22:78
	triMe‐NFPy TfO^−^ **10**	23:77
	diCl‐NFPy TfO^−^ **11 a**	13:87
	diCl‐NFPy BF_4_^−^ **11 b**	13:87
	pentaCl‐NFPy TfO^−^ **12**	20:80
testosterone enol diacetate **18**	Selectfluor™ **7**	43:57
cholestenone enol acetate **19**	Selectfluor™ **7**	38:62
hydrocortisone enol tetraacetate **20**	Selectfluor™ **7**	35:65

These results are surprising considering the vast range of reactivities and differing steric requirements of the fluorinating reagents. The near‐identical α:β isomer ratios suggested that epimerisation at the newly‐formed C−F centre could be in operation, with the isomer ratio being determined by solvent‐product interactions. Closer inspection of ^1^H NMR kinetic data for the fluorinations of progesterone enol acetate **17** with NFPy TfO^−^ **9** and NFSI **8** showed constant α:β isomer ratios over the courses of the reactions. In order to further investigate the potential for in situ epimerisation we explored whether ‘spent’ Selectfluor™ (ClCH_2_‐DABCO^+^ **⋅**BF_4_^−^) could play an acid/base catalysis role in this process. When **21‐β** was incubated with ClCH_2_‐DABCO^+^ **⋅**BF_4_^−^ in MeCN‐*d*_3_, no formation of **21‐α** was observed by ^19^F NMR spectroscopy over the course of 1 week. To probe the possibility of protonated ‘spent’ Selectfluor™ catalysing in situ epimerisation, we attempted to prepare a sample of this protonated species, although our efforts were unsuccessful (see Supporting Information Section 2.1.12). Thus, at present, we are unable to confirm that in situ epimerisation is in operation.

**Scheme 3:**
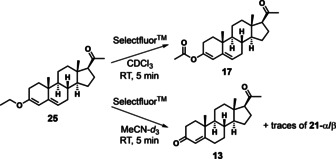
Reaction of 3‐ethoxy‐pregna‐3,5‐dien‐20‐one **25** with Selectfluor™ (0.95 equiv) in CDCl_3_ gave the oxidised product **17**, as identified by HR‐MS, and fluorinated products were not detected. The HR‐MS fragment ions consistent with **17** were: [*M*+H]^+^=357.243, [M+H‐CH_3_CO]^+^=315.228, [M‐CH_3_COO]^+^=297.215. However, in the reaction of **25** with Selectfluor™ (1.0 equiv) in MeCN‐*d*_3_, progesterone **13** was the major product, and traces of fluorinated products were present.

To explore the effects of other enol derivatives upon fluorination kinetics and stereoselectivity, the ethylated form of the progesterone enol, 3‐ethoxy‐pregna‐3,5‐dien‐20‐one **25**, was prepared using a previously described method.^30^ This compound is the starting material for the synthesis of birth control drug quingestrone and several related compounds.^31^ The electrophilic chlorination of **25** with *N*‐chlorosuccinimide (NCS) was conducted by Ringold et al.^32^ although, to the best of our knowledge, the fluorination of **25** has not been reported. On mixing **25** and Selectfluor™ (0.95 equiv) in CDCl_3_, an immediate colour change from yellow to red was observed (Scheme 3). CDCl_3_ was used due to the lower solubility of **25** in MeCN and acetone. Analysis of the reaction mixture by ^19^F NMR spectroscopy showed the disappearance of the N−F signal, however, new signals were not observed. Analysis by high resolution mass spectrometry (HR‐MS) showed the formation of a product consistent with oxidation of **25** by introduction of a single oxygen atom. The fragment ions showed loss of Ac‐ and AcO‐ groups, which indicate that the oxidised product is progesterone enol acetate **17**. Selectfluor™ is a known oxidant, for example, the copper‐mediated oxidation of amides to imides by Selectfluor™ has been reported.^33^ The oxidation of an ethoxy group to an acetyl group was previously reported for the conversion of 6‐ethoxybenzothiazole‐2‐sulfonamide to the corresponding acetate with trichloroisocyanuric acid,^34^ a reagent that is known to carry out both chlorination and oxidation.^35^ Oxidation may be specific at this position due to the non‐bonding electrons on the ethereal oxygen atom that could activate the neighbouring C−H bonds by hyperconjugation effects.^36^ We believe oxidation processes are likely to result in the formation of HF, which will be lost from the reaction mixture, thus leading to the loss of detectable ^19^F NMR signals over the course of the reaction.

When the reaction was conducted in MeCN‐*d*_3_ with 1 equiv of Selectfluor™ (with sonication to fully solubilise **25**), small amounts of **21‐α** and **21‐β** were detected by ^1^H and ^19^F NMR spectroscopy, as well as traces of unidentified fluorinated products (NMR spectra are included in the Supporting Information Section 2.1.11). Progesterone enol acetate **17** was not detected; however, the major product of the reaction was progesterone **13**, which could have formed by oxidation of **25** to **17**, followed by hydrolysis of the ester group to form **13**. The formation of **13** was confirmed by comparison of the spectra with an authentic sample of **13**.

### Kinetics of fluorination of steroid enol acetates

**Figure 3:**
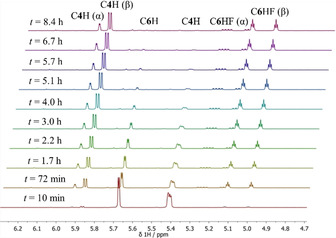
Fluorination of progesterone enol acetate **17** by NFSI in MeCN‐*d*_3_ at 25 °C, monitored by ^1^H NMR spectroscopy. Starting concentrations of reagents: [**17**]=17.5 mm, [NFSI]=526.0 mm. Signals corresponding to **17** are labelled as C6H and C4H. Peaks associated with fluoroprogesterone isomers **21‐α** and **21‐β** are also labelled.

The kinetics of fluorination of progesterone enol acetate **17** by NFSI **8**, NFPy TfO^−^ **9** and triMe‐NFPy TfO^−^ **10** were monitored by quantitative ^1^H NMR spectroscopy in MeCN‐*d*_3_ (Figure 3). These kinetics experiments were carried out with excess N−F reagent to achieve *pseudo*‐first order conditions. Due to the wide range of reactivity of the N−F reagents, reactions involving Selectfluor™ **7**, diCl‐NFPy TfO^−^ **11 a**, diCl‐NFPy BF_4_^−^ **11 b** and pentaCl‐NFPy TfO^−^ **12** were too rapid to be monitored by NMR spectroscopy. Hence, we used UV/Vis spectrophotometry, where the use of lower concentrations of the reaction partners was expected to afford lower observed rates. Although the fluorinations of steroid enol acetates discussed in the previous section were carried out in MeCN‐acetone mixtures to maximise the solubilities of the reaction partners, solubility was not an issue at the lower concentrations used in UV/Vis spectrophotometry and NMR spectroscopy studies. Hence, kinetics studies were conducted in MeCN only. Extinction coefficients were determined for **17**, **21‐α** and **21‐β** (see Supporting Information Section 2.4.4) with the aim of enabling us to determine **21‐α**:**21‐β** ratios in our reaction mixtures spectrophotometrically. Although there was a difference between the extinction coefficients for **21‐α** and **21‐β**, we were unable to reliably extract ratios. Since Selectfluor™ **7** is not chromophoric, we were able to monitor the decays in absorbance of progesterone enol acetate **17** at 236 nm with an excess of Selectfluor™ **7**. However, the UV/Vis spectra of **11 a**, **11 b** and **12** contain absorbance bands between 200–350 nm and it was not possible to selectively monitor the steroid band at 236 nm. Therefore, the kinetics experiments involving reagents **11 a**, **11 b** and **12** were conducted with excess **17** by monitoring the N−F reagent absorbance at *λ*_max_=288 nm (for **11 a** and **11 b**) and *λ*_max_=320 nm (for **12**).

**Figure 4:**
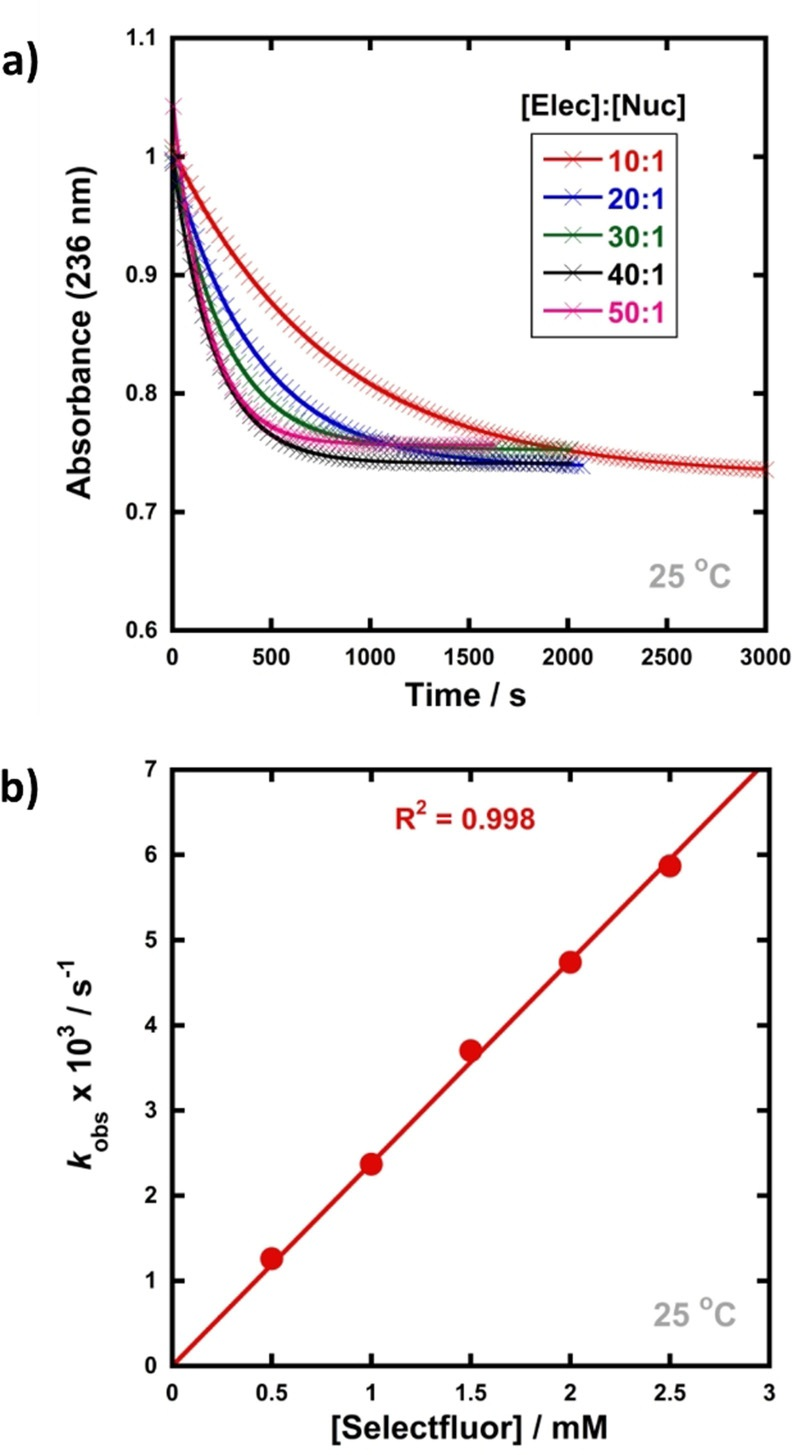
(a) Exponential decays of absorbance of progesterone enol acetate **17** (50 μm) with different concentrations of Selectfluor™ (0.5–2.5 mm) in MeCN at 25 °C. (b) Correlation of *k*_obs_ with [Selectfluor™].

A representative example is shown in Figure 4 for the fluorination of progesterone enol acetate **17** by Selectfluor™ **7**. Clean exponential decays of absorbance of the nucleophile were observed in all runs, and the first‐order rate constants *k*_obs_ were obtained from the fitting of plots of absorbance versus time (Figure 4 a). The *k*_obs_ values were plotted against N−F reagent concentration and a linear (i.e., first order) correlation was observed (Figure 4 b). The direct dependence upon N−F reagent concentration demonstrates rate‐limiting fluorination and thus the slope of this graph gave the second‐order rate constant, *k*_2_ [M^−1^ s^−1^], according to the second‐order rate Equation (1):

(1) $$\mathrm{Rate}=-\frac{d\left[Nuc\right]}{dt}\;=k{}_{2}\left[\mathrm{Nuc}\right]\left[\mathrm{NF}\;\mathrm{reagent}\right]$$

Kinetics studies were conducted on the fluorination of steroid enol acetates **18**, **19** and **20** using similar procedures and the *k*_2_ values are reported in Table 2, as well as those of **17**. All spectra relating to kinetics studies on fluorination of **17**–**20** are included in the Supporting Information Section 2.4. Relative rate constants (*k*_rel_) were determined using Equation (2), with Selectfluor™ as the reference electrophile, thus enabling a comparison of reactivities (Table 2). The reactivity trends of the N−F fluorinating agents match those we previously reported for fluorinations of enolic 1,3‐dicarbonyl systems **26**,^21^ which reinforces the predictive nature of our reactivity scale. More detailed comparisons of reactivities between the two nucleophile systems are included in the Supporting Information Section 2.5.

(2) $$k_{rel}=\frac{k_{2}\left(NF\;reagent\right)}{k_{2}\left({Selectfluor}^{TM}\right)}$$

**Table 2:** Rate constants (*k*_2_) for the fluorination of steroid enol acetates **17–20** by N−F reagents **7–12** in MeCN or MeCN‐*d*3 at 25 °C. The *k*_rel_ values were determined using Equation 2.

Nucleophile	Electrophile	*k*_2_ [m ^−1^ s^−1^]	*k*_rel_
progesterone enol acetate **17**	Selectfluor™ **7**	2.38	1.0
	NFSI **8**	3.33×10^−4^	1.4×10^−4^
	NFPy TfO^−^ **9**	2.08×10^−5^	8.7×10^−6^
	triMe‐NFPy TfO^−^ **10**	7.19×10^−6^	3.0×10^−6^
	diCl‐NFPy TfO^−^ **11 a**	4.72×10^−1^	2.0×10^−1^
	diCl‐NFPy BF_4_^−^ **11 b**	5.03×10^−1^	2.1×10^−1^
	pentaCl‐NFPy TfO^−^ **12**	1.31×10^2^	5.5×10^1^
testosterone enol diacetate **18**	Selectfluor™ **7**	2.11	1.0
	diCl‐NFPy TfO^−^ **11 a**	4.41×10^−1^	2.1×10^−1^
	pentaCl‐NFPy TfO^−^ **12**	1.42×10^2^	6.7×10^1^
cholestenone enol acetate **19**	Selectfluor™ **7**	3.18	1.0
	pentaCl‐NFPy TfO^−^ **12**	1.94×10^2^	6.1×10^1^
hydrocortisone enol tetraacetate **20**	Selectfluor™ **7**	1.06	1.0
	pentaCl‐NFPy TfO^−^ **12**	5.54×10^1^	5.2×10^1^

**Scheme 4:**
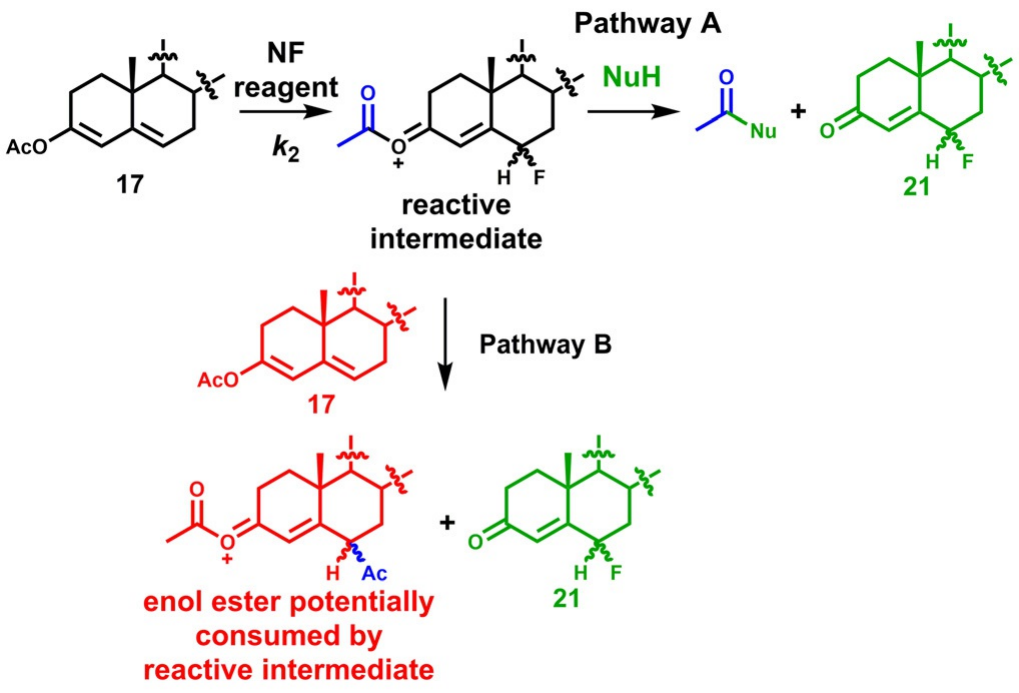
Potential trapping of the reactive intermediate using water or MeOH (Nu=OH, OMe).

**Figure 5:**
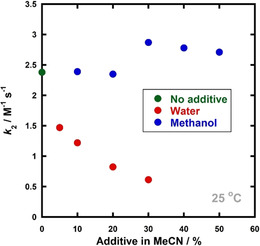
Effects of additives, methanol and water, upon the rate of fluorination of progesterone enol acetate **17**.

Selectfluor™ **7** shows excellent solubility and good stability in water;^22^ additionally, the use of benign solvents such as water is attractive due to the potential for reducing the environmental impact of the process. Furthermore, we recently showed that the addition of water significantly increased fluorination rate constants of the enol forms of 1,3‐dicarbonyl species.^22^ We also wondered whether the trapping of the cationic intermediate generated upon fluorination of **17**, with a weak nucleophilic species, could improve product yield by sequestering this reactive intermediate (Scheme 4, Pathway B). Thus, taking these factors together, we performed kinetics studies using water and MeOH as co‐solvents for fluorination of **17**. Reactions were conducted in the presence of different quantities of water and MeOH, and rate constants were determined using UV/Vis spectrophotometry. All rate constants (*k*_2_) are summarised in the Supporting Information Section 2.4.5. There was little variation in second‐order rate constants, *k*_2_, upon addition of 10–50 % MeOH (*v*/*v* in MeCN). For example, with 30 % MeOH, there was only a 1.2‐fold rate enhancement compared to without MeOH. With water, on the other hand, fluorination rate constants, *k*_2_, decreased as the amount of water was increased (Figure 5). For example, with 30 % water in MeCN (*v*/*v*), the rate of fluorination decreased 4‐fold compared with the analogous reaction in MeCN. Thus, water is not a suitable co‐solvent for increasing the rate of fluorination of **17**, and we tentatively attribute its inhibitory effects to the differing solvation requirements of fluoroenol species in our earlier study^22^ and enol ester **17** along their fluorination reaction coordinates. Product analyses of crude reaction mixtures by LC‐MS were also unchanged by the addition of MeOH or water, and thus we conclude that the use of these co‐solvents offers no advantage to the fluorination process.

Activation parameters (Δ*G* ^≠^, Δ*H* ^≠^, Δ*S* ^≠^) were obtained from kinetic data for the reactions of progesterone enol acetate **17** with two N−F reagents (see Table 3 and Supporting Information Sections 2.4.5 and 2.4.6). With Selectfluor™ **7**, these values were Δ*H* ^≠^ = +51 kJ mol^−1^, Δ*S* ^≠^ = −66 J K^−1^ mol^−1^ and Δ*G* ^≠^ = +71 kJ mol^−1^. With diCl‐NFPy TfO^−^ **11 a**, the values were Δ*H* ^≠^ = +52 kJ mol^−1^, Δ*S* ^≠^ = −76 J K^−1^ mol^−1^ and Δ*G* ^≠^ = +75 kJ mol^−1^. We previously obtained activation parameters for the fluorination of *para*‐substituted enolic 1,3‐dicarbonyl derivatives **26** (vide infra) by Selectfluor™, which were Δ*H* ^≠^=+55 to +64 kJ mol^−1^, Δ*S* ^≠^=−54 to −72 J K^−1^ mol^−1^ and Δ*G* ^≠^=+74 to +83 kJ mol^−1^.^21^ Activation parameters determined by Nelson et al.^19^ for the fluorination of four tetralone derivatives using Selectfluor™ were Δ*H* ^≠^=+62 to +65 kJ mol^−1^, Δ*S* ^≠^=−84 to −100 J K^−1^ mol^−1^ and Δ*G* ^≠^=+90 to +93 kJ mol^−1^. The similarities in these parameters with those of enol ester **17** are consistent with a common S_N_2 mechanism for fluorination of these substrates.

**Table 3:** Activation parameters for the fluorinations of progesterone enol acetate **17** with Selectfluor™ **7** and diCl‐NFPy TfO^−^ **11 a**.

N−F reagent	Δ*G* ^≠^ [kJ mol^−1^]	Δ*H* ^≠^ [kJ mol^−1^]	Δ*S* ^≠^ [J K^−1^ mol^−1^]
Selectfluor™ **7**	+71	+51	−66
diCl‐NFPy TfO^−^ **11 a**	+75	+52	−76

We also attempted to employ fluorine gas to fluorinate progesterone enol acetate **17**. Exploratory experiments were carried out in formic acid solution, which is a preferred solvent for the direct fluorination of enolic systems.^37^ The crude product mixture contained progesterone **13**, 6α‐fluoroprogesterone (**21‐α**) and 6β‐fluoroprogesterone (**21‐β**) along with some minor impurities. However, upon analysis by HPLC, integration of the chromatogram revealed that only half of the crude product mass could be accounted for by these three compounds. Direct fluorination in MeCN yielded mixtures of **21‐α**, **21‐β** and unreacted progesterone enol acetate **17** as well as other fluorinated side‐products, but progesterone **13** was not detected. The selectivity of the direct fluorinations were α:β, 38:62, which is a similar ratio to that obtained using Selectfluor™ **7**. Ultimately, we found batch‐based direct fluorinations to be ineffective, however, flow‐based systems^38^, ^39^, ^40^ may offer improved performance. Further details of direct fluorination experiments are contained in Supporting Information Section 2.3.

### Comparison of nucleophilicities

The relative nucleophilicities, $k_{rel}^{'}$ , of enol acetates **17**–**20**, expressed, defined by Equation (3) (see Table 4) were determined using the second‐order rate constants, *k*_2_ (from Table 2). Unsurprisingly, the reactivity differences are small across the four compounds. Progesterone enol acetate **17** and testosterone enol diacetate **18** have, on average, very similar reactivities. Cholestenone enol acetate **19** is, on average, 1.4‐fold more reactive than **17**, and hydrocortisone enol tetraacetate **20** is 2.3‐fold less reactive than **17**. The major structural change across this range of steroids is the substituent at the remote C‐17 position which, unsurprisingly, appears to have limited effects on their respective nucleophilicities. The electron‐withdrawing acetate groups in **20** result in reduced nucleophilicity of this compound, whereas the inductive electron‐donating alkyl chain at the C‐17 position of **19** increases its nucleophilicity.

(3) $$k_{rel}^{'}=\frac{k_{2}\left(Steroid\;enol\;acetate\right)}{k_{2}\left(Progesterone\;enol\;acetate\;17\right)}$$

**Table 4:** Comparison of reactivities of steroid enol acetates **17–20** using $k_{rel}^{'}$ values defined by Equation 3, determined using the *k*_2_ values summarised in Table 2.

Nucleophile	$k_{rel}^{'}$		
	Selectfluor™ **7**	diCl‐NFPy TfO^−^ **11 a**	pentaCl‐NFPy TfO^−^ **12**
progesterone enol acetate **17**	1.0	1.0	1.0
testosterone enol diacetate **18**	0.89	0.93	1.08
cholestenone enol acetate **19**	1.34	–	1.48
hydrocortisone enol tetraacetate **20**	0.45	–	0.42

**Figure 6:**
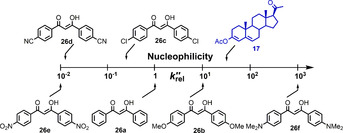
Reactivity scale for nucleophilic enols **26 a**–**f** and enol ester **17**, with enol **26 a** as the reference nucleophile.

The nucleophilic reactivity of progesterone enol acetate **17** was compared with those of the 1,3‐dicarbonyl derivatives **26 a**–**f** using the second‐order rate constants, *k*_2_, for fluorination of these substrates by Selectfluor™ and NFSI (from ref. 21). These two N−F reagents were selected for this comparison since they show markedly different reactivities, as well as having the most extensive datasets for fluorination kinetics. Equation (4) defines $k_{rel}^{{}^{'}{}^{'}}$ . The reactivities of the nucleophiles span 5 orders of magnitude (Figure 6) and enol ester **17** is one order of magnitude more reactive than enol **26 a**. More detailed comparisons of reactivities are included in the Supporting Information Section 2.5.

(4) $$k_{rel}^{{}^{'}{}^{'}}=\frac{k_{2}\left(Nucleophile\right)}{k_{2}\left(26a\;enol\right)}$$

### Kinetics of epimerisation

**Figure 7:**
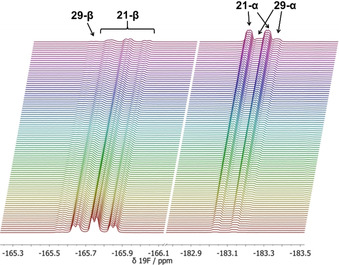
Quantitative time‐arrayed ^19^F NMR spectroscopic monitoring of the epimerisation of **21‐β** (60 mm) to **21‐α** with HCl (0.50 m in AcOH) at 25 °C. Spectra were acquired every 15 min for 17 h with relaxation delays (T1) of 10 s.

The α‐isomers of 6‐fluorosteroids are generally desired because they display higher levels of biological activity.^16^, ^17^ However, the low stereoselectivities of the fluorination reactions discussed earlier result in the formation of the 6β‐isomer as the major product (Table 1). We carried out studies on the rates of epimerisation of 6β‐fluoroprogesterone (**21‐β**) to 6α‐fluoroprogesterone (**21‐α**) by HCl in AcOH, using appropriately diluted solutions of a commercially available reagent. Reactions were monitored directly by quantitative time‐arrayed “in‐magnet” ^19^F NMR spectroscopy with four different concentrations of HCl in acetic acid (0.25–1.00 m). A representative example is shown in Figure 7 for an epimerisation reaction conducted with 0.50 m HCl in acetic acid. The triplet of doublets at *δ*=−165.60 to −165.90 ppm, corresponding to **21‐β**, decreased in intensity over time, whereas the doublet of doublet of doublets at *δ*=−183.00 to −183.16 ppm associated with **21‐α** increased in intensity. Additional peaks were present at *δ*=−165.56 ppm, which overlapped with part of the adjacent **21‐β** signals. Similarly, small peaks appeared over time adjacent to the **21‐α** signals.

**Figure 8:**
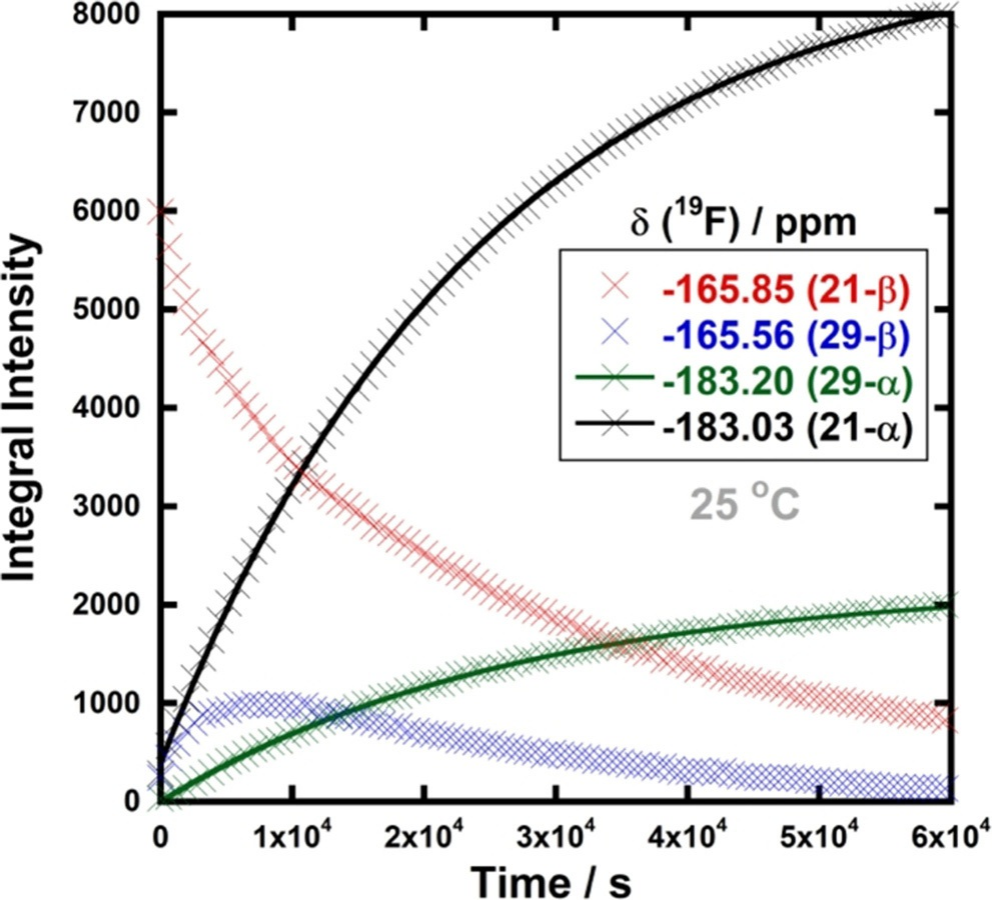
Reaction profiles for the species present in the epimerisation of **21‐β** (60 mm) to **21‐α** with 0.50 m HCl in AcOH. Red: **21‐β**, blue: β‐isomer intermediate **29‐β**, green: α‐isomer intermediate **29‐α**, black: **21‐α**. Due to significant overlap of peaks present, partial signal integration was used for each species, therefore, the integral intensity of **21‐α** (black) at the end of the reaction is higher than that of **21‐β** (red) at the start.

**Figure 9:**
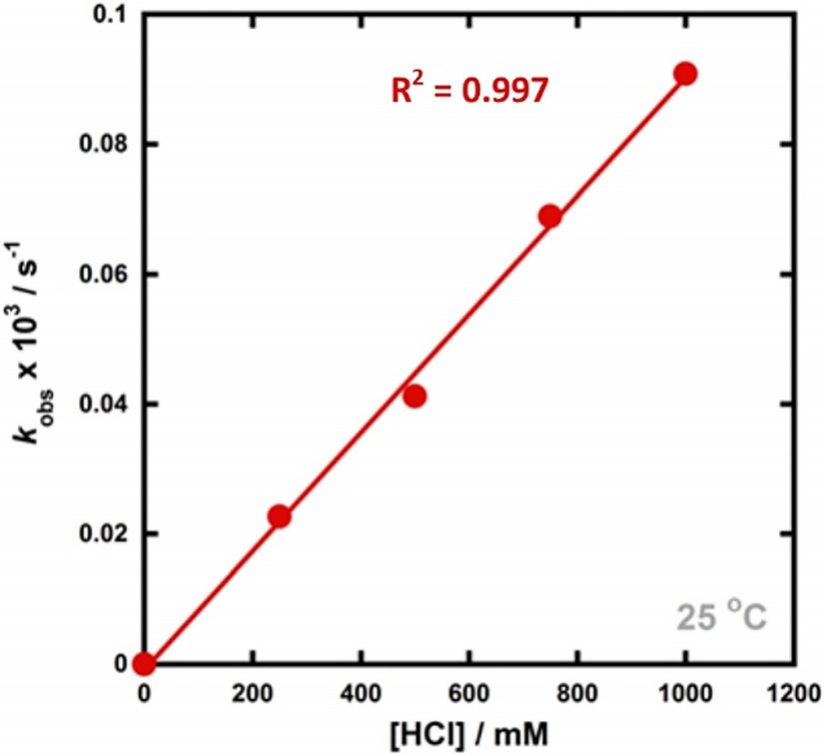
Correlation of *k*_obs_ values versus [HCl] for the epimerisation of **21‐β** to **21‐α** by HCl in acetic acid at 25 °C.

**Scheme 5:**
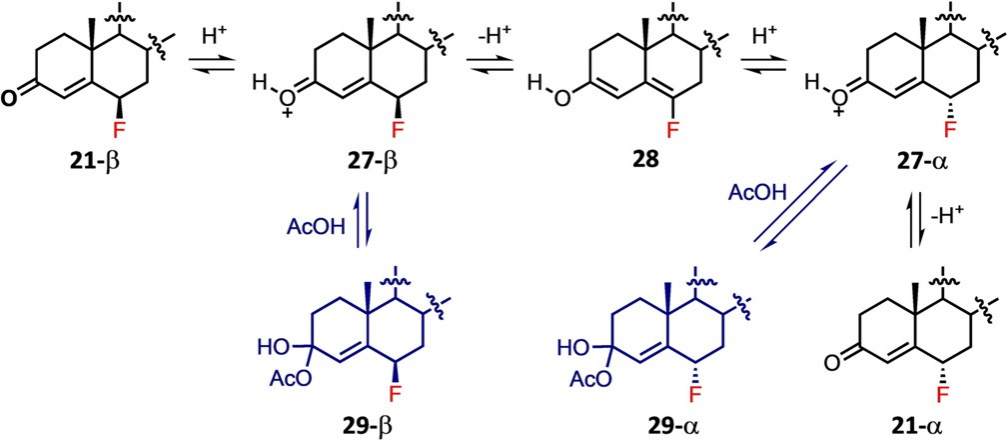
A potential mechanism for epimerisation of **21‐β** to **21‐α**. Another pathway, hemiacylal formation via AcOH (in blue), is proposed.

The reaction profiles for each species in the epimerisation mixture are shown in Figure 8. Due to the overlap between the peaks, partial signal integration was employed. The integrals corresponding to the growth of **21‐α** (black data points) were fitted to an exponential rise function for all concentrations of HCl. A plot of *k*_obs_ versus HCl concentration showed a linear relationship (Figure 9). The integrals of the small signals at *δ*=−165.56 ppm (blue data points) clearly showed the formation and decay of an intermediate (Scheme 5).

The small signals at *δ*=−165.56 and −183.20 ppm in Figure 7 are likely to be product‐related species, due to their similarity in chemical shift and coupling patterns. When an authentic sample of **21‐α** was incubated in a solution of 0.50 m HCl in AcOH for 45 min, the proton‐coupled ^19^F NMR spectrum of the solution showed the presence of signals corresponding to **21‐α** (at *δ*=−183.03 to −183.15 ppm) as well as smaller adjacent signals due to the intermediate species (at *δ*=−183.10 to −183.20 ppm). The proton‐decoupled ^19^F NMR spectrum confirmed that two different species were present (see Supporting Information Section 2.6). However, when **21‐α** was incubated in AcOH alone, signals corresponding to only one species, **21‐α**, were observed. These results suggest acid‐catalysed formation of intermediates such as hemiacylals **29‐β** and **29‐α** (Scheme 5, blue pathways) or enol esters, however, we have no direct evidence of their structures. The change in epimer preference under the reaction conditions from **21‐β** to **21‐α** probably derives from differing solvent‐product interactions. These interactions are likely to be markedly different in AcOH in comparison to the MeCN solvent that was applied for fluorinations.

## Conclusions

The kinetics of fluorination of progesterone enol acetate **17** using seven N−F reagents were studied. The method of analysis was tuned to the reactivity of the system: less powerful electrophiles were studied by ^1^H NMR spectroscopy while more reactive reagents were studied using UV/Vis spectrophotometry. Relative rate constants were determined from absolute rate constants, and they correlate well with our recently reported reactivity scale.^21^ These results highlight the successful predictive power of our scale towards a different class of carbon nucleophiles. Activation parameters were determined for the fluorination of progesterone enol acetate **17** by Selectfluor™ **7** and diCl‐NFPy TfO^−^ **11 a**. The moderately negative values of Δ*S* ^≠^ are consistent with those from our previous studies on fluorination of enolic 1,3‐dicarbonyl systems^21^, ^22^ and recent studies by Nelson et al.^19^ on closely‐related tetralone systems. Kinetics studies on the fluorination of testosterone enol diacetate **18**, cholestenone enol acetate **19** and hydrocortisone enol tetraacetate **20** were conducted. The substituent at the C‐17 position has a small but measurable effect upon the rate of fluorination.

The epimerisation of 6β‐fluoroprogesterone **21‐β** to 6α‐fluoroprogesterone **21‐α** with increasing concentrations of HCl in acetic acid proved to be more rapid. Additional signals in the ^19^F NMR spectra of the product mixtures also gave evidence for the formation of intermediates in an acid catalyst‐dependent manner.

Overall, we have delivered a clearer understanding of the kinetics of fluorination of steroidal systems and the subsequent epimerisation of fluorosteroids. These results highlight the opportunities for achieving more efficient synthetic routes through kinetic understanding.

## Experimental Section

All experimental details can be found in the Supporting Information, which contains characterisations of compounds, details of kinetics experiments and kinetics data.

Deposition Numbers 1985095, 1985096, 1985097, 1985098 and 1985099 contain the supplementary crystallographic data for this paper. These data are provided free of charge by the joint Cambridge Crystallographic Data Centre and Fachinformationszentrum Karlsruhe Access Structures service www.ccdc.cam.ac.uk/structures.

## Conflict of interest

The authors declare no conflict of interest.

## Supporting information

As a service to our authors and readers, this journal provides supporting information supplied by the authors. Such materials are peer reviewed and may be re‐organized for online delivery, but are not copy‐edited or typeset. Technical support issues arising from supporting information (other than missing files) should be addressed to the authors.

Supplementary
