# Supplementary material for: Kinetics of Electrophilic Fluorination of Steroids and Epimerisation of Fluorosteroids
Source: Chemistry. 2020 Aug 25;26(52):12027–35. doi: 10.1002/chem.202001120 (PMC7540021; doi:10.1002/chem.202001120)
Supplement: Supplementary file 1 — Supplementary [file CHEM-26-12027-s001.pdf]

Chemistry–A European Journal

Supporting Information

Kinetics of Electrophilic Fluorination of Steroids and Epimerisation of Fluorosteroids

Neshat Rozatian, Antal Harsanyi, Ben J. Murray, Alexander S. Hampton, Emily J. Chin, Alexander S. Cook, David R. W. Hodgson,* and Graham Sandford*[a]

Table of Contents

1. General Instrumentation and Materials 3

2. Experimental 4

2.1 Synthesis of steroids 4

2.1.1 Progesterone enol acetate 4

2.1.2 6β-Fluoroprogesterone 5

2.1.3 6α-Fluoroprogesterone 6

2.1.4 3-Ethoxy-pregna-3,5-dien-20-one 7

2.1.5 Testosterone enol diacetate 8

2.1.6 6-Fluorotestosterone acetate. 9

2.1.7 (+)-4-Cholesten-3-one enol acetate 10

2.1.8 (+)-6-Fluoro-4-cholesten-3-one. 11

2.1.9 Hydrocortisone enol tetraacetate 11

2.1.10 6-Fluorohydrocortisone triacetate 12

2.1.11 Reactions of 3-ethoxy-pregna-3,5-dien-20-one 25 with N−F reagents 13

2.1.12 Attempted preparation of protonated $ClCH_2$-DABCO$^+$ BF_4^- ('spent' Selectfluor™) 15

2.2 X-ray crystallography 17

2.3 Direct fluorination using fluorine gas 20

2.3.1 Direct fluorination of progesterone enol acetate in formic acid 20

2.3.1.1 HPLC-UV analysis: calibration 20

2.3.1.2 HPLC-UV analysis: crude product from direct fluorination: 23

2.3.2 Direct fluorination of progesterone enol acetate in acetonitrile 24

2.3.2.1 ReactIR study 26

2.4 Kinetics studies on fluorination using N−F reagents 28

2.4.1 Method: NMR spectroscopy 28

2.4.2 Method: UV-vis spectrophotometry 28

2.4.3 Reference UV-vis spectra 29

2.4.4 Extinction coefficients determination 31

2.4.5 Kinetics of fluorination of progesterone enol acetate 17 by Selectfluor™ 7 33

2.4.5.1 Kinetics studies at different temperatures and Eyring correlation 33

2.4.5.2 Kinetics studies with water 38

2.4.5.3 Kinetics studies with methanol 42

2.4.5.4 Solvent effects 47

2.4.6 Kinetics of fluorination of progesterone enol acetate 17 by diCl-NFPy TfO^- 11a at different temperatures and Eyring correlation 48

2.4.7 Kinetics of fluorination of progesterone enol acetate 17 by diCl NFPy BF_4^- 11b 53

2.4.8 Kinetics of fluorination of progesterone enol acetate 17 by pentaCl NFPy TfO^- 12 54

2.4.9 Kinetics of fluorination of progesterone enol acetate 17 by NFSI 8 55

2.4.10 Kinetics of fluorination of progesterone enol acetate 17 by NFPy TfO^- 9 57

2.4.11 Kinetics of fluorination of progesterone enol acetate 17 by triMe NFPy TfO^- 10........ 59

2.4.12 Kinetics of fluorination of testosterone enol diacetate 18 by Selectfluor™ 7........ 60

2.4.13 Kinetics of fluorination of testosterone enol diacetate 18 by diCl-NFPy TfO^- 11a 61

2.4.14 Kinetics of fluorination of testosterone enol diacetate 18 using pentaCl-NFPy TfO^- 12 62

2.4.15 Kinetics of fluorination of cholestenone enol acetate 19 by Selectfluor™ 7 63

2.4.16 Kinetics of fluorination of cholestenone enol acetate 19 by pentaCl-NFPy TfO^- 12........ 64

2.4.17 Kinetics of fluorination of hydrocortisone enol tetraacetate 20 by Selectfluor™ 7..... 65

2.4.18 Kinetics of fluorination of hydrocortisone enol tetraacetate 20 by pentaCl-NFPy TfO^- 12 66

2.5 Comparison of electrophilicities and nucleophilicities 67

2.6 Kinetics studies on epimerisation of β-fluoroprogesterone to α-fluoroprogesterone 69

3. References 73

1. General Instrumentation and Materials

^{1}H, ^{19}F and ^{13}C NMR spectra were obtained using a Bruker Avance-400 spectrometer (^{1}H NMR at 400 MHz, ^{19}F NMR at 376 MHz and ^{13}C NMR at 101 MHz) or Varian VNMRS-600 spectrometer (^{1}H NMR at 600 MHz and ^{13}C NMR at 151 MHz) using residual solvent peaks as the internal standard (^{1}H NMR: $CHCl_3$ at 7.26 ppm; ^{19}F NMR: $CFCl_3$ at 0.00 ppm; ^{13}C NMR: $CDCl_3$ at 77.16 ppm). Accurate mass analysis was performed on a Xevo QtoF mass spectrometer (Waters Ltd, UK) with an accurate solids analysis probe (ASAP). Melting point data were obtained using a Gallenkamp apparatus at atmospheric pressure and are uncorrected. Infra-red (IR) spectroscopy was performed on a Perkin Elmer 1600 Series FTIR with an ATR probe.

Kinetic NMR data discussed in Section 2.4, using the discontinuous method, were acquired on a Bruker Avance-400 spectrometer. “In-magnet” kinetic NMR data discussed in Section 2.6 were acquired on a Varian Inova-500 spectrometer. *In-situ* IR spectroscopy was performed using a Mettler Toledo React IR instrument equipped with a diamond probe.

Fluorinations with F_2 gas were carried out in a glass fluorination reactor (100 mL, 250 mL or 500 mL). The reactor was built from a standard glass bottle with GL 45 thread joint and a PTFE screw cap or a glass flange head, equipped with a gas inlet/outlet head built of Stainless Steel, PTFE and FEP Swagelok components. HPLC analysis of steroid samples was carried out on a PerkinElmer instrument with UV detection at 237 nm using an XBridge C18, 100 × 4.6 mm, 3.5 µm (Waters) column at 25 °C with the following method: elution started with 60% water/40% acetonitrile with 0.1% TFA which was increased to 5% water/95% acetonitrile with 0.1% TFA over 15 minutes at 1.5 mL min^{-1} flow rate.

NMR solvents were purchased from Cambridge Isotopes Inc., supplied by Goss Scientific and Sigma-Aldrich. Organic solvents were used without further purification. HPLC grade MeCN (Romil SpR Super Purity Reagent), HPLC grade MeOH (Fisher Scientific) and HCl solution 1.0 M in AcOH (Sigma Aldrich) were used. Steroids **13**-**16** were purchased from Sigma Aldrich. Selectfluor™ and NFSI were purchased from Fluorochem. Dichloro-NFPy TfO^- was purchased from Sigma Aldrich. Dichloro-NFPy BF_4^-, NFPy TfO^- and trimethyl-NFPy TfO^- were purchased from TCI. Pentachloro-NFPy TfO^- and $ClCH_2$-$DABCO^+$ BF_4^- were prepared using previously reported procedures.[1,2]

2. Experimental

2.1 Synthesis of steroids

2.1.1 Progesterone enol acetate

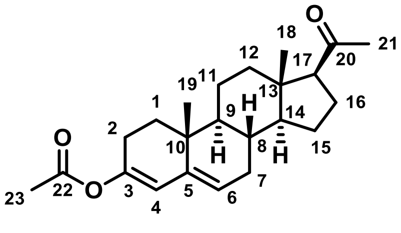

17

Progesterone **13** (14.65 g, 46.5 mmol) was dissolved in a mixture of acetyl chloride (30 mL, 420 mmol) and acetic anhydride (40 mL, 250 mmol) and was heated to 100 °C for 1 h. Upon cooling to room temperature, the mixture was concentrated to one third of the original volume under reduced pressure at 25 °C whereupon a white precipitate formed. The product was filtered and washed with cold acetonitrile (2 × 10 mL) and dried under vacuum to afford progesterone enol acetate (10.74 g, 65%) as a white solid. M.p. 119-123 °C (lit.[3] 130-132 °C, cryst from methanol). IR (cm^{-1}): 2939, 1748, 1703, 1365, 1219, 1202, 1188, 1119. ^{1}H NMR ($CDCl_3$, 600 MHz): 0.65 (3H, s, C**18**H_3), 1.00 (3H, s, C**19**H_3), 1.04-1.10 (1H, m, C**9**-H), 1.18-1.29 (2H, m, C**14**H, C**15**H), 1.30-1.38 (1H, m, C**1**H), 1.43-1.50 (2H, m, C**11**H, C**12**H), 1.58-1.73 (5H, m, C**11**H, C**16**H, C**15**H, C**7**H, C**8**H), 1.85 (1H, dd, $^{2}J_{HH}$ 12.5, $^{3}J_{HH}$ 5.5; C**1**H), 2.03-2.06 (1H, m, C**12**H), 2.12 (3H, s, C**23**H_3), 2.13 (3H, s, C**21**H_3), 2.14-2.22 (3H, m, C**16**H, C**2**H, C**7**H), 2.41-2.47 (1H, m, C**2**H), 2.54 (1H, t, $^{3}J_{HH}$ 9.0, C**17**H), 5.39-5.40 (1H, m, C**4**H), 5.69 (1H, d, $^{4}J_{HH}$ 1.9, C**6**H). ^{13}C NMR ($CDCl_3$, 151 MHz): 13.50 (C**18**), 19.00 (C**19**), 21.24 (C**23**), 21.37 (C**11**), 22.99 (C**16**), 24.55 (C**15**), 24.93 (C**21**), 31.69 (C**2**), 31.87 (C**7**), 31.89 (C**8**), 33.93 (C**1**), 35.04 (C**10**), 38.96 (C**12**), 44.24 (C**13**), 48.02 (C**9**), 57.16 (C**14**), 63.83 (C**17**), 117.08 (C**6**), 123.81 (C**4**), 139.50 (C**5**), 147.18 (C**3**), 169.52 (C**22**), 209.64 (C**20**). m/z (ASAP): 357 (15%, $[M+H]^+$), 314 (92%, $[M+H-CH_3CO]^+$), 297 (56%, $[M-CH_3COO]^+$).

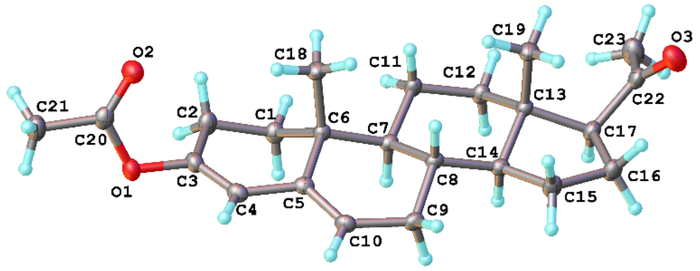

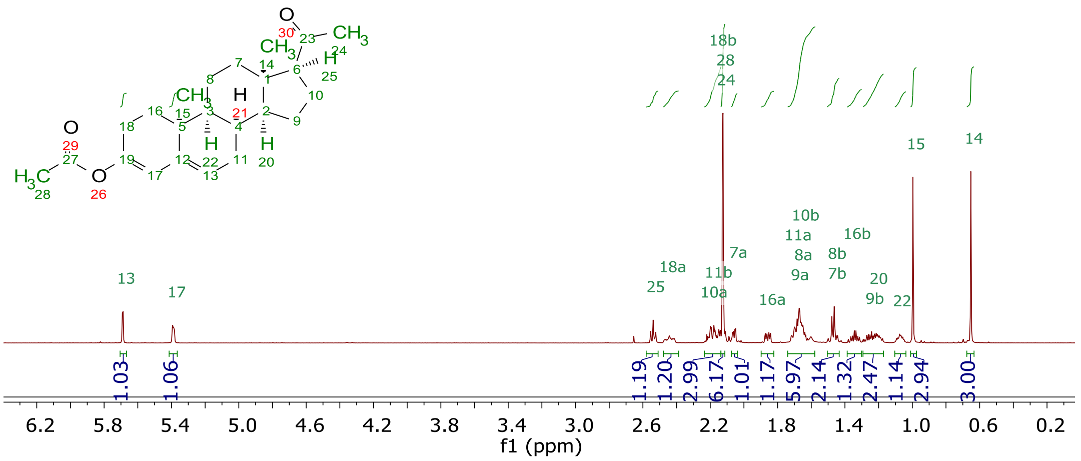

Figure 1: ^{1}H NMR spectrum of progesterone enol acetate.

2.1.2 6β-Fluoroprogesterone

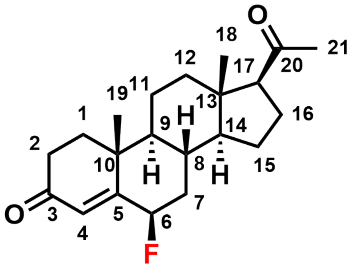

21-β

Progesterone enol acetate **17** (1.50 g, 4.2 mmol) was dissolved in a mixture of acetonitrile (20 mL) and acetone (30 mL). Selectfluor™ (1.56 g, 4.4 mmol) was added and the mixture was stirred at ambient temperature for 2 h. The solvents were removed *in vacuo*, and the residue was partitioned between ethyl acetate (30 mL) and water (25 mL). The organic layer was separated, washed with brine (15 mL) and dried over $MgSO_4$. The solvent was evaporated *in vacuo* and, after column chromatography, (silica, hexanes : ethyl acetate, 10:1 to 3:1, Rf.: 0.18 in 3:1 mixture) 6β-fluoroprogesterone (0.65 g, 46% yield) was isolated as a white solid. Crystals suitable for X-ray crystallographic analysis were obtained by slow evaporation of an acetone solution of 6β-fluoroprogesterone. M.p. 152-156 °C (lit.[4] 159-161 °C, from benzene). IR (cm^{-1}): 2932, 1700, 1682, 1386, 1355, 1228, 1193, 1161. ^{1}H NMR ($CDCl_3$, 600 MHz): 0.69 (3H, s, C**18**H_3), 0.99 (1H, td, J_{HH} 11.4, $^{3}J_{HH}$ 3.9, C**9**H), 1.10-1.27 (2H, m, C**7**H, C**14**H), 1.30 (3H, d, $^{5}J_{FH}$ 1.3, C**19**H_3), 1.30-1.34 (1H, m, C**15**H), 1.44 (1H, td, $^{2}J_{HH}$ 12.7, $^{3}J_{HH}$ 3.9 Hz, C**12**H), 1.48-1.54 (1H, m, C**11**H), 1.62-1.77 (4H, m, C**1**H, C**11**H, C**15**H, C**16**H), 1.90-1.94 (1H, m, C**8**H), 2.05-2.11 (2H, m, C**1**H, C**12**H), 2.12 (3H, s, C**21**H_3), 2.17-2.24 (2H, m, C**11**H, C**7**H), 2.43 (1H, dt, $^{2}J_{HH}$ 16.9,

$^{3}J_{HH}$ 3.3, C**2**H), 2.51-2.58 (2H, m, C**2**H, C**17**H), 4.99 (1H, dt, $^{2}J_{HF}$ 48.6, $^{3}J_{HH}$ 2.5, C**6**HF), 5.87 (1H, d, $^{4}J_{HF}$ 5.0, C**4**H). ^{19}F NMR ($CDCl_3$, 376 MHz): −165.5 (td, $^{2}J_{HF}$ 47.8, $^{3}J_{HF}$ 12.6). ^{13}C NMR ($CDCl_3$, 151 MHz): 13.45 (C**18**), 18.53 (C**19**), 20.98 (C**11**), 22.99 (C**16**), 24.37 (C**15**), 30.09 (C**8**), 31.59 (C**21**), 34.34 (C**2**), 37.04 (C**1**), 37.35 (d, $^{2}J_{FC}$ 23.5, C**7**), 37.97 (C**10**), 38.65 (C**12**), 44.11 (C**13**), 53.21 (C**9**), 55.99 (C**14**), 63.55 (C**17**), 93.41 (d, $^{1}J_{FC}$ 166.0, C**6**), 128.57 (C**4**), 161.67 (d, $^{2}J_{FC}$ 12.6, C**5**), 199.88 (C**3**), 209.19 (C**20**). m/z (ASAP): 333 (100%, $[M+H]^+$), 313 (98%, $[M-F]^+$).

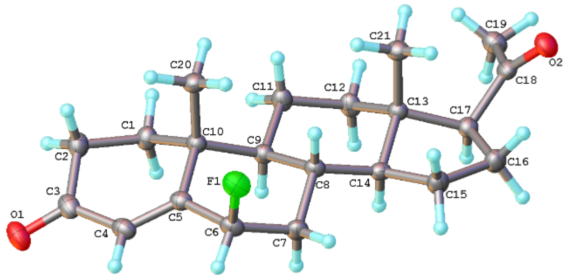

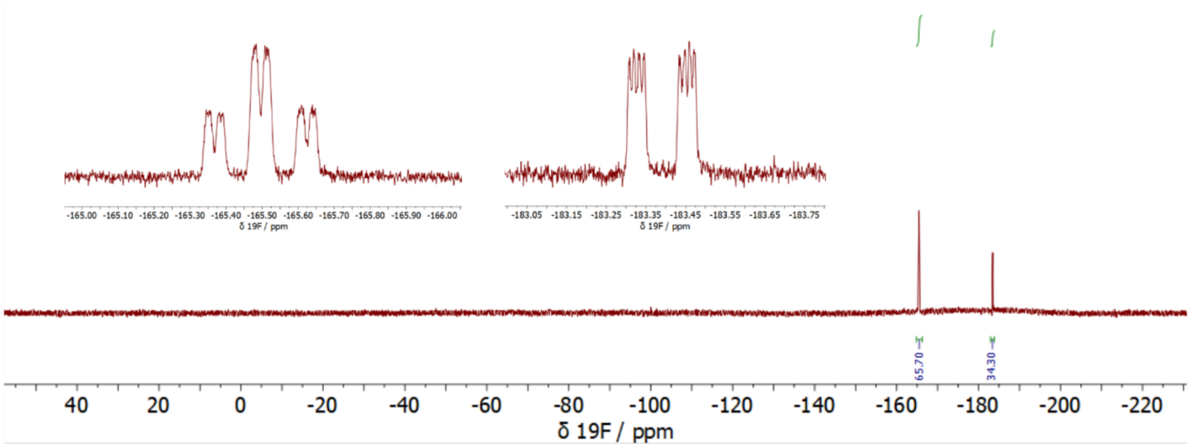

Figure 2: ^{19}F NMR spectrum of the crude fluorinated product.

2.1.3 6α-Fluoroprogesterone

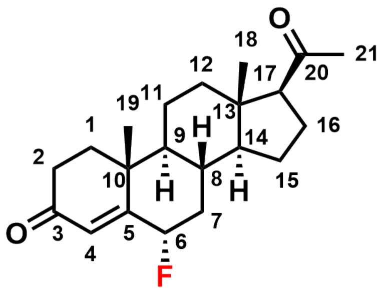

21-α

6β-Fluoroprogesterone **21-β** (0.65 g, 1.9 mmol) was dissolved in glacial acetic acid (25 mL) and dry HCl gas (generated using c. H_2SO_4 and NaCl) was bubbled into the solution for 1.5 h

(approx. 20 bubbles/min). The solvent was evaporated and the crude product (19:1 α:β) was recrystallized from methanol (5 mL) to give 6α-fluoroprogesterone (0.48 g, 74% yield) as colourless needles. M.p. 144-147 °C (lit.[4] 146-148 °C, from acetone-hexane). IR (cm^{-1}): 2948, 1700, 1680, 1357, 1269, 1225, 1186, 1059. ^{1}H NMR ($CDCl_3$, 600 MHz): 0.66 (3H, s, C**18**H_3), 0.99 (1H, m, C**9**H), 1.18 (3H, d, C**19**H_3), 1.23-1.33 (3H, m, C**7**H, C**14**H, C**15**H), 1.39-1.46 (2H, m, C**11**H, C**12**H), 1.56-1.62 (1H, m, C**8**H), 1.62-1.67 (1H, m, C**11**H), 1.67-1.76 (2H, m, C**15**H, C**16**H), 1.76-1.82 (1H, m, C**1**H), 2.03-2.10 (2H, m, C**1**H, C**12**H), 2.12 (3H, s, C**21**H_3), 2.16-2.22 (1H, m, C**16**H), 2.26-2.32 (1H, m, C**7**H), 2.35-2.39 (1H, m, C**2**H), 2.44 (1H, td, $^{2}J_{HH}$ 14.5, $^{3}J_{HH}$ 3.4, C**2**H), 2.54 (1H, t, $^{3}J_{HH}$ 8.5, C**17**H), 5.09 (1H, ddd, $^{2}J_{FH}$ 47.9, $^{3}J_{HH}$ 12.3, $^{3}J_{HH}$ 5.6, C**6**HF), 6.09 (1H, s, C**4**H). ^{19}F NMR ($CDCl_3$, 376 MHz): −183.37 (ddd, $^{2}J_{FH}$ 47.9, $^{3}J_{HH}$ 12.3, $^{3}J_{HH}$ 5.6, C**6**HF). ^{13}C NMR ($CDCl_3$, 151 MHz): 13.41 (C**18**), 18.20 (C**19**), 21.03 (C**11**), 22.96 (C**16**), 24.47 (C**15**), 31.60 (C**21**), 33.52 (C**8**), 33.83 (C**2**), 36.42 (C**1**), 38.48 (d, $^{2}J_{FC}$ 17.7, C**7**), 38.49 (C**12**), 39.24 (C**10**), 44.00 (C**13**), 53.56 (C**9**), 55.74 (C**14**), 63.41 (C**17**), 88.22 (d, $^{1}J_{FC}$ 183.9, C**6**), 119.88 (d, $^{3}J_{CF}$ 14.8 C**4**), 165.80 (d, $^{2}J_{FC}$ 11.2, C**5**), 198.79 (C**3**), 209.09 (C**20**). m/z (ASAP): 333 (100%, $[M+H]^+$).

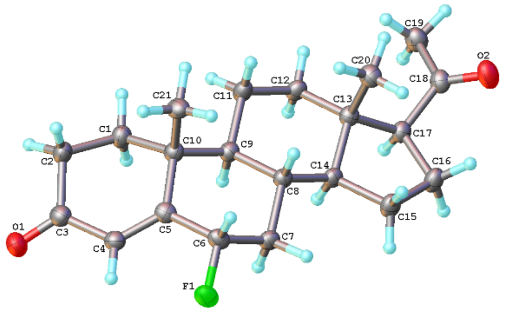

2.1.4 3-Ethoxy-pregna-3,5-dien-20-one

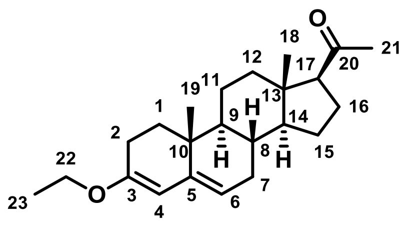

25

Method 1: Progesterone **13** (1.50 g, 4.76 mmol), triethylorthoformate (2.0 mL, 12 mmol) and *p*-toluenesulfonic acid (0.12 g, 0.64 mmol) were dissolved in ethanol (25 mL) then stirred at room temperature for 16 h. Triethylamine (2 mL) was added and the mixture was left at 0 °C for 1 h. The resulting solid was filtered and washed with 1% triethylamine in ethanol (10 mL)

then recrystallised from diethyl ether and dried *in vacuo* to give 3-ethoxy-pregna-3,5-dien-20-one (0.32 g, 20%) as a yellow solid.

Method 2:[5] Progesterone **13** (1.99 g, 6.34 mmol) was dissolved in anhydrous THF (20 mL) with triethyl orthoformate (2.6 mL, 16 mmol), ethanol (1.0 mL, 17 mmol) and *p*-toluenesulfonic acid (0.035 g, 0.20 mmol) under an argon atmosphere. The reaction mixture was heated to 45 °C for 2 h then quenched with 10% $NaHCO_3$ (20 mL) and extracted with diethyl ether (3 × 30 mL). The combined organic extracts were washed with brine (60 mL), dried over $MgSO_4$ and concentrated *in vacuo* to give the crude product as a yellow solid. Column chromatography over silica gel (hexane:EtOAc gradient 1:0 to 8:1) afforded 3-ethoxy-pregna-3,5-dien-20-one (1.06 g, 48%) as a white solid. M.p. 102-104 °C (lit.[6] 105-107 °C from MeOH/pyridine). ^{1}H NMR ($CDCl_3$; 400 MHz) 0.64 (3H, s, C**18**H_3), 0.97 (3H, s, C**19**H_3), 1.30 (3H, t, $^3J_{HH}$ 7.0, OCH_2C**H**$_3$), 2.12 (3H, s, C**21**H_3), 3.75 (2H, q, $^3J_{HH}$ 7.0, OC**H**$_2CH_3$), 5.10 (1H, d, J_{HH} 2.1, C**17**H), 5.20 (1H, dd, J_{HH} 5.0, J_{HH} 2.1). ^{13}C NMR ($CDCl_3$; 101 MHz) 13.49, 14.81, 19.09, 21.32, 22.93, 24.55, 25.65, 31.72, 31.84, 31.99, 33.98, 35.31, 39.00, 44.27, 48.30, 57.26, 62.33, 63.86, 99.06, 117.88, 141.10, 154.67, 209.79. HRMS (ESI+) *m/z* calc. for $C_{23}H_{35}O_2^+$ 343.2637; found 343.2645.

2.1.5 Testosterone enol diacetate

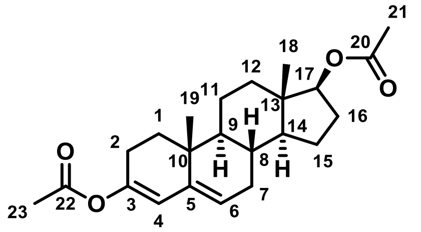

18

Testosterone **14** (0.644 g, 2.23 mmol) was dissolved in acetyl chloride (6 mL) and acetic anhydride (4 mL) then heated to 100 °C for 2 h before being allowed to cool to room temperature. The mixture was then concentrated *in vacuo* and the resulting solid washed with cold acetonitrile (2 × 10 mL) to give testosterone enol diacetate (0.578 g, 70%) as a white solid. M.p. 143-144 °C (lit.[7] 143-147 °C). ^{1}H NMR ($CDCl_3$; 400 MHz) 0.85 (3H, s, C**18**H_3), 1.03 (3H, s, C**19**H_3), 1.01-1.91 (12H, m), 2.06 (3H, s, C**17**-OAc), 2.08-2.11 (2H, m), 2.15 (3H, s, C**3**-OAc), 2.18-2.21 (2H, m), 2.42-2.47 (1H, m), 4.63 (1H, dd, $^3J_{HH}$ 9.2, $^3J_{HH}$ 7.7, C**17**H), 5.41 (1H, dd, $^3J_{HH}$ 5.3, $^4J_{HH}$ 2.3, C**6**H), 5.71 (1H, d, $^4J_{HH}$ 2.3, C**4**H). ^{13}C NMR ($CDCl_3$; 101 MHz) 12.19, 19.01,

20.84, 21.24, 21.33, 23.64, 24.92, 27.68, 31.54, 31.72, 33.90, 35.08, 36.86, 42.64, 48.05, 51.30, 82.86 (C**17**), 117.06 (C**6**), 123.67 (C**4**), 139.58 (C**5**), 147.20 (C**3**), 169.53 (C**3**-OAc), 171.39 (C**17**-OAc). HRMS (ESI+) *m/z* calc. for $C_{23}H_{33}O_4^+$ 373.2379; found 373.2379.

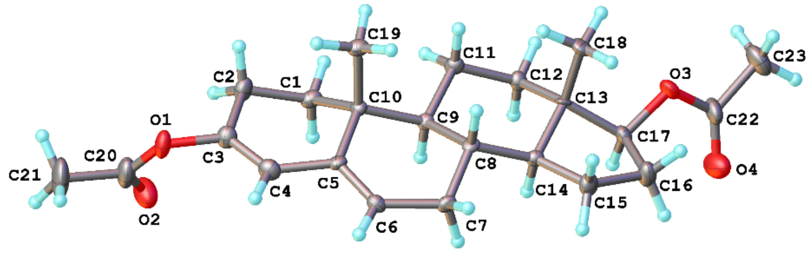

2.1.6 6-Fluorotestosterone acetate.

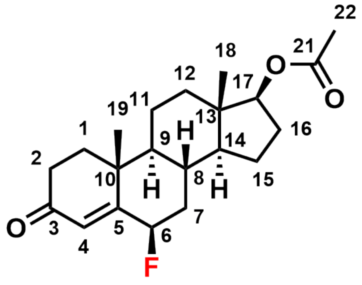

22-β

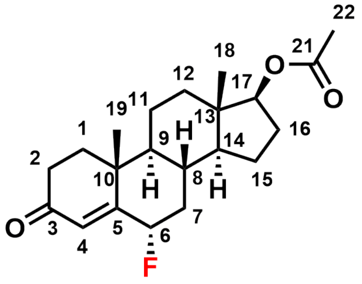

22-α

Testosterone enol diacetate **18** (0.103 g, 0.0275 mmol) and Selectfluor™ (0.109 g, 0.0308 mmol) were dissolve in acetonitrile (10 mL) and stirred at room temperature for 2 h. The solvent was removed *in vacuo* and the residue partitioned between ethyl acetate (20 mL) and water (20 mL). The organic layer was separated, washed with brine (20 mL), dried over $MgSO_4$ then concentrated *in vacuo* to give 6-fluorotestosterone acetate, (0.071 g, 74%), as a white solid. ^{1}H NMR ($CDCl_3$; 400 MHz) 0.83/0.86 (s, α/β-C**18**H_3), 1.19/1.30 (s, α/β-C**19**H_3), 2.04/2.05 (s, α/β-C**17**-OAc), 4.99 (dt, $^2J_{HF}$ 48.7, J_{HH} 3.8, β-C**6**H), 5.07 (dddd, $^2J_{HF}$ 47.7, $^3J_{HH}$ 12.2, $^3J_{HH}$ 5.9, $^4J_{HH}$ 2.0, α-C**6**H), 5.87 (dd, $^4J_{HF}$ 3.8, $^4J_{HH}$ 1.0, β-C**4**H), 6.08 (dt, $^4J_{HF}$ 2.0, $^4J_{HH}$ 1.0, α-C**4**H). ^{19}F NMR ($CDCl_3$; 376 MHz) −183.41 (ddd, $^2J_{HF}$ 47.7, $^3J_{HF}$ 9.3, $^3J_{HF}$ 3.8, α-F), −165.51 (tdd, $^2J_{HF}$ 48.7, $^3J_{HF}$ 11.9, $^3J_{HF}$ 2.8, β-F). ^{13}C NMR ($CDCl_3$; 101 MHz) 36.48 (d, $^2J_{CF}$ 24.5, α/β-C**7**), 36.96 (d, $^2J_{CF}$ 23.5, α/β-C**7**), 82.29/82.45 (α/β-C**17**), 88.23 (d, $^1J_{CF}$ 185.2, α/β-C**6**), 93.36 (d, $^1J_{CF}$ 166.7, α/β-C**6**), 128.56/128.65 (α/β-C**4**), 161.70 (d, $^2J_{CF}$ 12.2, α/β-C**5**), 165.83 (d, $^2J_{CF}$ 11.3, α/β-C**5**), 171.21/171.31 (α/β-C**17**-OAc), 198.75/199.94 (α/β-C**3**). HRMS (ESI+) *m/z* calc. for $C_{21}H_{30}O_3F$ 349.2179; found 349.2175.

X-ray crystal structure, co-crystal of both enantiomers: The carbon atoms of disordered (0.65:0.35) CHF group in structure **22** were refined in isotropic approximation.

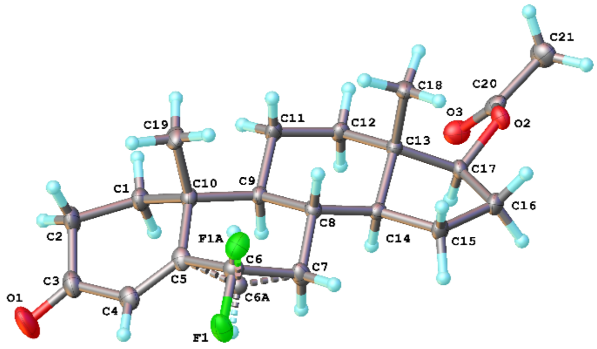

2.1.7 (+)-4-Cholesten-3-one enol acetate

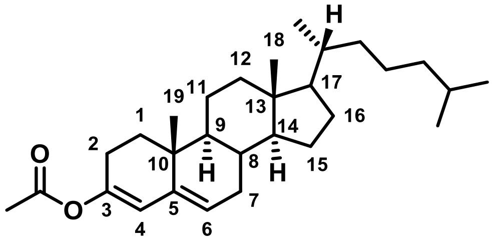

19

(+)-4-Cholesten-3-one **15** (0.474 g, 1.23 mmol) was dissolved in acetyl chloride (6 mL) and acetic anhydride (4 mL) then heated to 100 °C for 2 h before being allowed to cool to room temperature. The mixture was then concentrated *in vacuo* and the resulting solid washed with cold acetonitrile (2 × 10 mL) to give (+)-4-cholesten-3-one enol acetate (0.305 g, 58%) as a white solid. M.p. 81-82 °C (lit.[8] 80-81 °C). ^{1}H NMR ($CDCl_3$; 400 MHz) 0.70 (3H, s, C**18**H_3), 0.82-09.94 (10H, m), 1.00 (3H, s, C**19**H_3), 1.01-1.74 (15H, m), 1.79-1.92 (3H, m), 1.97-2.11 (6H, m), 2.13 (3H, s, C**3**-OAc), 2.43 (1H, m, C**17**H), 5.39 (1H, m, C**6**H), 5.68 (1H, d, $^{4}J_{HH}$ 2.3, C**4**H). ^{13}C NMR ($CDCl_3$; 101 MHz) 11.98, 18.72, 18.86, 21.12, 21.22, 22.57, 22.83, 23.83, 24.20, 24.82, 28.03, 28.25, 31.75, 31.88, 33.78, 34.90, 35.80, 36.18, 39.52, 39.75, 42.45, 47.97, 56.14, 56.85, 117.03 (s, C**6**), 124.13 (C**4**), 139.37 (C**5**), 146.97 (C**3**), 169.43 (OAc). Calc: %C 81.63, %H 10.87, %N 0; measured: %C 79.55, %H 10.61, %N −0.03.

2.1.8 (+)-6-Fluoro-4-cholesten-3-one.

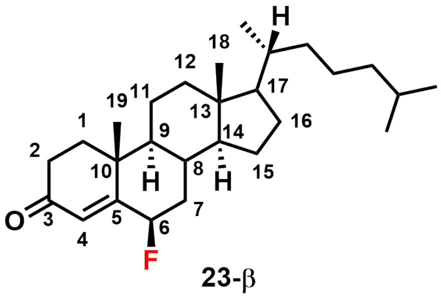

23-β

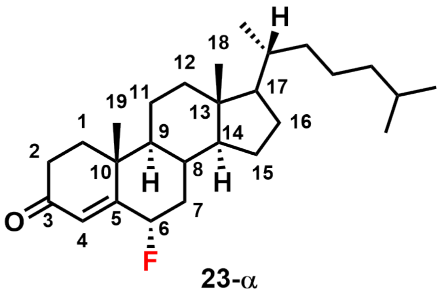

23-α

(+)-4-Cholesten-3-one enol acetate **19** (0.279 g, 0.0652 mmol) and Selectfluor™ (0.292 g, 0.0801 mmol) were dissolved in acetonitrile (10 mL) and stirred at room temperature for 2 h. The solvent was removed *in vacuo* and the residue was partitioned between ethyl acetate (20 mL) and water (20 mL). The organic layer was separated, washed with brine (20 mL), dried over $MgSO_4$ then concentrated *in vacuo* to give (+)-6-fluoro-4-cholesten-3-one, (0.217 g, 78%), as a yellow solid. ^{1}H NMR ($CDCl_3$; 400 MHz) 0.70/0.73 (s, α/β-C**18**H_3), 1.19/1.27 (s, α/β-C**19**H_3), 4.97 (dt, $^2J_{HF}$ 48.2, $^3J_{HH}$ 2.8, β-C**6**H), 5.14 (dddd, $^2J_{HF}$ 48.6 $^3J_{HH}$ 12.2, $^3J_{HH}$ 5.9, $^4J_{HH}$ 2.0, α-C**6**H), 5.86 (dd, $^4J_{HF}$ 4.8, $^4J_{HH}$ 0.9, β-C**4**H), 6.06-6.07 (m, α-C**4**H). ^{19}F NMR ($CDCl_3$; 376 MHz) −183.06 (ddd, $^2J_{HF}$ 48.6, $^3J_{HF}$ 9.7, $^3J_{HF}$ 4.8, α-F), −165.27 (tdd, $^2J_{HF}$ 48.2, $^3J_{HF}$ 12.1, $^3J_{HF}$ 2.9, β-F). ^{13}C NMR ($CDCl_3$; 101 MHz) 36.12 (d, $^2J_{CF}$ 14.6, α/β-C**7**), 37.34 (d, $^2J_{CF}$ 23.4, α/β-C**7**), 88.36 (d, $^1J_{CF}$ 185.0, α/β-C**6**), 93.46 (d, $^1J_{CF}$ 166.3, α/β-C**6**), 162.19 (d, $^2J_{CF}$ 12.3, α/β-C**5**), 166.42 (d, $^2J_{CF}$ 11.1, α/β-C**5**), 198.87/200.05 (α/β-C**3**).

2.1.9 Hydrocortisone enol tetraacetate

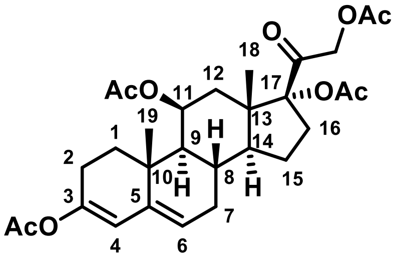

20

Hydrocortisone **16** (0.811 g, 2.24 mmol) was dissolved in acetyl chloride (6.0 mL) and acetic anhydride (4.0 mL) then heated to 100 °C for 2 h before being allowed to cool to room temperature. The mixture was then concentrated *in vacuo* and the resulting solid washed with cold acetonitrile (2 × 10 mL) to give hydrocortisone enol tetraacetate (0.670 g, 56%) as a white solid. M.p. 207-213 °C (lit.[9] 211-214 °C). ^{1}H NMR ($CDCl_3$; 400 MHz) 0.86 (3H, s, C**18**H_3),

1.06 (3H, s, C**19**H_3), 1.24 (2H, t, J_{HH} 7.0), 1.26-1.55 (2H, m), 1.71-2.50 (10H, m), 2.03 (3H, s, OAc), 2.09 (3H, s, OAc), 2.13 (3H, s, OAc), 2.15 (3H, s, OAc), 2.84-2.91 (1H, m), 3.68-3.76 (1H, m), 4.60-4.86 (2H, m), 5.30-5.34 (1H, m), 5.53-5.57 (1H, m, C**6**H), 5.66 (1H, d, $^4J_{HH}$ 2.2, C**4**H). ^{13}C NMR ($CDCl_3$; 101 MHz) 15.88, 20.64, 21.23, 21.35, 21.44, 22.01, 23.97, 24.62, 28.75, 30.87, 31.70, 33.54, 34.79, 36.00, 46.92, 50.14, 53.31, 67.03, 69.73, 94.69, 116.17 (C**6**), 122.54 (C**4**), 140.39 (C**5**), 147.41 (C**3**), 169.45 (OAc), 170.19 (OAc), 170.36 (OAc), 170.92 (OAc), 198.92 (C**17**). HRMS (ESI+) *m/z* calc. for $C_{29}H_{39}O_9^+$ 531.2594; found 531.2595.

2.1.10 6-Fluorohydrocortisone triacetate

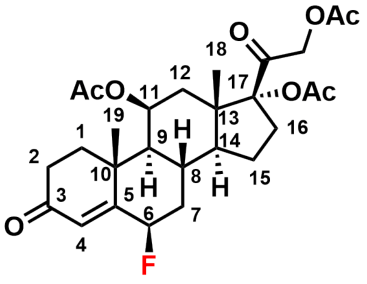

24-β

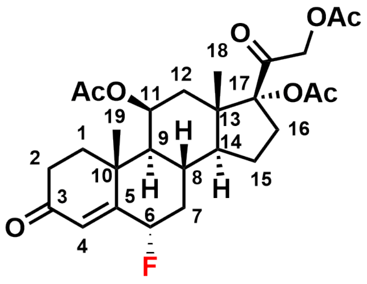

24-α

Hydrocortisone enol tetraacetate **20** (0.084 g, 0.157 mmol) and Selectfluor™ (0.060 g, 0.176 mmol) were dissolved in acetonitrile (10 mL) and stirred at room temperature for 2 h. The solvent was removed *in vacuo* and the residue was partitioned between ethyl acetate (20 mL) and water (20 mL). The organic layer was separated, washed with brine (20 mL), dried over $MgSO_4$ then concentrated *in vacuo* to give 6-fluoro-hydrocortisone, (0.045 g, 69%), as a white solid. ^{1}H NMR ($CDCl_3$; 400 MHz) 0.86/0.89 (s, α/β-C**18**H_3), 1.25/1.35 (s, α/β-C**19**H_3), 2.02/2.03 (s, α/β-OAc), 2.06/2.07 (s, α/β-OAc), 2.14 (s, OAc), 5.00 (dt, $^2J_{HF}$ 48.6, $^3J_{HH}$ 2.6, β-C**6**H), 5.16 (dddd, $^2J_{HF}$ 48.6, $^3J_{HH}$ 12.6, $^3J_{HH}$ 6.2, $^4J_{HH}$ 2.1, α-C**6**H), 5.83 (d, $^4J_{HF}$ 4.6, β-C**4**H), 6.01 (m, α-C**6**H). ^{19}F NMR ($CDCl_3$; 376 MHz) −184.54 (ddd, $^2J_{HF}$ 48.6, $^3J_{HF}$ 10.7, $^3J_{HF}$ 3.9, α-F), −165.83 (tdd, $^2J_{HF}$ 48.6, $^3J_{HF}$ 11.7, $^3J_{HF}$ 4.6, β-F). ^{13}C NMR ($CDCl_3$; 101 MHz) 37.84 (d, $^2J_{CF}$ 23.8, α/β-C**7**), 38.69 (d, $^2J_{CF}$ 18.7, α/β-C**7**), 87.38 (d, $^1J_{CF}$ 184.8, α/β-C**6**), 92.37 (d, $^1J_{CF}$ 168.3, α/β-C**6**), 161.34 (d, $^2J_{CF}$ 12.1, α/β-C**5**), 165.55 (d, $^2J_{CF}$ 11.5, α/β-C**5**), 169.71/169.93 (α/β-OAc), 170.34/170.37 (α/β-OAc), 170.75/170.78 (α/β-OAc), 198.14/198.71 (α/β-C**17**), 198.76/199.31 (α/β-C**3**).

2.1.11 Reactions of 3-ethoxy-pregna-3,5-dien-20-one 25 with N–F reagents

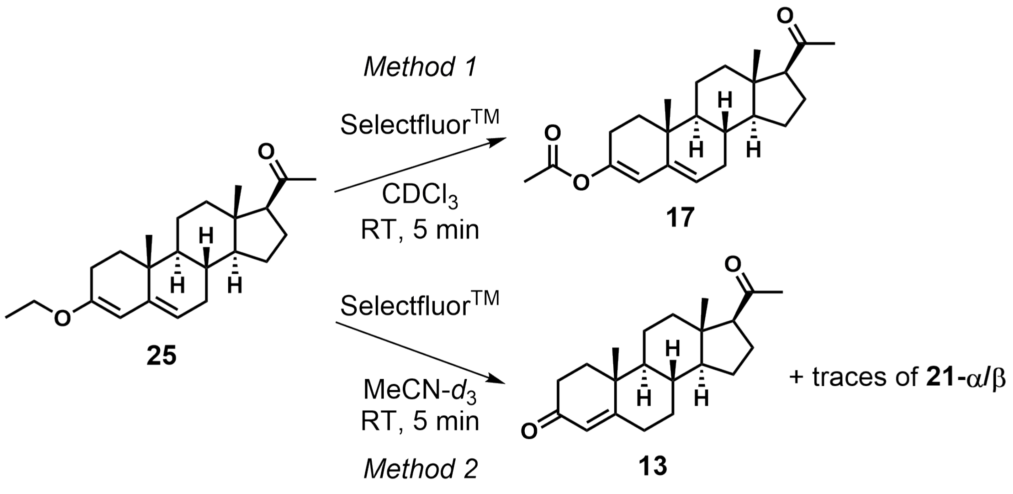

Method 1: 3-Ethoxy-pregna-3,5-dien-20-one **25** (10.5 mg) was dissolved in $CDCl_3$ and Selectfluor™ (0.95 equiv) was added. The HR-MS shown in **Figure 3** corresponds to the product of this reaction, progesterone enol acetate **17**.

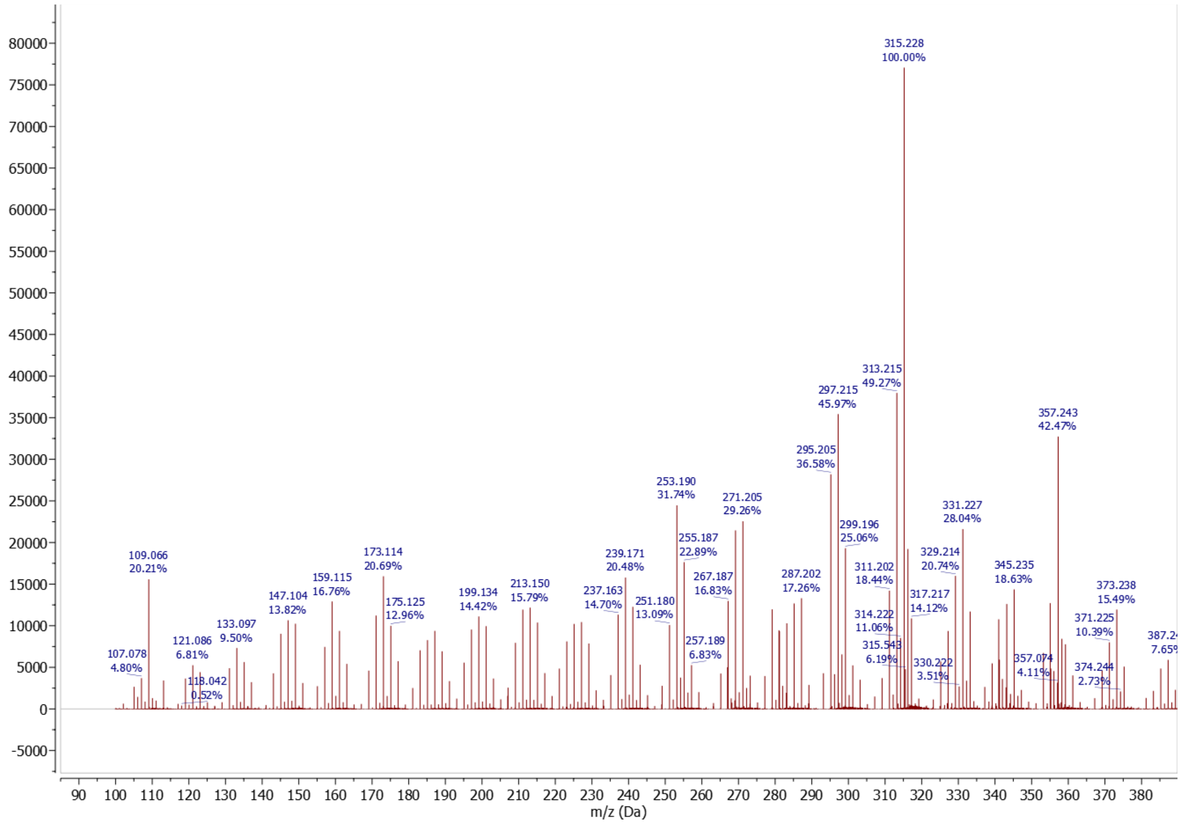

Figure 3: High-resolution mass spectrum of product from reaction of **25** with Selectfluor™ (0.95 equiv.) in $CDCl_3$.

Method 2: 3-Ethoxy-pregna-3,5-dien-20-one **25** (11.5 mg) was dissolved in MeCN-d_3 and Selectfluor™ (1.0 equiv.) was added. NMR spectra were acquired after 5 min (**Figure 4**). ^{1}H NMR spectra showed the formation of progesterone **13** rather than progesterone enol acetate **17**.

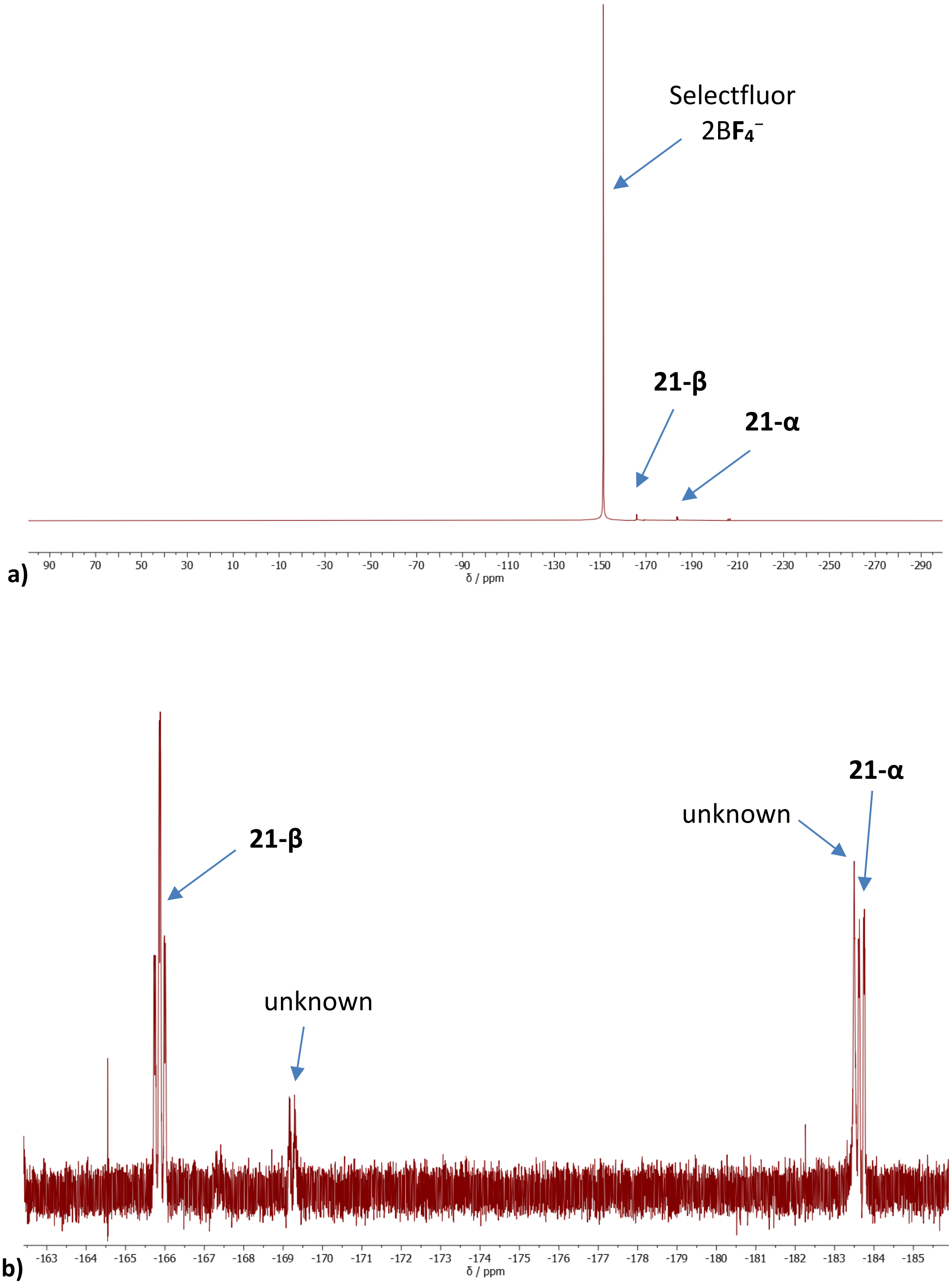

Figure 4: ^{19}F NMR spectra corresponding to reaction of **25** with Selectfluor™ (1.0 equiv.) in MeCN-d_3.

2.1.12 Attempted preparation of protonated $ClCH_2$-DABCO$^+$ BF_4^- ('spent' Selectfluor™)

To probe the possibility of epimerisation of β-fluoroprogesterone by protonated 'spent' Selectfluor™, we attempted to prepare protonated $ClCH_2$-DABCO$^+$ BF_4^- using 2 methods:

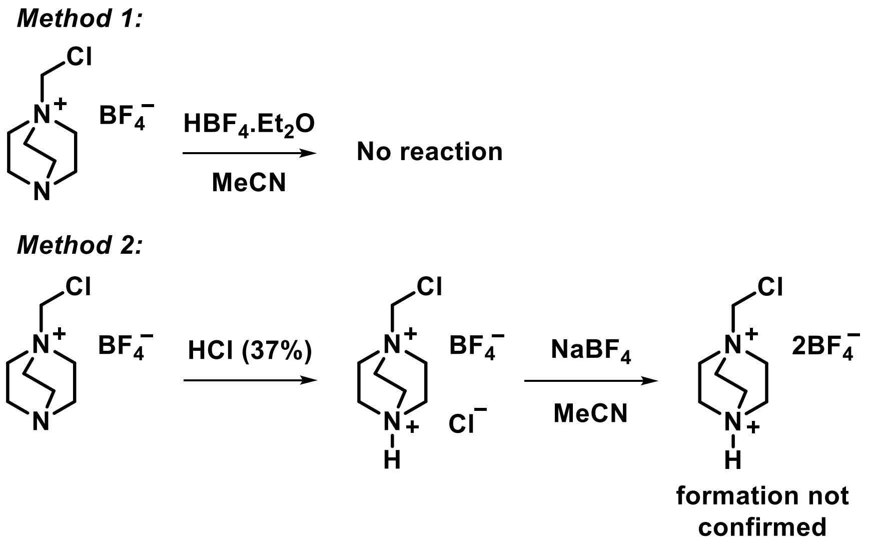

Method 1: $ClCH_2$-DABCO$^+$ BF_4^- (10 mg), prepared using our previously reported method,[2] was dissolved in MeCN (1 mL) and a commercial sample of $HBF_4.Et_2O$ (brown viscous appearance, 1 mL) was added to the solution. The mixture was stirred at RT for 1 h before evaporation of solvents. NMR spectroscopy of the brown residue from the reaction gave a complex mixture of products, most likely due to the impurities present in the commercial $HBF_4.Et_2O$.

Method 2: $ClCH_2$-DABCO$^+$ BF_4^- (10 mg) was dissolved in HCl solution (37%, 2 mL). The mixture was stirred at RT for 3 h before evaporation of solvents. Counterion exchange was then attempted using $NaBF_4$. The product from the first step was dissolved in MeCN (5 mL) and $NaBF_4$ (15 mg) was added to the solution, which was stirred at RT for 2 days. The solvent was then evaporated. Although ^{1}H NMR spectroscopy of the product suggested that the dication was present, as indicated by higher shift values (**Figure 5**), we were unable to prove whether anion exchange had been effective. This was critical to ascertain because the presence of chloride ion would be likely to have a marked effect on kinetics, owing to its basicity in MeCN in comparison to the tetrafluoroborate ion.

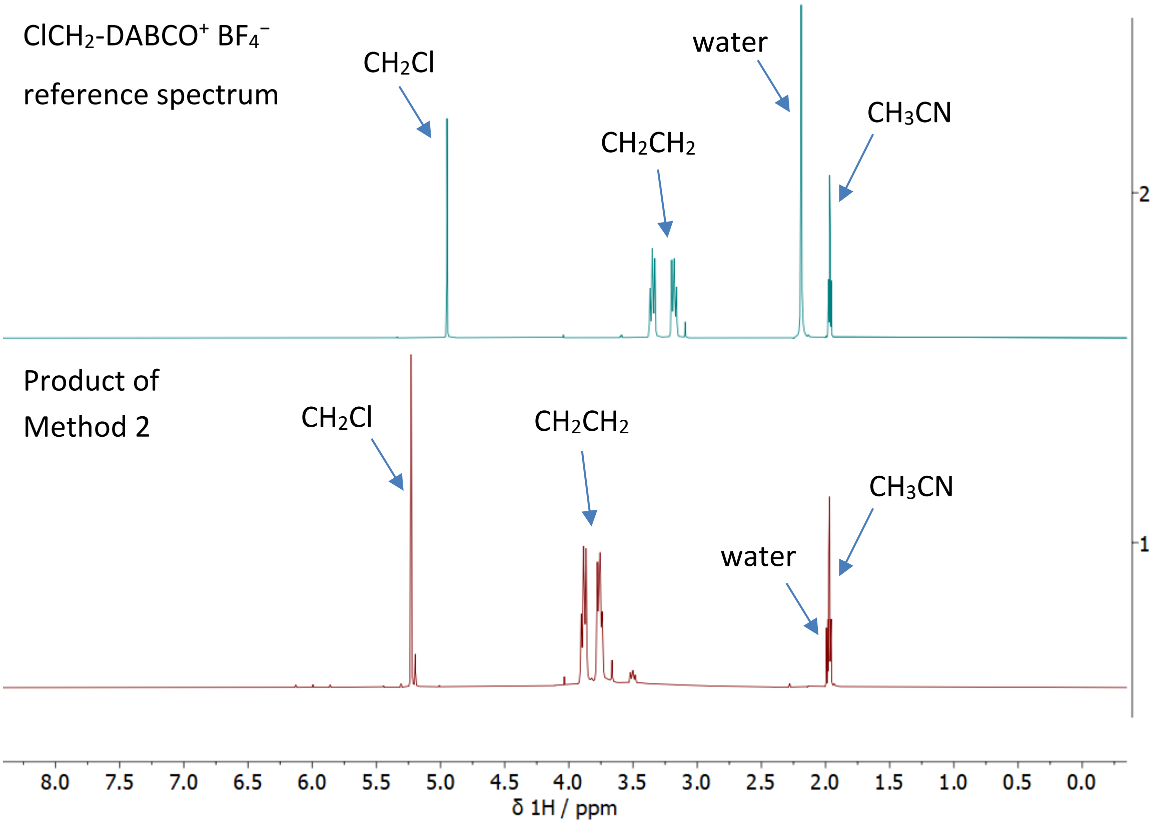

Figure 5: Spectrum 1: ^{1}H NMR spectrum of the product obtained from Method 2 in MeCN-d_3. Spectrum 2: $ClCH_2$-DABCO$^+$ BF_4^- in MeCN-d_3.

2.2 X-ray crystallography

The X-ray single crystal data have been collected using λMoKα (λ = 0.71073 Å, compounds **18** and **22**) and λCuKα (λ = 1.54178 Å, compounds **17**, **21α** and **21β**) radiation on a Bruker D8Venture (Photon100 CMOS detector, IμS-microsource, focusing mirrors) diffractometer equipped with a Cryostream (Oxford Cryosystems) open-flow nitrogen cryostat at 120.0(2) K. All structures were solved by direct method and refined by full-matrix least squares on F^2 for all data using Olex2 [1] and SHELXTL [2] software. All non-hydrogen atoms were refined anisotropically, hydrogen atoms were refined isotropically; hydrogen atoms in structures **18** and **22** were placed in the calculated positions and refined in riding mode. The carbon atoms of disordered (0.65:0.35) CHF group in structure **22** were refined in isotropic approximation.

The absolute configurations of the studied compounds were determined from anomalous scattering by calculating the Flack (*x*) [3] and Hooft (*y*) [4] parameters which should be equal to 0 for the correct absolute structure and 1 for the inverted model. The standard uncertainties of both parameters for compounds **18** and **22** were too large to regard the absolute configuration as unequivocal and in these cases the assignment was made based on chemical information.

Crystal data and parameters of refinement are listed in **Table 1**. Crystallographic data for the structures have been deposited with the Cambridge Crystallographic Data Centre as supplementary publications CCDC-1985095-1985099.

---

1. O. V. Dolomanov, L. J. Bourhis, R. J. Gildea, J. A. K. Howard and H. Puschmann, *J. Appl. Cryst.* **2009**, *42*, 339-341.

2. G.M. Sheldrick, *Acta Cryst.* **2008**, *A64*, 112-122.

3. H. D. Flack, *Acta Crystallogr., Sect. A* **1983**, *39*, 876–881.

4. R. W. W. Hooft, L. H. Straver, A. L. Spek, *J. Appl. Crystallogr.* **2008**, *41*, 96–103.

Table 1: Crystal data and structure refinement parameters.

Compound	**17**	**18**	**21α**	**21β**	**22**
Empirical formula	$C_{23}H_{32}O_3$	$C_{23}H_{32}O_4$	$C_{21}H_{29}O_2F$	$C_{21}H_{29}O_2F$	$C_{21}H_{29}FO_3$
Formula weight	356.48	372.48	332.44	332.44	348.44
Temperature/K	120.0	120.0	120.0	120	120.0
Crystal system	monoclinic	monoclinic	orthorhombic	monoclinic	orthorhombic
Space group	$P2_1$	$P2_1$	$P2_12_12_1$	$P2_1$	$P2_12_12_1$
a/Å	9.3411(7)	9.2419(13)	8.4307(4)	10.7502(4)	7.9436(4)
b/Å	7.5666(5)	7.5226(11)	10.3350(5)	6.0734(2)	12.5895(6)
c/Å	13.7556(10)	29.452(4)	20.3550(10)	14.0002(5)	17.9887(8)
α/°	90	90	90.00	90.00	90
β/°	94.038(3)	97.159(6)	90.00	104.6887(14)	90
γ/°	90	90	90.00	90.00	90
Volume/Å^3	969.84(12)	2031.6(5)	1773.56(15)	884.20(5)	1798.98(15)
Z	2	4	4	2	4
ρ_{calc}g/cm^3	1.221	1.218	1.245	1.249	1.287
μ/mm^{-1}	0.619	0.082	0.680	0.681	0.091
F(000)	388.0	808.0	720.0	360.0	752.0
Crystal size/mm^3	0.23 × 0.19 × 0.07	0.32 × 0.12 × 0.02	0.49 × 0.15 × 0.15	0.34 × 0.19 × 0.11	0.42 × 0.08 × 0.07
Radiation	CuKα	MoKα	CuKα	CuKα	MoKα
2Θ range for data collection/°	6.442 to 146.948	4.442 to 51.998	8.68 to 147.98°	8.5 to 145.86°	4.528 to 56.996
Reflections collected	12155	22948	12769	5515	34890
Independent reflections, R_{int}	3651, 0.0288	7988, 0.0736	3427, 0.0550	3071, 0.0187	4562, 0.0895
Data/restraints/parameters	3651/1/363	7988/1/496	3427/0/333	3071/1/333	4562/1/307

Goodness-of-fit on F^2	1.030	1.030	1.036	1.061	1.063
Final R_1 indexes [I≥2σ (I)]	0.0287	0.0898	0.0541	0.0296	0.0460
Final wR_2 indexes [all data]	0.0791	0.2550	0.1471	0.0781	0.1135
Largest diff. peak / hole / e $Å^{-3}$	0.23/-0.15	0.43/-0.32	0.25/-0.22	0.20/-0.16	0.24/-0.19
Flack parameter, x	-0.09(8)	-2.3(10)	-0.22(17)	-0.01(13)	-0.2(7)
Hooft parameter, y	-0.11(6)	-1.4(11)	-0.01(8)	-0.02(5)	-0.9(6)

2.3 Direct fluorination using fluorine gas

2.3.1 Direct fluorination of progesterone enol acetate in formic acid

Progesterone enol acetate **17** (1.07 g, 3.0 mmol) was dissolved in formic acid (98%, 30 mL) in a 100 mL glass fluorination reactor and cooled to 0-5 °C in a water bath. The solution was purged with nitrogen for 5 min (20 mL min^{-1}) then fluorine (10% *v/v* in nitrogen, 20 mL min^{-1}) was introduced for 50 min (1.4 equivalents); the initially colourless solution turned yellow. After the fluorination, the vessel was purged with nitrogen, the contents were transferred to a round bottomed flask and evaporated to dryness to leave a viscous yellow oil (1.02 g). The product mixture was dissolved in acetonitrile (25 mL) in a volumetric flask, a 1 mL aliquot was diluted twenty-fold and analysed by a calibrated HPLC-UV method.

Analysis by NMR spectroscopy showed that **21-α** and **21-β** were present in a 1:2 ratio. The HPLC-UV chromatogram of the crude product mixture showed only the presence of progesterone **13**, 6α-fluoroprogesterone (**21-α**) and 6β-fluoroprogesterone (**21-β**) as main components along with some minor impurities. However, integration of the chromatogram revealed that only half of the crude product mass could be accounted for by these three compounds. Column chromatography of the crude product led to the recovery of a fraction that contained a small amount of 6α- and 6β-fluoroprogesterone along with several unidentified non-fluorinated species and many other fractions that contained unidentified steroid derivatives. This outcome is consistent with the low mass recovery of the expected products that were observed by HPLC analysis. Progesterone **13** probably formed as a result of the acidic nature of the reaction medium, leading to the hydrolysis of progesterone enol acetate **17**.

2.3.1.1 HPLC-UV analysis: calibration

A Waters C18 column using water and acetonitrile as mobile phase with 0.1% formic acid additive was employed. Base-line separation was achieved using the following method: after injection, the sample was eluted at 1.5 ml min^{-1} flow rate for 3 min with a mixture of 60% water (0.1% HCOOH) and 40% acetonitrile (0.1% HCOOH) then the acetonitrile was increased to 95% over 15 min and this was maintained for a further 3 min. The UV detection was carried out at 237 nm, as all desired compounds have strong absorption at this wavelength. To accurately determine the amount of the desired products in a crude reaction mixture,

calibration curves were measured for the three known components. The concentration range where these compounds can be accurately measured with HPLC was between 0.1 and 0.5 mg mL^{-1} and so calibration was carried out in this range using 5 data points for each component. All three calibrations gave excellent linear relationships ($R^2 \geq 0.99$) between product concentration and absorption output, and they were validated by analysing an artificial sample of known concentration of each component which confirmed the accuracy of the method.

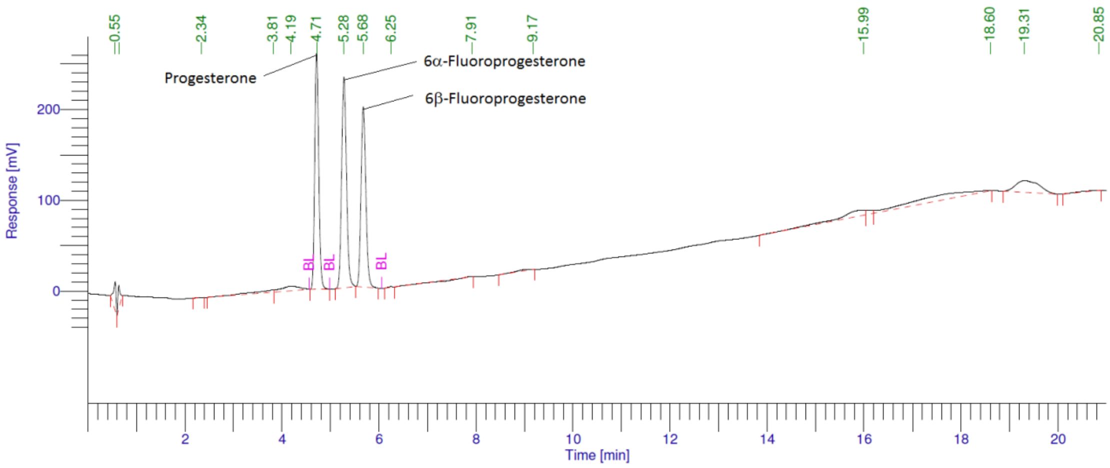

Figure 6: HPLC-UV chromatogram of a mixture of progesterone, 6α- and 6β-fluoroprogesterone for calibration.

Table 2: Validation results of the calibration curves.

**Compound**	**Concentration / mg mL^{-1}**	**Measured concentration / mg mL^{-1} (average of 5 injections)**
Progesterone	0.3370	0.3197 ± 0.0090
6α-Fluoroprogesterone	0.3232	0.3059 ± 0.0128
6β-Fluoroprogesterone	0.3021	0.2953 ± 0.0210

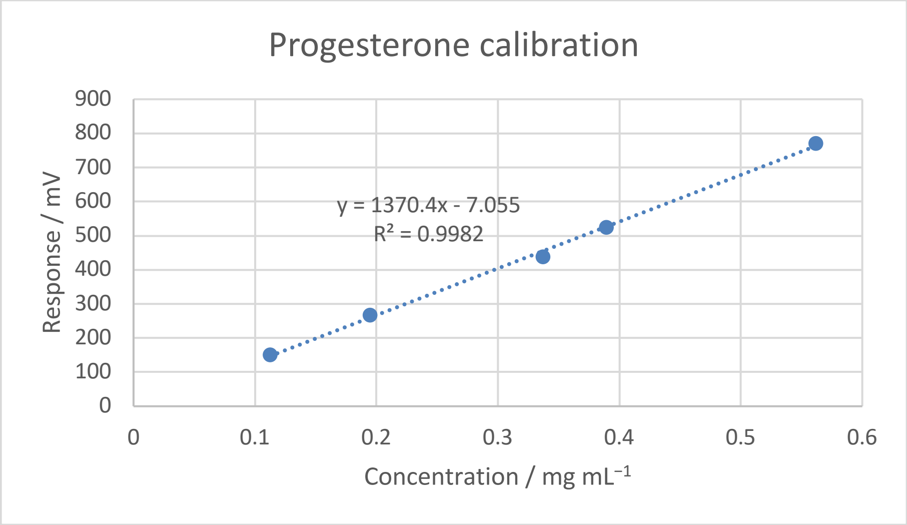

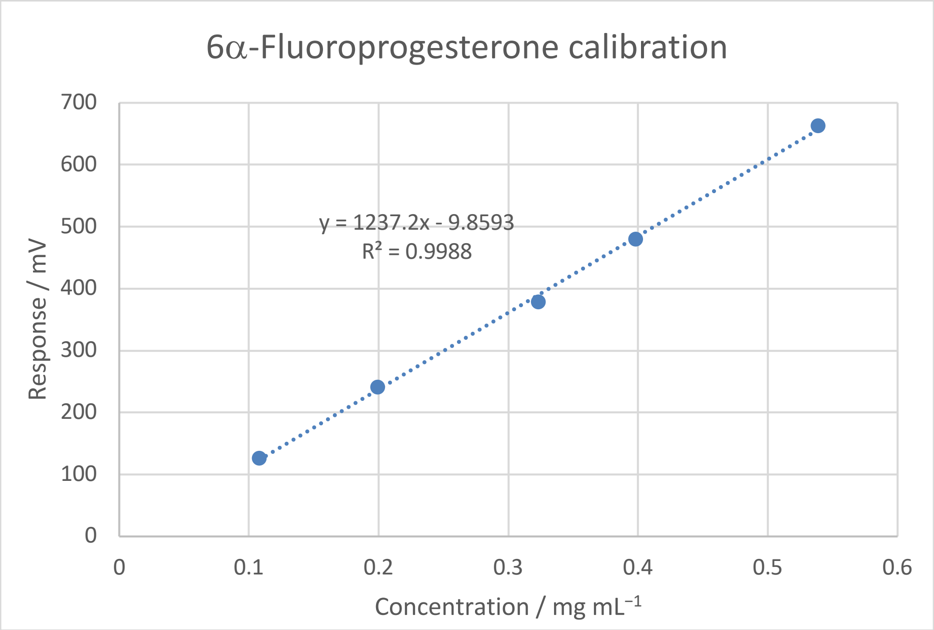

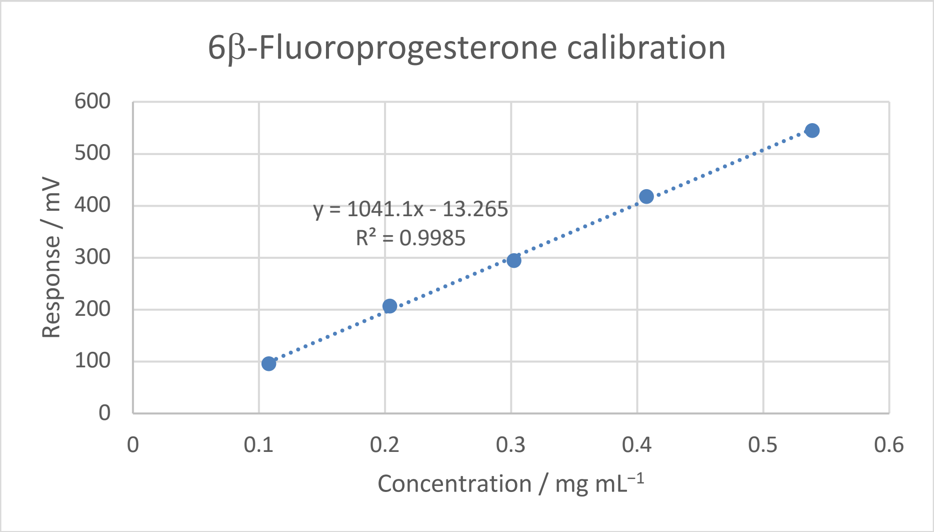

Figure 7: Calibration curves for the determination of progesterone, 6α- and 6β-fluoroprogesterone.

2.3.1.2 HPLC-UV analysis: crude product from direct fluorination:

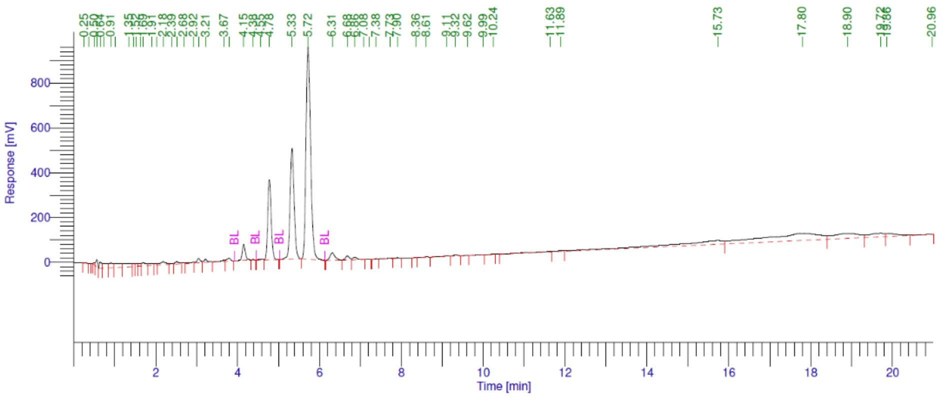

Figure 8: HPLC-UV chromatogram of the crude product from the direct fluorination of progesterone enol acetate.

Table 3: Quantitative analysis of the reaction products by HPLC-UV.

Product	Retention time / min	Calculated amount / mg
6α-Fluoroprogesterone	4.78	80.1
6β-Fluoroprogesterone	5.33	158.2
Progesterone	5.72	272.9

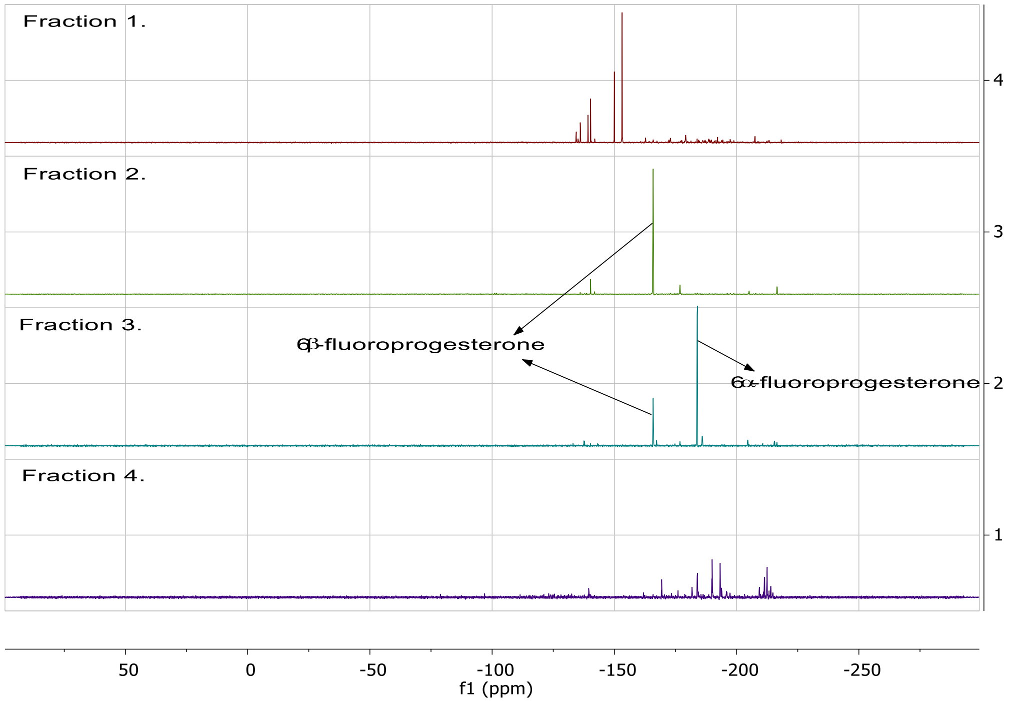

Figure 9: ^{19}F NMR spectra of the various fractions from the purification of a progesterone enol acetate fluorination mixture using column chromatography.

2.3.2 Direct fluorination of progesterone enol acetate in acetonitrile

Progesterone enol acetate **17** (0.18 g, 0.5 mmol) was added to a Simax bottle and dissolved in acetonitrile (20 mL). The reaction vessel was cooled to 0 °C, stirred rapidly and purged with nitrogen for 10 min. Fluorine gas (10% *v/v* in nitrogen) was then added at a prescribed flow rate (5 or 10 mL min^{-1}, 13-49 min) that was controlled by a mass flow controller. No colour change was observed. After purging with nitrogen for 20 min, the reaction vessel was removed and the solvent was removed under vacuum to give yellow crystals, yellow oil or a mixture of both. NMR analyses showed that **21-α** and **21-β** were present in a 38:62 ratio. The signal at δ = −150 ppm, which was present in all spectra, is due to fluorinated borosilicate glass, and is likely to have formed from the presence of HF in the crude reaction mixtures. Upon standing for 1-2 days, the crude samples containing crystalline products gradually showed the appearance of oily material, hence, the presence of small quantities of HF (formed as a by-product in reactions involving F_2) are likely to have caused fluorination of **17** or decomposition of **21α/β** to other fluorinated steroid derivatives.

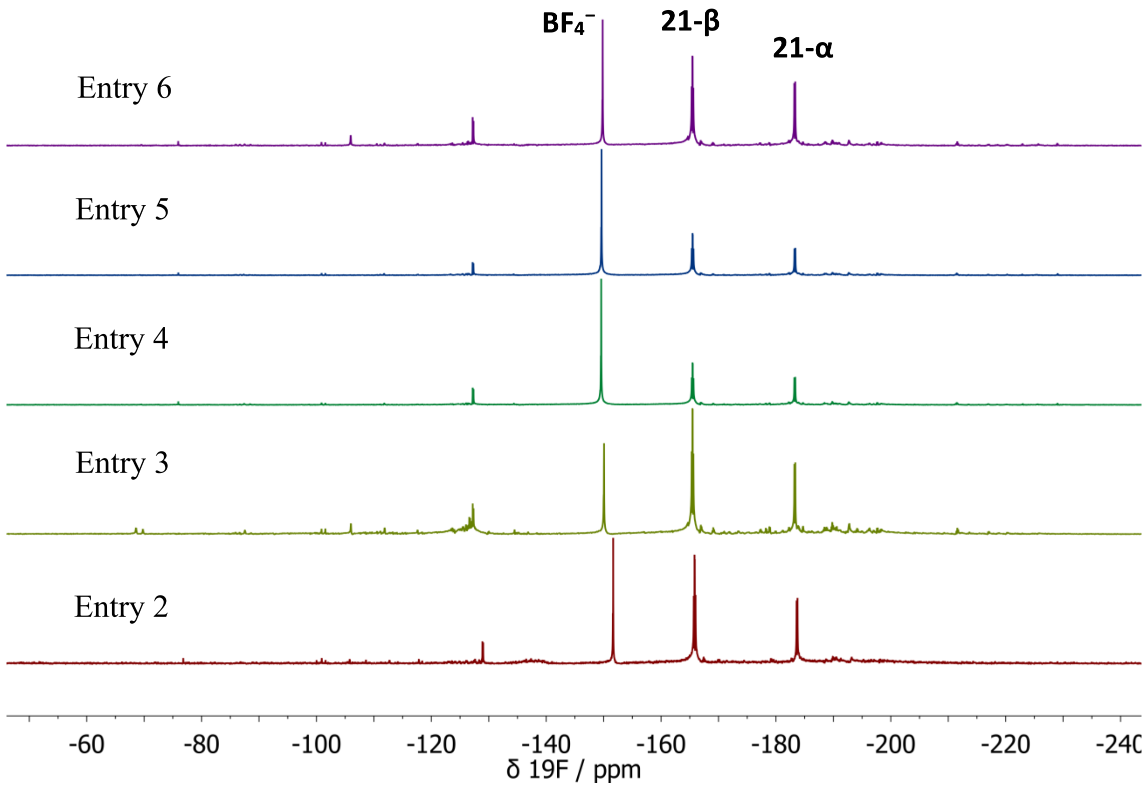

Figure 10: ^{19}F NMR spectra for crude products from fluorination of progesterone enol acetate **17** by F_2 in MeCN under the conditions summarised in **Table 4**.

Upon evaporation of solvents, analyses of the crude product mixtures by ^{1}H and ^{19}F NMR spectroscopy showed that they contained mixtures of **21-α**, **21-β** and unreacted progesterone enol acetate **17** as well as other fluorinated side-products. Progesterone **13** was not detected.

The use of >1.1 equivalents of F_2 and long reaction times gave yellow or orange oils (**Table 4** entries 3, 4). Conditions that yielded crystalline crude products were 1.1 equivalents with 5 mL min^{-1} flow rate for 27 min (entry 2) and 1.1 equivalents with 10 mL min^{-1} flow rate for 13 min (entry 6). The **21-α**:**21-β** selectivity of all direct fluorination reactions was 38:62. The conditions described by entry 3 yielded the highest amounts of fluorinated side products, which were evident in the NMR spectrum. The spectra for entries 2 and 4-6 displayed similar amounts of fluorinated side-products. Therefore, from these initial studies, the optimal conditions consist of a small excess of F_2 with high flow rate. Entry 7 was monitored using ReactIR and is discussed in the next section.

Table 4: Reaction conditions for the fluorination of progesterone enol acetate **17** using F_2 (10% in N_2) at 0 °C and the nature of the crude products as determined from visible inspection.

Entry	Solvent	Flow rate / mL min^{-1}	Equiv. of F_2	Reaction time / min	Crude product
1	Formic acid	20	1.4	50	Oil
2	MeCN	5	1.1	27	Crystals
3	MeCN	5	2.0	48	Oil
4	MeCN	5	1.5	36	Oil
5	MeCN	10	1.5	18	Oil, crystals
6	MeCN	10	1.1	13	Crystals
7	MeCN	10	1.1	50	Crystals

2.3.2.1 ReactIR study

An *in situ* ReactIR probe was used to monitor the fluorination of progesterone enol acetate **17** using F_2 in MeCN at 0 °C, using the conditions described by entry 7 in **Table 4**. During the initial 14 min of the reaction, the stirred solution containing progesterone enol acetate (0.84 g) in MeCN (95 mL) was purged with N_2 gas. The flow of F_2 gas was introduced at t = 14 min and stopped at t = 70 min. The mass of crude products obtained was 0.87 g. Upon evaporation of solvents, analysis of the crude product mixture by ^{1}H and ^{19}F NMR spectroscopy showed that the reaction reached 50% conversion.

The starting material was subtracted from the spectra shown in **Figure 11** to view only the formation of products. The two keto-carbonyl stretching frequencies of 6α-fluoroprogesterone and 6β-fluoroprogesterone both occur at 1680 cm^{-1} and 1702 cm^{-1} (see Sections 2.1.2-2.1.3).

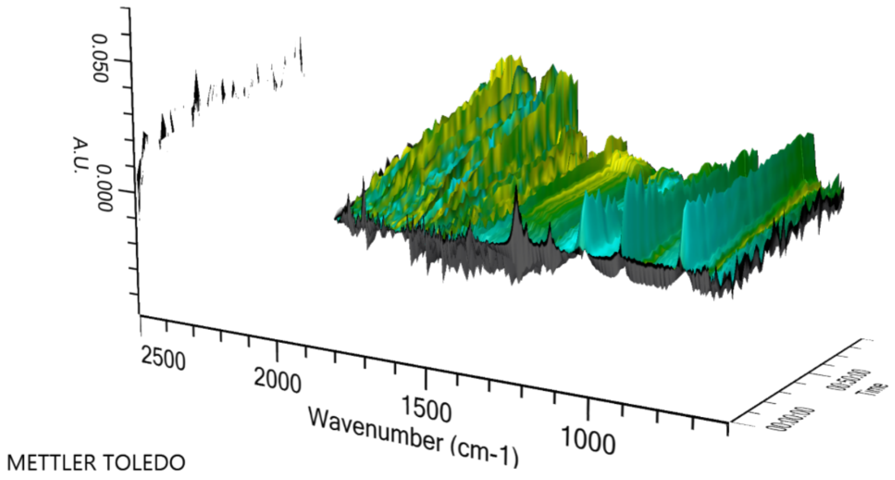

Figure 11: ReactIR study for the fluorination of progesterone enol acetate **17** using F_2 (10% in N_2). The formation of products is shown.

Selected peaks intensities were plotted versus time (**Figure 12**, **Figure 13**). The graph of peak intensities at 1702 cm^{-1} versus time was fitted to obtain k_{obs} = 6.19 × 10^{-4} s^{-1} (**Figure 13b**).

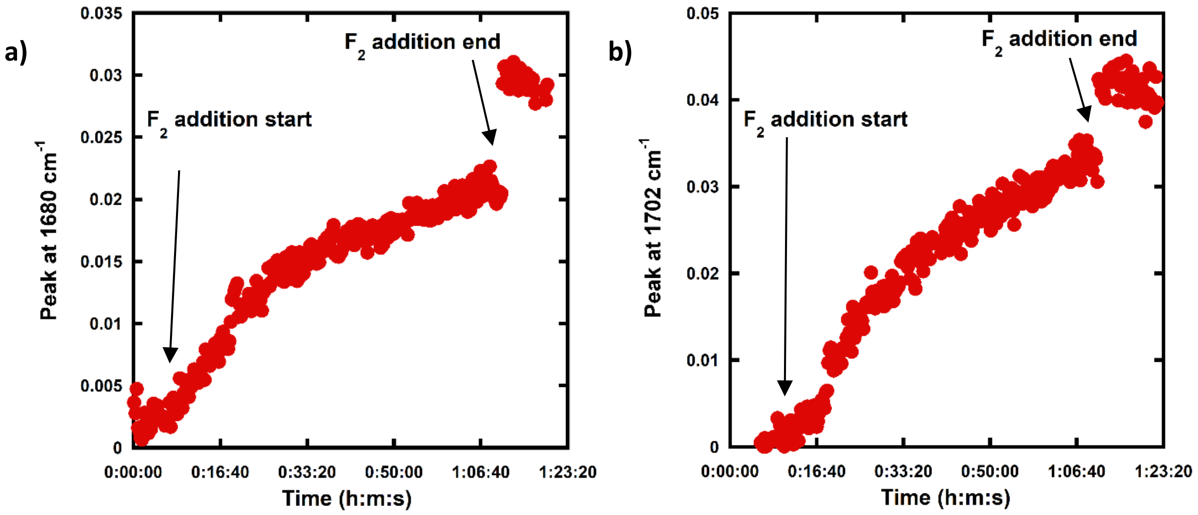

Figure 12: Peak intensities at (a) 1680 cm^{-1} and (b) 1702 cm^{-1} corresponding to the carbonyl bands of α/β-fluoroprogesterone products.

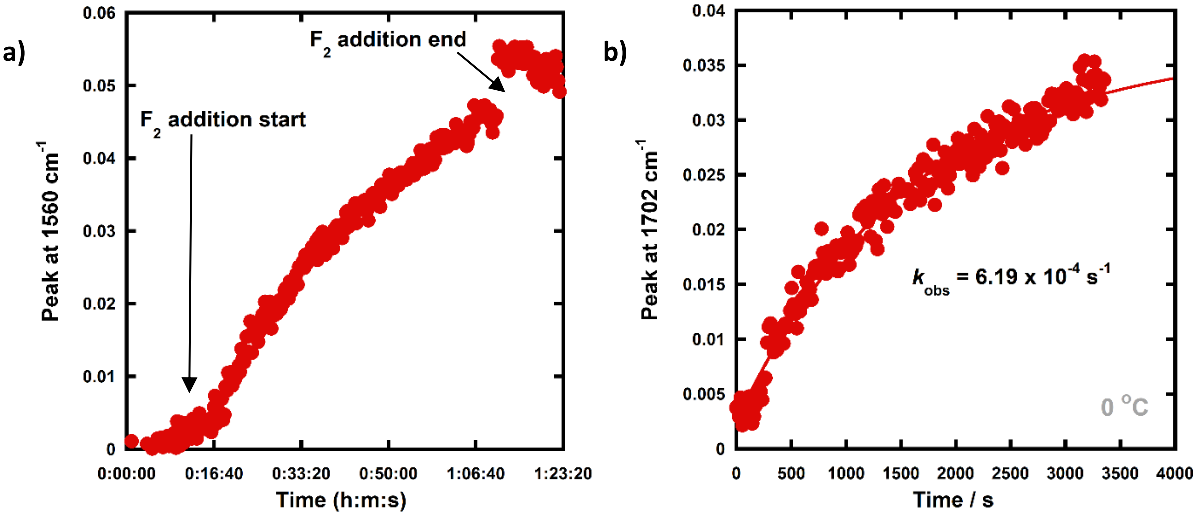

Figure 13: (a) An additional peak at 1560 cm^{-1}, possibly corresponding to a side product. (b) The peak at 1702 cm^{-1} fitted to a first-order exponential.

2.4 Kinetics studies on fluorination using N−F reagents

2.4.1 Method: NMR spectroscopy

The fluorination of progesterone enol acetate **17** by NFSI **8**, *N*-fluoropyridinium triflate **9** and 2,4,6-trimethyl-*N*-fluoropyridinium triflate **10** were studied by quantitative ^{1}H NMR spectroscopy. All kinetics experiments were carried out with excess N−F reagent in MeCN-d_3 at 25 °C to achieve pseudo-first order conditions (steroid **17** was not used in excess due to its relatively low solubility in MeCN-d_3 compared to the N−F reagents). In the case of NFSI, reactions were monitored discontinuously over the course of 1 day. The peaks at δ = 5.67 ppm and δ = 5.41 ppm correspond to C**6**H and C**4**H, respectively, of **17**. The doublet of triplets at δ = 5.05 ppm and the doublet at δ = 5.87 ppm are associated with C**6**H and C**4**H, respectively, of **21-β**. The doublet of doublet of doublets at δ = 5.20 ppm and the peak at δ = 5.92 ppm correspond to C**6**H and C**4**H, respectively, of **21-α**.

The signals corresponding to C**6**H of **17**, **21-α** and **21-β** were each integrated over the course of the reactions and the relative peak integrals gave exponential behaviours. The k_{obs} values were obtained from the fitting of plots of integral intensities versus time and second-order rate constants, k_2, were obtained from plots of k_{obs} against concentration of the N−F reagent.

2.4.2 Method: UV-vis spectrophotometry

Kinetics studies were carried out using a Varian Cary-100 Bio UV-vis Spectrophotometer equipped with a Cary Temperature Controller unit, or a Varian Cary-50 Bio UV-vis Spectrophotometer connected to a Varian Cary PCB-150 Water Peltier system. Samples were contained in quartz absorption cuvettes with a path length of 1 cm. All spectra were zeroed against air. Reactions involving Selectfluor™ **7** were followed by monitoring the disappearance of steroid enol esters **17-20** at a fixed wavelength corresponding to the maximum absorbance (λ_{max}) at 236 nm. Fluorination reactions involving Selectfluor™ **7** were carried out under pseudo-first-order conditions in the presence of excess Selectfluor™. Fluorinations involving N−F reagents **11a**, **11b** and **12** were conducted using excesses of enol esters **17-20**, by monitoring the disappearance of the N−F reagents at their λ_{max} values. Error values quoted in this section are standard error values obtained from data fitting in KaleidaGraph software. Stock solutions of steroid enol acetates **17-20** (5 mM) and fluorinating reagents: Selectfluor™ **7** (40 mM), **11a** and **11b** (5-10 mM), and **12** (5 mM) were prepared in

volumetric flasks in MeCN (HPLC grade). In a typical experiment, an aliquot of the stock solution of the steroid enol acetate was removed and diluted to the desired concentrations in a cuvette, which was placed in the spectrophotometer for 10 min to equilibrate to the required temperature. The required fluorinating reagent was then added, and kinetics studies were carried out.

Second-order rate constants for the reactions of progesterone enol acetate **17** with Selectfluor™ **7** and diCl-NFPy TfO⁻ **11a** at 4 different temperatures were determined as described above. The linear form of the Eyring equation was used to calculate activation parameters, where the slope of the linear plot of ln(k_2/T) vs 1/T is equal to −Δ$H^‡$/R. The entropy of activation, Δ$S^‡$, was calculated from the intercept of the linear plot, i.e. ln(k_B/h) + Δ$S^‡$/R. The values for Δ$G^‡$ were calculated from Equation 2.

$$ln\frac{k}{T} = \frac{-\Delta H^{\ddagger}}{RT} + \ln\left(\frac{k_B}{h}\right) + \frac{\Delta S^{\ddagger}}{R} \qquad (\mathbf{1})$$

$$\Delta G^{\ddagger} = \Delta H^{\ddagger} - T\Delta S^{\ddagger} \qquad (\mathbf{2})$$

2.4.3 Reference UV-vis spectra

Selectfluor™ is non-chromophoric, therefore the absorbance of enol acetates **17-20** at λ_{max} = 236 nm was monitored during the fluorination reactions. However, due to the presence of a broad absorbance between 200-240 nm in the UV-vis absorbance spectrum of diCl-NFPy TfO⁻ **11a**, the absorbance of steroid enol acetates **17-20** were masked. Therefore, the absorbance band of diCl-NFPy TfO⁻ **11a** at λ_{max} = 288 nm was monitored instead.

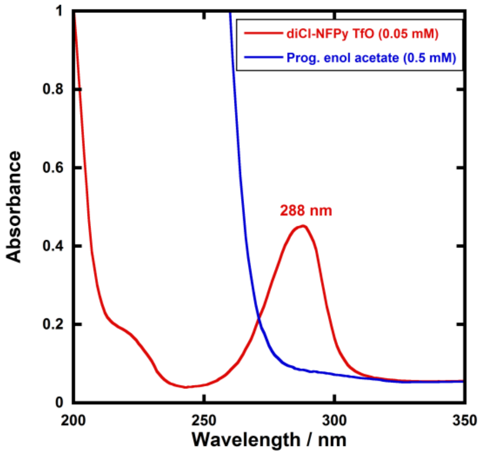

Figure 14: UV-vis spectrum for diCl-NFPy TfO⁻ **11a** (0.05 mM) and progesterone enol acetate **17** (0.50 mM).

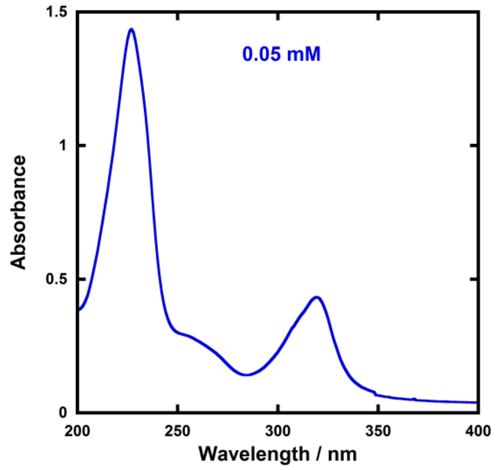

Figure 15: UV-vis spectrum for a solution of pentaCl-NFPy TfO^- **12** (0.05 mM).

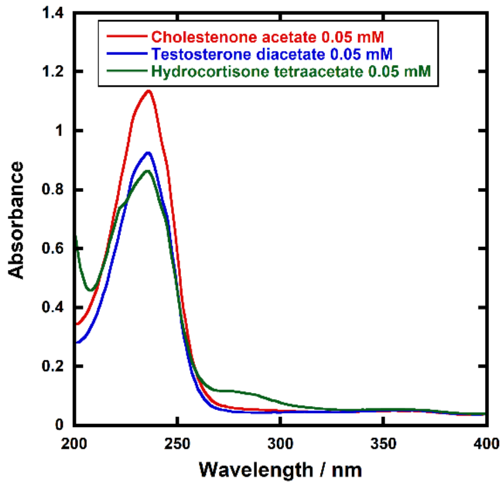

Figure 16: Reference UV-vis spectra for steroids **18-20** in MeCN. For all enol acetates, λ_{max} = 236 nm.

2.4.4 Extinction coefficients determination

Table 5: Extinction coefficients for progesterone enol acetate **17**, α-fluoroprogesterone **21-α** and β-fluoroprogesterone **21-β** in MeCN, determined using Beer-Lambert law and the figures below.

Steroid	λ_{max} / nm	ε / mol^{-1} dm^3 cm^{-1}
Progesterone enol acetate **17**	235	19466
α-Fluoroprogesterone **21-α**	233	16022
β-Fluoroprogesterone **21-β**	232	12764

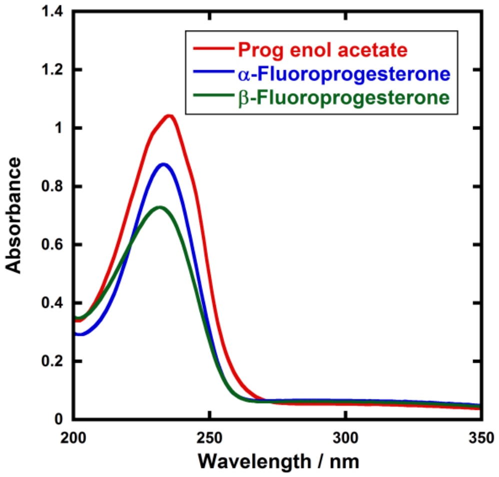

Figure 17: UV-vis spectra corresponding to progesterone enol acetate **17** (red line), **21-α** (blue line) and **21-β** (green line) in MeCN.

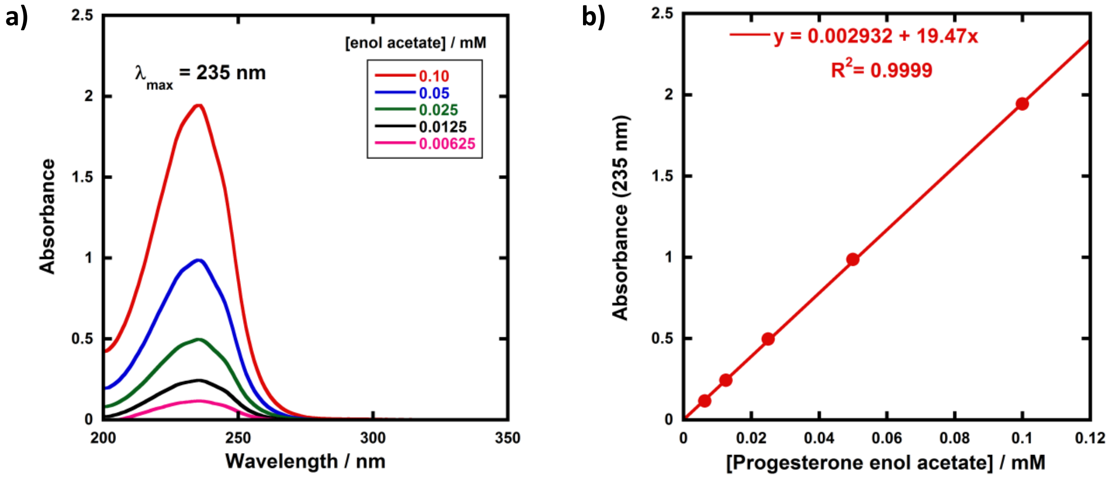

Figure 18: UV-vis spectra for progesterone enol acetate **17** at 5 concentrations.

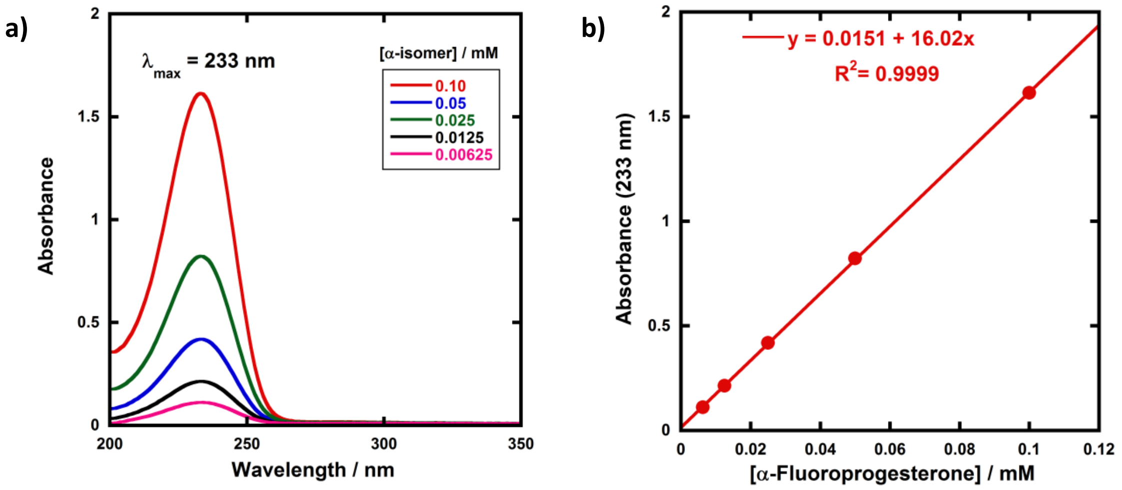

Figure 19: UV-vis spectra for α-fluoroprogesterone (**21-α**) at 5 concentrations.

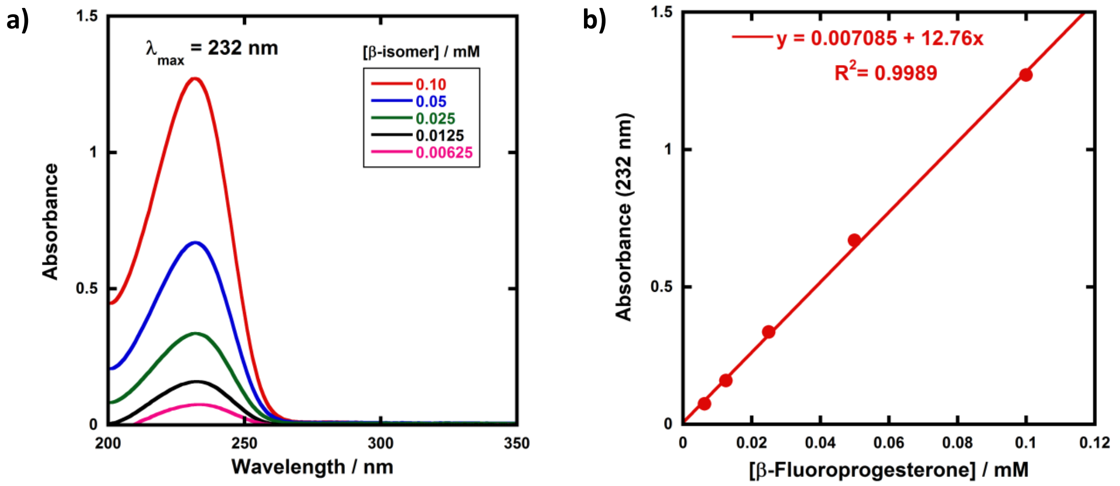

Figure 20: UV-vis spectra for β-fluoroprogesterone (**21-β**) at 5 concentrations.

2.4.5 Kinetics of fluorination of progesterone enol acetate 17 by Selectfluor™ 7

2.4.5.1 Kinetics studies at different temperatures and Eyring correlation

At 25 °C:

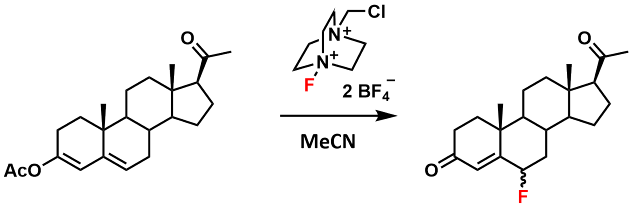

17 **21** α/β

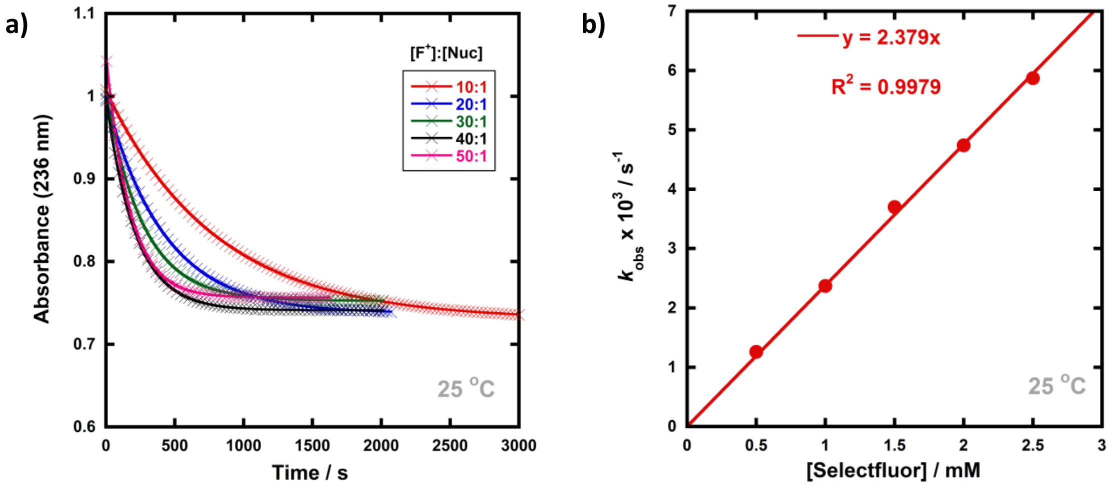

Figure 21: (a) Exponential decays of absorbance of progesterone enol acetate **17** with different concentrations of Selectfluor™ in MeCN at 25 °C. (b) Correlation of k_{obs} with [Selectfluor™].

Table 6: k_{obs} values at different concentrations of Selectfluor™ at 25 °C. Errors are standard error values.

Experiment	Ratio of Elec : Nuc	[Elec] : [Nuc] / mM	$k_{obs} \times 10^3$ / s^{-1}
**1**	10:1	0.50 : 0.05	1.260 ± 0.002
**2**	20:1	1.00 : 0.05	2.370 ± 0.004
**3**	30:1	1.50 : 0.05	3.700 ± 0.006
**4**	40:1	2.00 : 0.05	4.740 ± 0.008
**5**	50:1	2.50 : 0.05	5.87 ± 0.01

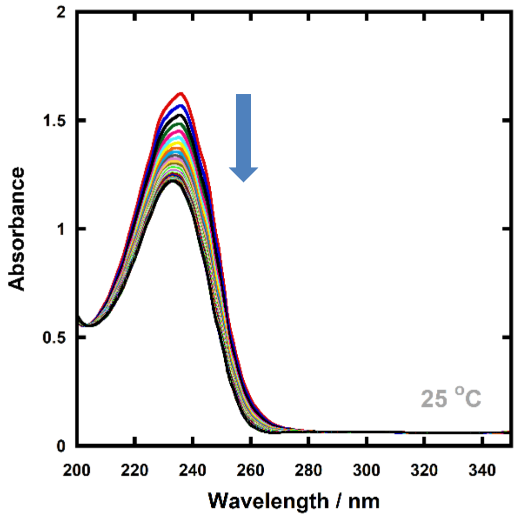

Figure 22: Time arrayed multi-wavelength analysis of fluorination of **17** using Selectfluor™ (30 equiv.).

At 30 °C:

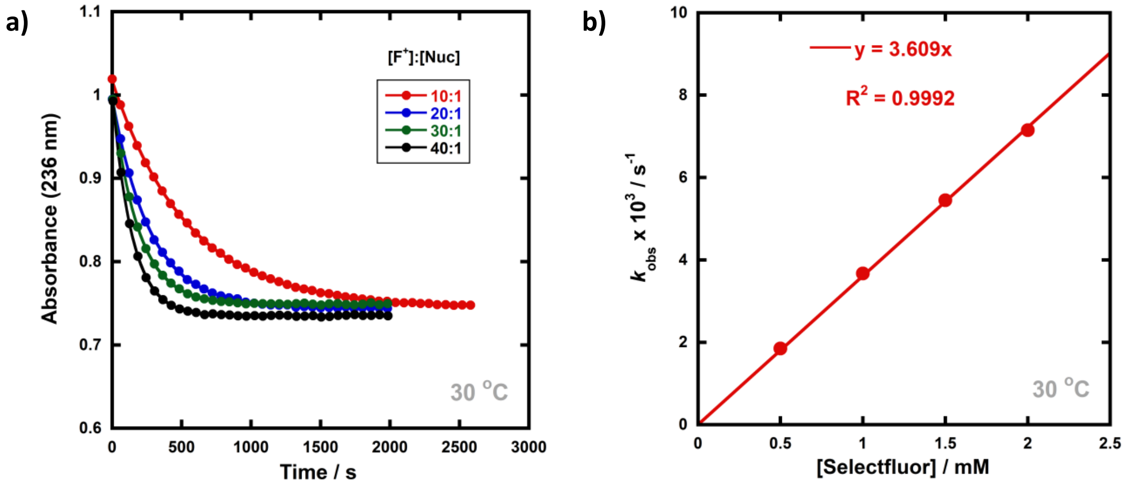

Figure 23: (a) Exponential decays of absorbance of progesterone enol acetate **17** with different concentrations of Selectfluor™ in MeCN at 30 °C. (b) Correlation of k_{obs} with [Selectfluor™].

Table 7: k_{obs} values at different concentrations of Selectfluor™ at 30 °C. Errors are standard error values.

Experiment	Ratio of Elec : Nuc	[Elec] : [Nuc] / mM	$k_{obs} \times 10^3$ / s^{-1}
**1**	10:1	0.50 : 0.05	1.850 ± 0.006
**2**	20:1	1.00 : 0.05	3.67 ± 0.02
**3**	30:1	1.50 : 0.05	5.45 ± 0.02
**4**	40:1	2.00 : 0.05	7.15 ± 0.03

At 35 °C:

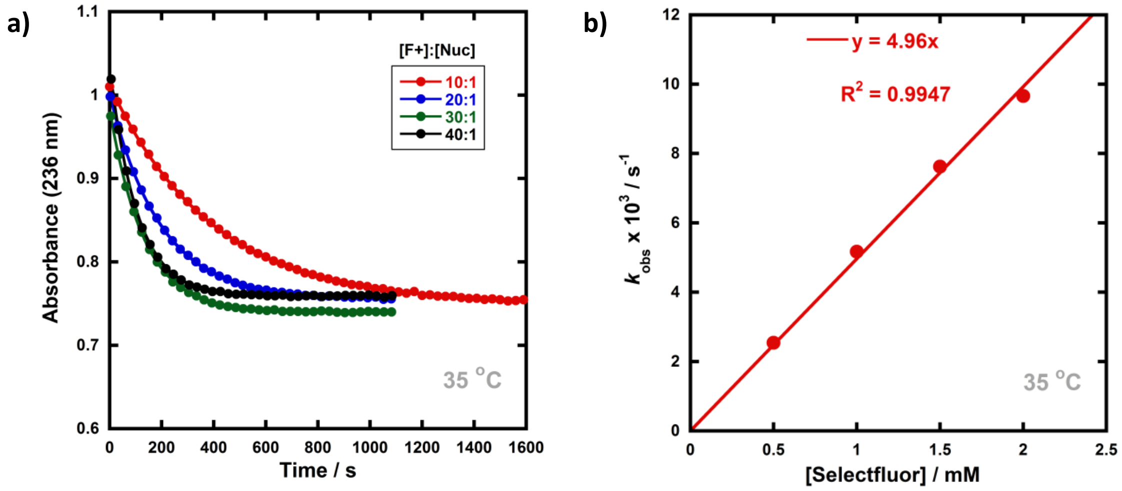

Figure 24: (a) Exponential decays of absorbance of progesterone enol acetate **17** with different concentrations of Selectfluor™ in MeCN at 35 °C. (b) Correlation of k_{obs} with [Selectfluor™].

Table 8: k_{obs} values at different concentrations of Selectfluor™ at 35 °C. Errors are standard error values.

Experiment	Ratio of Elec : Nuc	[Elec] : [Nuc] / mM	$k_{obs} \times 10^3$ / s^{-1}
**1**	10:1	0.50 : 0.05	2.545 ± 0.009
**2**	20:1	1.00 : 0.05	5.17 ± 0.02
**3**	30:1	1.50 : 0.05	7.63 ± 0.03
**4**	40:1	2.00 : 0.05	9.66 ± 0.04

At 40 °C:

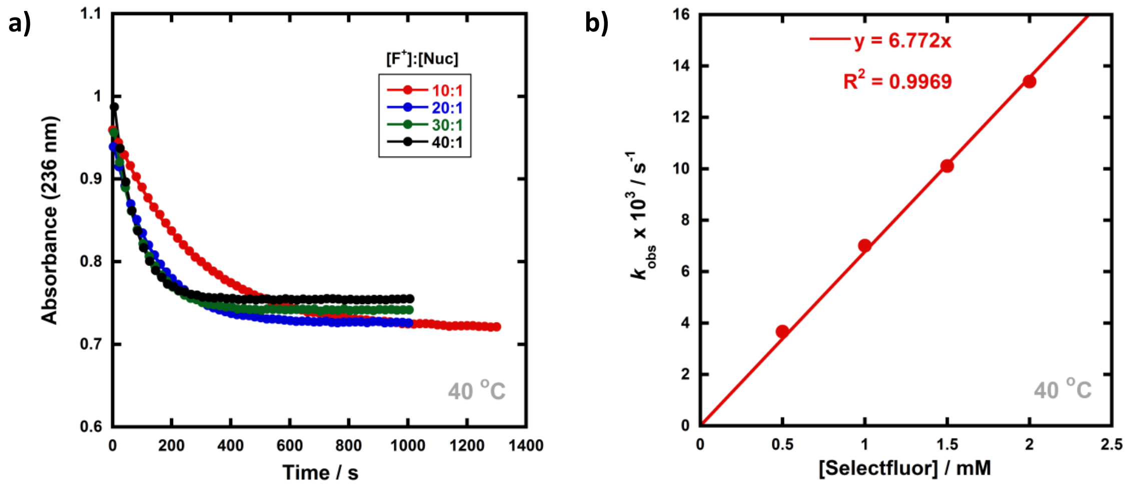

Figure 25: (a) Exponential decays of absorbance of progesterone enol acetate **17** with different concentrations of Selectfluor™ in MeCN at 40 °C. (b) Correlation of k_{obs} with [Selectfluor™].

Table 9: k_{obs} values at different concentrations of Selectfluor™ at 40 °C. Errors are standard error values.

**Experiment**	**Ratio of Elec : Nuc**	**[Elec] : [Nuc] / mM**	$k_{obs} \times 10^3$ / s^{-1}
**1**	10:1	0.50 : 0.05	3.67 ± 0.02
**2**	20:1	1.00 : 0.05	7.01 ± 0.04
**3**	30:1	1.50 : 0.05	10.11 ± 0.05
**4**	40:1	2.00 : 0.05	13.39 ± 0.07

Summary of rate constants:

Table 10: Summary of second-order rate constants, k_2, for fluorination of progesterone enol acetate **17** using Selectfluor™ **7** at 4 different temperatures.

N–F reagent	k_2 (25 °C) / M^{-1} s^{-1}	k_2 (30 °C) / M^{-1} s^{-1}	k_2 (35 °C) / M^{-1} s^{-1}	k_2 (40 °C) / M^{-1} s^{-1}
Selectfluor™	2.38	3.61	4.96	6.77

Eyring correlation:

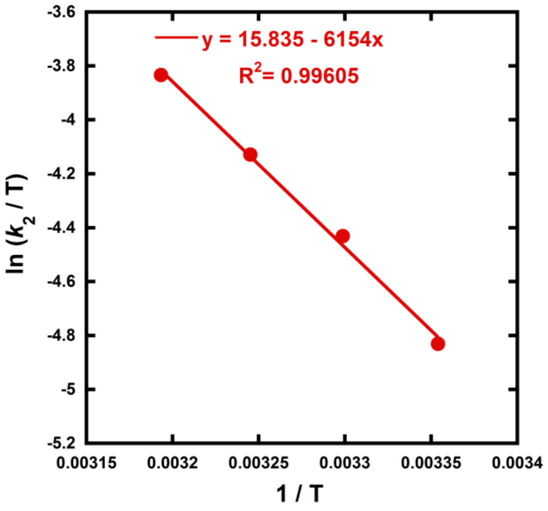

Figure 26: Eyring plot for fluorination of progesterone enol acetate **17** by Selectfluor™ **7** at 4 different temperatures.

Activation parameters: determined using Eyring correlation

$\Delta H^{\ddagger}$ = +51.2 kJ mol^{-1}

$\Delta S^{\ddagger}$ = −65.9 J K^{-1} mol^{-1}

$\Delta G^{\ddagger}$ = +70.8 kJ mol^{-1}

2.4.5.2 Kinetics studies with water

5% water:

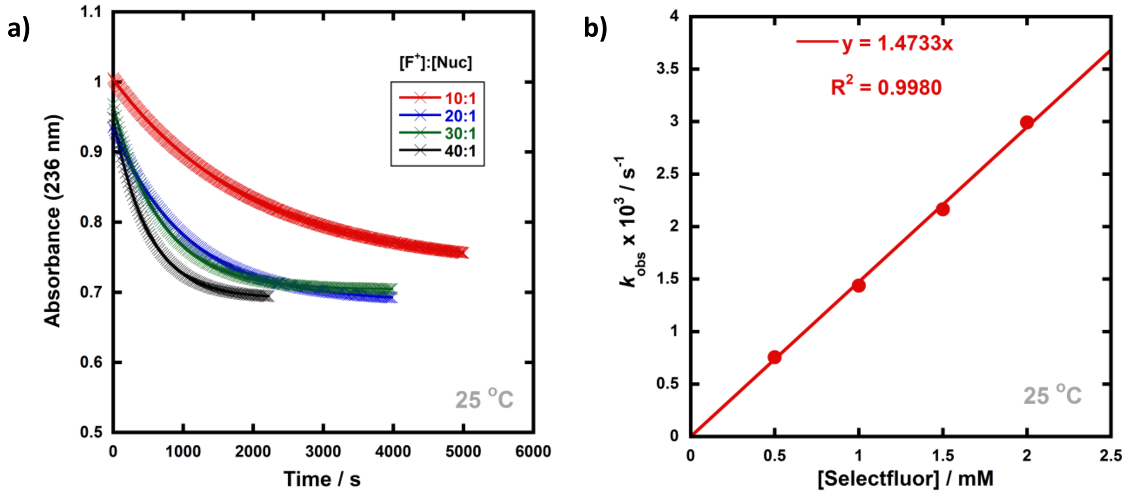

Figure 27: (a) Exponential decays of absorbance of progesterone enol acetate **17** with different concentrations of Selectfluor™ at 25 °C, with 5% water in MeCN. (b) Correlation of k_{obs} with [Selectfluor™].

Table 11: k_{obs} values at different concentrations of Selectfluor™ at 25 °C, with 5% water in MeCN. Errors are standard error values.

Experiment	Ratio of Elec : Nuc	[Elec] : [Nuc] / mM	$k_{obs} \times 10^3$ / s^{-1}
**1**	10:1	0.50 : 0.05	0.757 ± 0.004
**2**	20:1	1.00 : 0.05	1.439 ± 0.008
**3**	30:1	1.50 : 0.05	2.165 ± 0.008
**4**	40:1	2.00 : 0.05	2.99 ± 0.01

10% water:

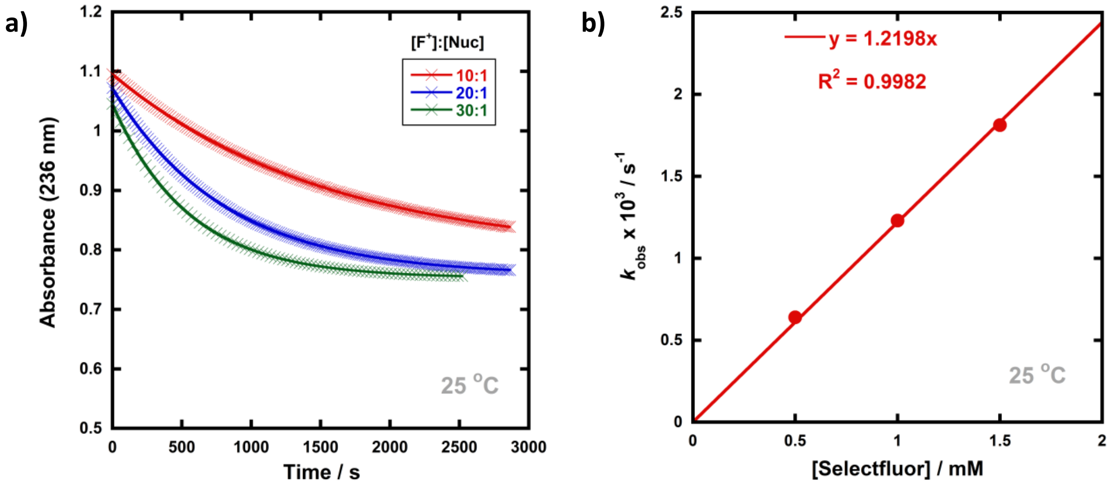

Figure 28: (a) Exponential decays of absorbance of progesterone enol acetate **17** with different concentrations of Selectfluor™ at 25 °C, with 10% water in MeCN. (b) Correlation of k_{obs} with [Selectfluor™].

Table 12: k_{obs} values at different concentrations of Selectfluor™ at 25 °C, with 10% water in MeCN. Errors are standard error values.

Experiment	Ratio of Elec : Nuc	[Elec] : [Nuc] / mM	$k_{obs} \times 10^3$ / s^{-1}
**1**	10:1	0.50 : 0.05	0.6396 ± 0.0008
**2**	20:1	1.00 : 0.05	1.230 ± 0.001
**3**	30:1	1.50 : 0.05	1.813 ± 0.002

20% water:

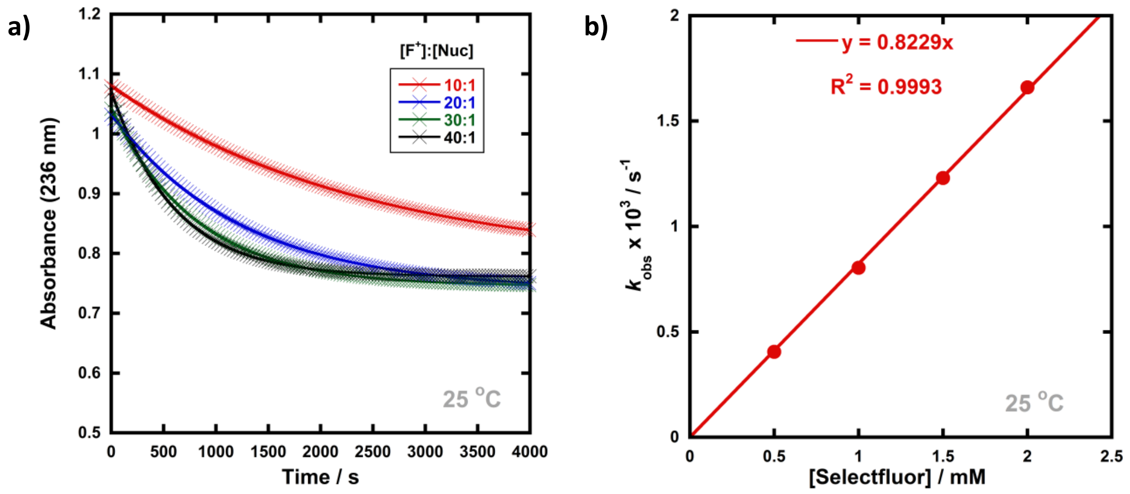

Figure 29: (a) Exponential decays of absorbance of progesterone enol acetate **17** with different concentrations of Selectfluor™ at 25 °C, with 20% water in MeCN. (b) Correlation of k_{obs} with [Selectfluor™].

Table 13: k_{obs} values at different concentrations of Selectfluor™ at 25 °C, with 20% water in MeCN. Errors are standard error values.

Experiment	Ratio of Elec : Nuc	[Elec] : [Nuc] / mM	$k_{obs} \times 10^3$ / s^{-1}
**1**	10:1	0.50 : 0.05	0.4060 ± 0.0008
**2**	20:1	1.00 : 0.05	0.8040 ± 0.0008
**3**	30:1	1.50 : 0.05	1.230 ± 0.002
**4**	40:1	2.00 : 0.05	1.660 ± 0.002

30% water:

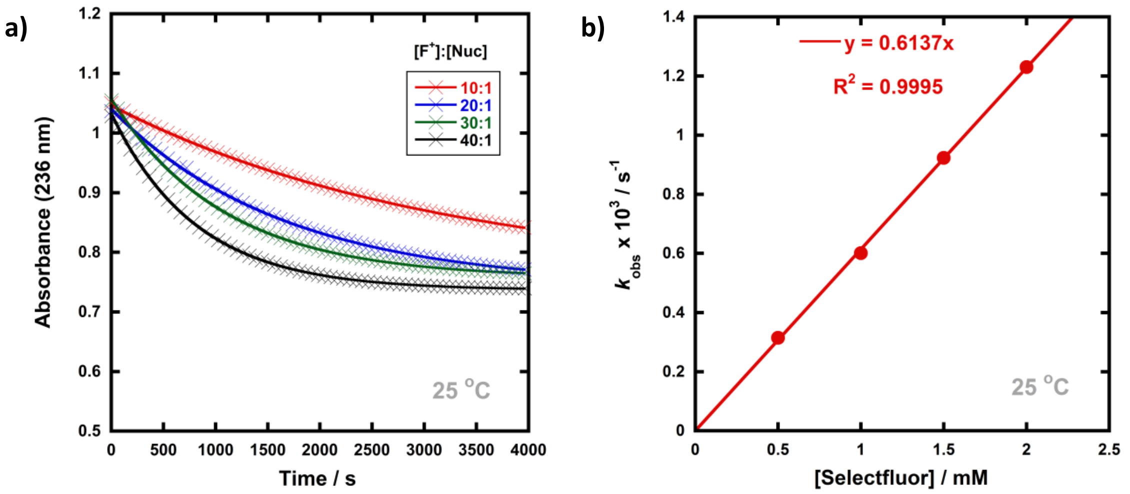

Figure 30: (a) Exponential decays of absorbance of progesterone enol acetate **17** with different concentrations of Selectfluor™ at 25 °C, with 30% water in MeCN. (b) Correlation of k_{obs} with [Selectfluor™].

Table 14: k_{obs} values at different concentrations of Selectfluor™ at 25 °C, with 30% water in MeCN. Errors are standard error values.

Experiment	Ratio of Elec : Nuc	[Elec] : [Nuc] / mM	$k_{obs} \times 10^3$ / s^{-1}
**1**	10:1	0.50 : 0.05	0.315 ± 0.003
**2**	20:1	1.00 : 0.05	0.601 ± 0.003
**3**	30:1	1.50 : 0.05	0.923 ± 0.001
**4**	40:1	2.00 : 0.05	1.230 ± 0.003

2.4.5.3 Kinetics studies with methanol

10% methanol:

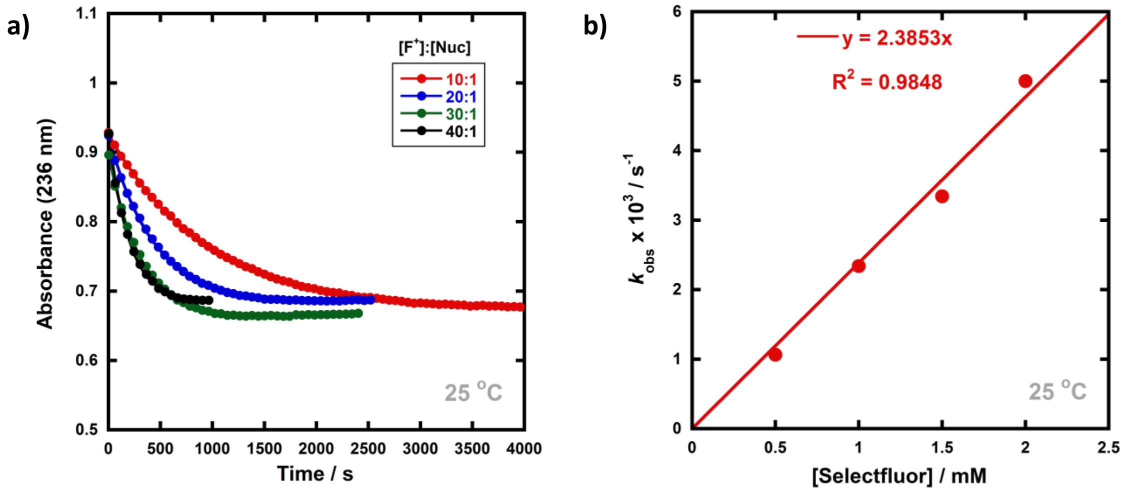

Figure 31: (a) Exponential decays of absorbance of progesterone enol acetate **17** with different concentrations of Selectfluor™ at 25 °C, with 10% MeOH in MeCN. (b) Correlation of k_{obs} with [Selectfluor™].

Table 15: k_{obs} values at different concentrations of Selectfluor™ at 25 °C, with 10% MeOH in MeCN. Errors are standard error values.

Experiment	Ratio of Elec : Nuc	[Elec] : [Nuc] / mM	$k_{obs} \times 10^3$ / s^{-1}
**1**	10:1	0.50 : 0.05	1.065 ± 0.004
**2**	20:1	1.00 : 0.05	2.34 ± 0.02
**3**	30:1	1.50 : 0.05	3.34 ± 0.05
**4**	40:1	2.00 : 0.05	5.05 ± 0.09

20% methanol:

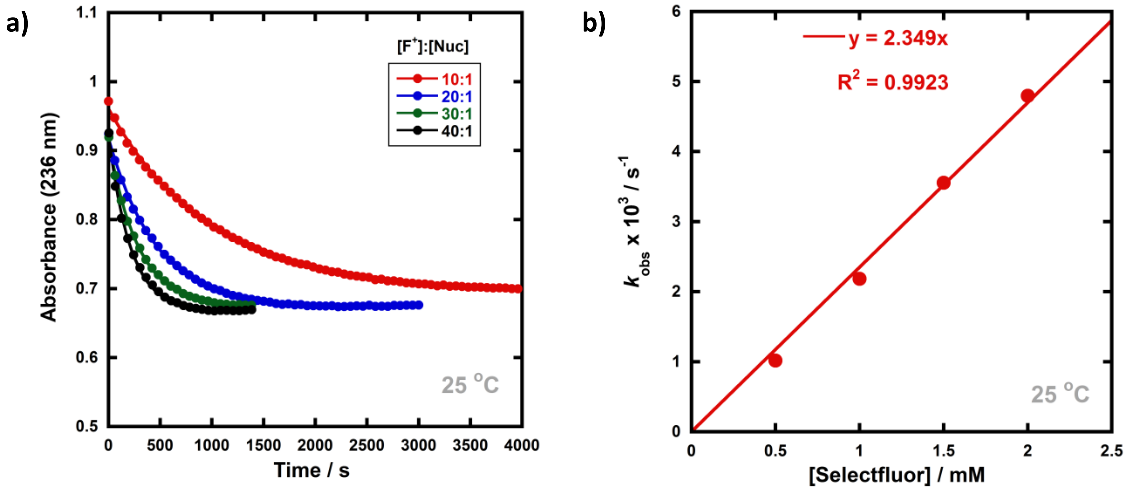

Figure 32: (a) Exponential decays of absorbance of progesterone enol acetate **17** with different concentrations of Selectfluor™ at 25 °C, with 20% MeOH in MeCN. (b) Correlation of k_{obs} with [Selectfluor™].

Table 16: k_{obs} values at different concentrations of Selectfluor™ at 25 °C, with 20% MeOH in MeCN. Errors are standard error values.

Experiment	Ratio of Elec : Nuc	[Elec] : [Nuc] / mM	$k_{obs} \times 10^3$ / s^{-1}
**1**	10:1	0.50 : 0.05	1.018 ± 0.007
**2**	20:1	1.00 : 0.05	2.18 ± 0.02
**3**	30:1	1.50 : 0.05	3.55 ± 0.06
**4**	40:1	2.00 : 0.05	4.80 ± 0.09

30% methanol:

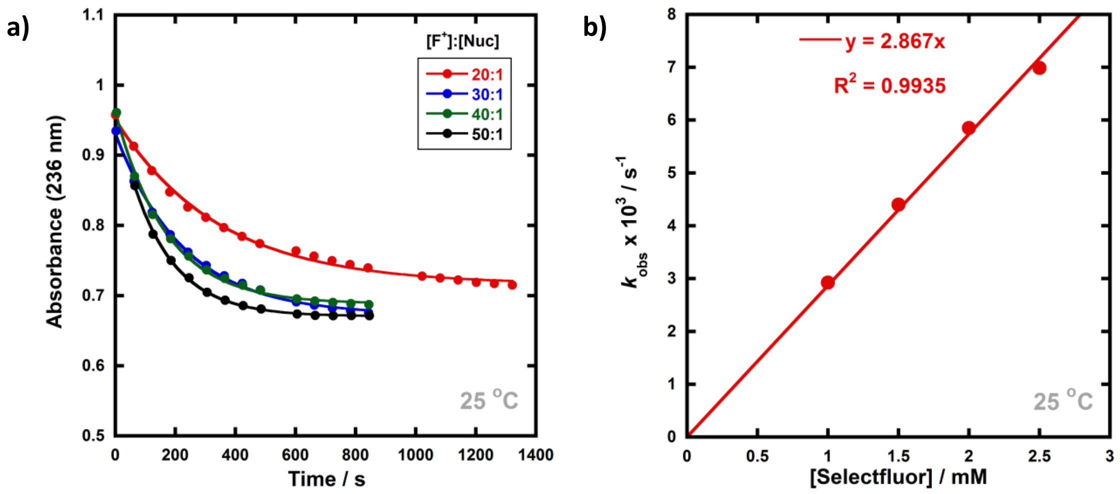

Figure 33: (a) Exponential decays of absorbance of progesterone enol acetate **17** with different concentrations of Selectfluor™ at 25 °C, with 30% MeOH in MeCN. (b) Correlation of k_{obs} with [Selectfluor™].

Table 17: k_{obs} values at different concentrations of Selectfluor™ at 25 °C, with 30% MeOH in MeCN. Errors are standard error values.

Experiment	Ratio of Elec : Nuc	[Elec] : [Nuc] / mM	$k_{obs} \times 10^3$ / s^{-1}
**1**	20:1	1.00 : 0.05	2.9 ± 0.1
**2**	30:1	1.50 : 0.05	4.4 ± 0.2
**3**	40:1	2.00 : 0.05	5.9 ± 0.2
**4**	50:1	2.50 : 0.05	7.0 ± 0.1

40% methanol:

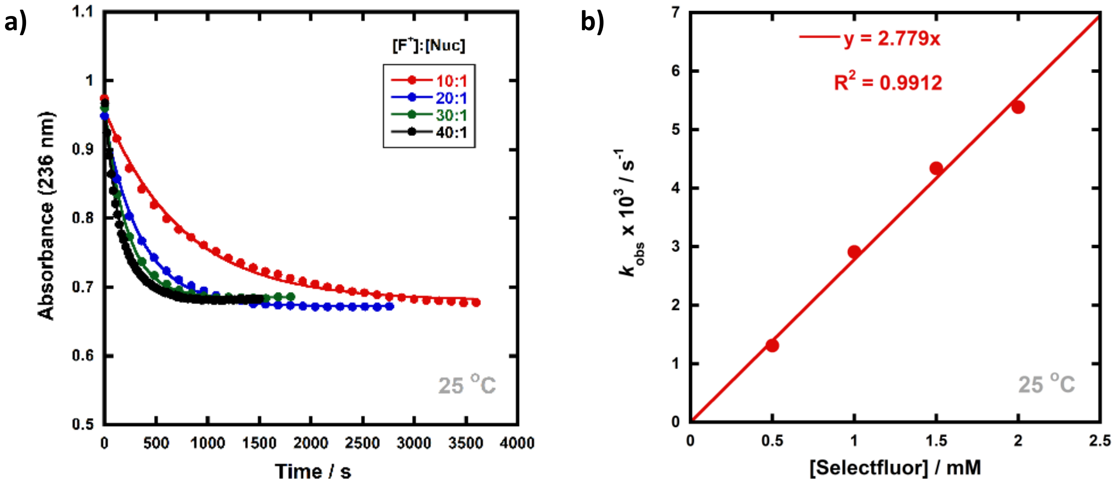

Figure 34: (a) Exponential decays of absorbance of progesterone enol acetate **17** with different concentrations of Selectfluor™ at 25 °C, with 40% MeOH in MeCN. (b) Correlation of k_{obs} with [Selectfluor™].

Table 18: k_{obs} values at different concentrations of Selectfluor™ at 25 °C, with 40% MeOH in MeCN. Errors are standard error values.

**Experiment**	**Ratio of Elec : Nuc**	**[Elec] : [Nuc] / mM**	**$k_{obs} \times 10^3$ / s^{-1}**
**1**	10:1	0.50 : 0.05	1.31 ± 0.05
**2**	20:1	1.00 : 0.05	2.85 ± 0.06
**3**	30:1	1.50 : 0.05	4.31 ± 0.09
**4**	40:1	2.00 : 0.05	5.44 ± 0.09

50% methanol:

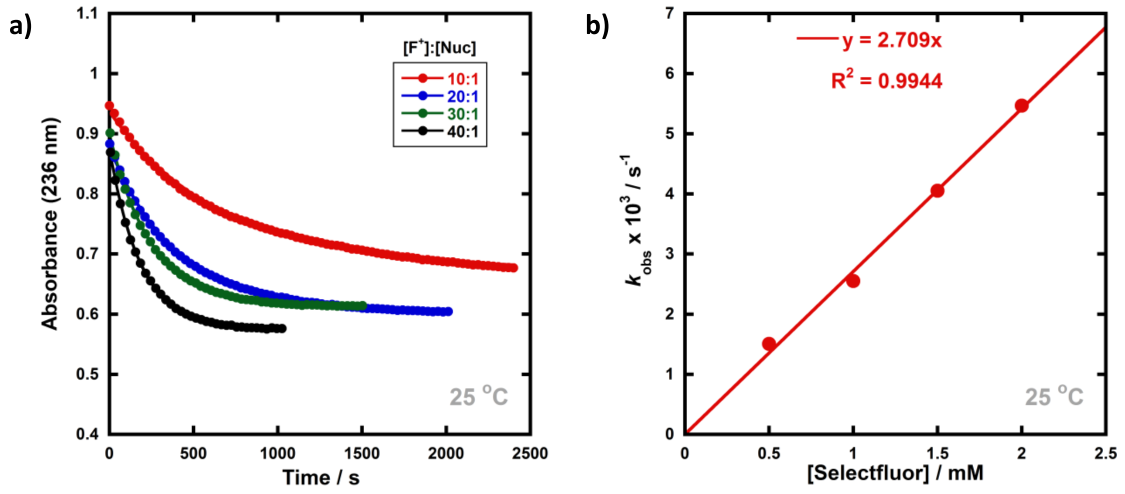

Figure 35: (a) Exponential decays of absorbance of progesterone enol acetate **17** with different concentrations of Selectfluor™ at 25 °C, with 50% MeOH in MeCN. (b) Correlation of k_{obs} with [Selectfluor™].

Table 19: k_{obs} values at different concentrations of Selectfluor™ at 25 °C, with 50% MeOH in MeCN. Errors are standard error values.

Experiment	Ratio of Elec : Nuc	[Elec] : [Nuc] / mM	$k_{obs} \times 10^3$ / s^{-1}
**1**	10:1	0.50 : 0.05	1.51 ± 0.02
**2**	20:1	1.00 : 0.05	2.55 ± 0.02
**3**	30:1	1.50 : 0.05	4.05 ± 0.03
**4**	40:1	2.00 : 0.05	5.47 ± 0.03

2.4.5.4 Solvent effects

Table 20: Rate constants (k_2) for the fluorination of progesterone enol acetate **17** by Selectfluor™ in the presence of additives (water and MeOH) in MeCN at 25 °C.

Additive	% Additive in MeCN (*v*/*v*)	k_2 / M^{-1} s^{-1}
None	-	2.38
Water	5	1.47
	10	1.22
	20	8.23×10^{-1}
	30	6.14×10^{-1}
MeOH	10	2.39
	20	2.35
	30	2.87
	40	2.78
	50	2.71

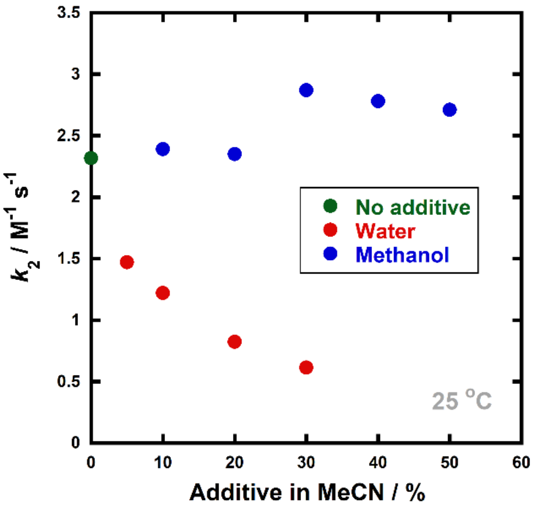

Figure 36: Effects of additives, methanol and water, upon the rate of fluorination of progesterone enol acetate.

2.4.6 Kinetics of fluorination of progesterone enol acetate 17 by diCl-NFPy TfO⁻ 11a at different temperatures and Eyring correlation

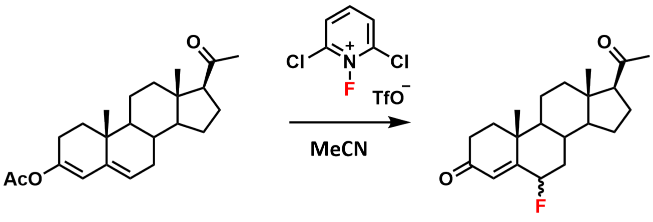

At 25 °C:

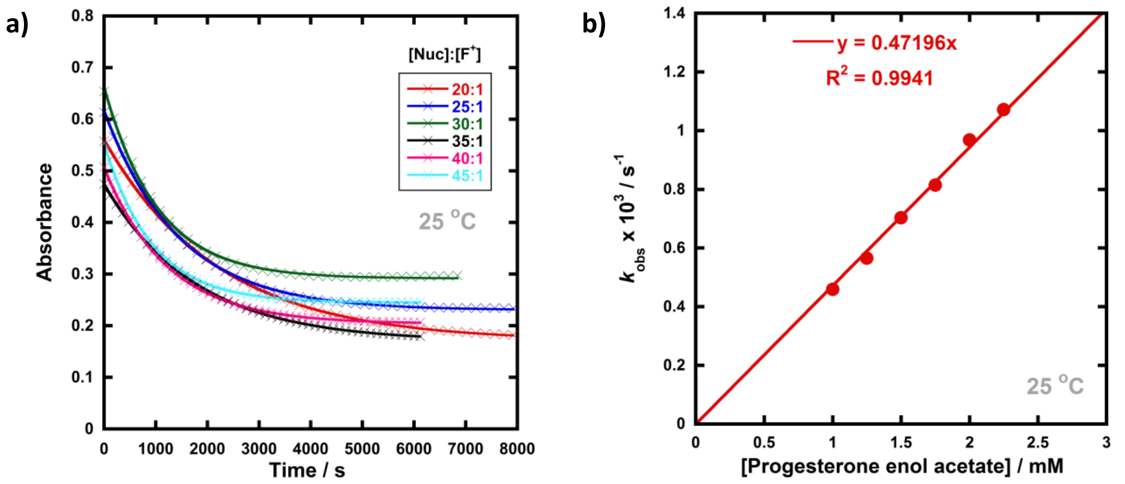

Figure 37: (a) Exponential decays of absorbance of diCl-NFPy TfO⁻ **11a** with different concentrations of progesterone enol acetate **17** in MeCN at 25 °C. (b) Correlation of k_{obs} with [progesterone enol acetate].

Table 21: k_{obs} values at different concentrations of **17** in MeCN at 25 °C. Errors are standard error values.

Experiment	Ratio of Nuc : Elec	[Nuc] : [Elec] / mM	$k_{obs} \times 10^3$ / s^{-1}
**1**	20:1	1.00 : 0.05	0.454 ± 0.002
**2**	25:1	1.25 : 0.05	0.566 ± 0.001
**3**	30:1	1.50 : 0.05	0.693 ± 0.004
**4**	35:1	1.75 : 0.05	0.815 ± 0.002
**5**	40:1	2.00 : 0.05	0.968 ± 0.009
**6**	45:1	2.25 : 0.05	1.072 ± 0.004

At 30 °C:

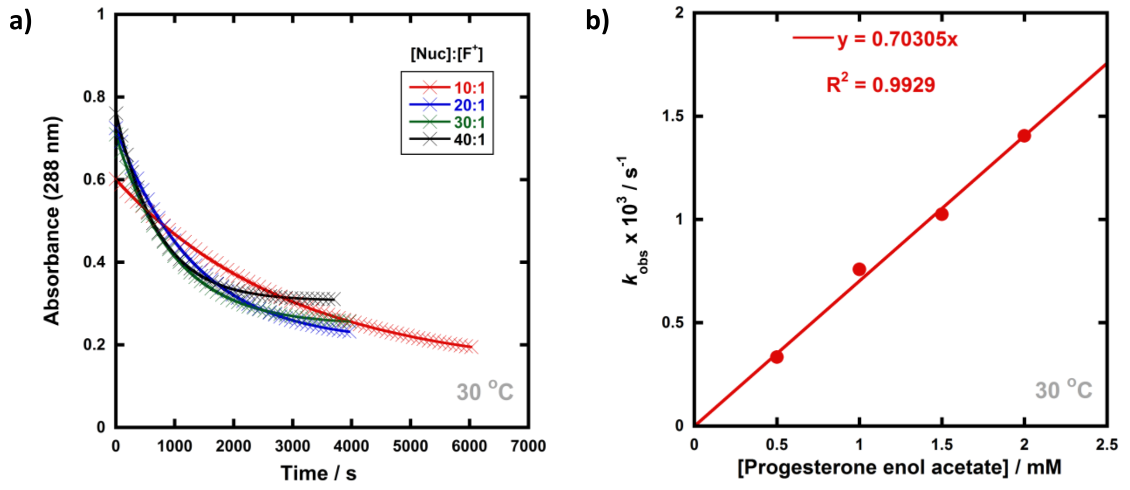

Figure 38: (a) Exponential decays of absorbance of diCl-NFPy TfO$^-$ **11a** with different concentrations of progesterone enol acetate **17** in MeCN at 30 °C. (b) Correlation of k_{obs} with [progesterone enol acetate].

Table 22: k_{obs} values at different concentrations of progesterone enol acetate **17** in MeCN at 30 °C. Errors are standard error values.

Experiment	Ratio of Nuc : Elec	[Nuc] : [Elec] / mM	$k_{obs} \times 10^3$ / s^{-1}
**1**	10:1	0.50 : 0.05	0.3334 ± 0.0007
**2**	20:1	1.00 : 0.05	0.759 ± 0.001
**3**	30:1	1.50 : 0.05	1.025 ± 0.003
**4**	40:1	2.00 : 0.05	1.405 ± 0.003

At 35 °C:

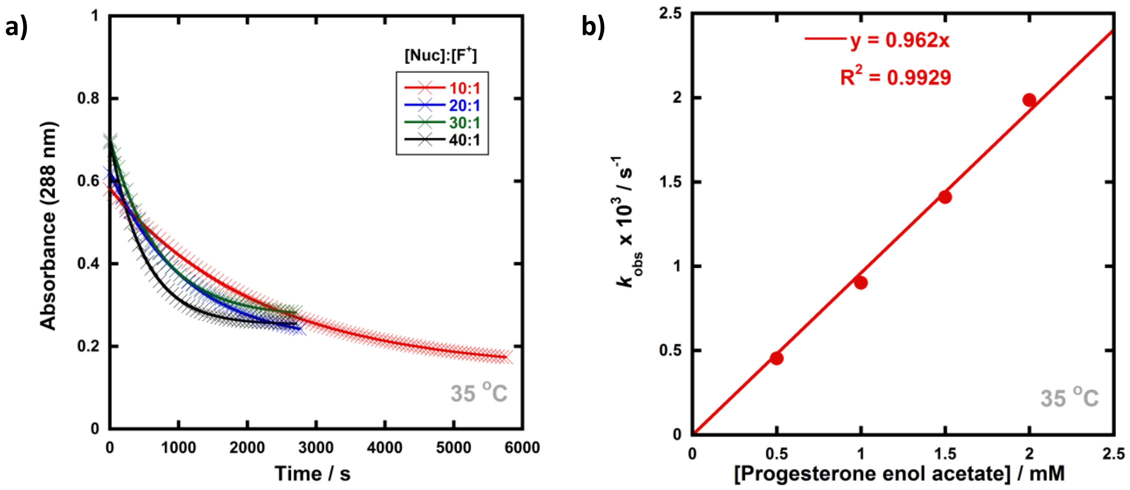

Figure 39: (a) Exponential decays of absorbance of diCl-NFPy TfO⁻ **11a** with different concentrations of progesterone enol acetate **17** in MeCN at 35 °C. (b) Correlation of k_{obs} with [progesterone enol acetate].

Table 23: k_{obs} values at different concentrations of progesterone enol acetate **17** in MeCN at 35 °C. Errors are standard error values.

Experiment	Ratio of Nuc : Elec	[Nuc] : [Elec] / mM	$k_{obs} \times 10^3$ / s^{-1}
**1**	10:1	0.50 : 0.05	0.4527 ± 0.0006
**2**	20:1	1.00 : 0.05	0.902 ± 0.002
**3**	30:1	1.50 : 0.05	1.410 ± 0.003
**4**	40:1	2.00 : 0.05	1.986 ± 0.006

At 40 °C:

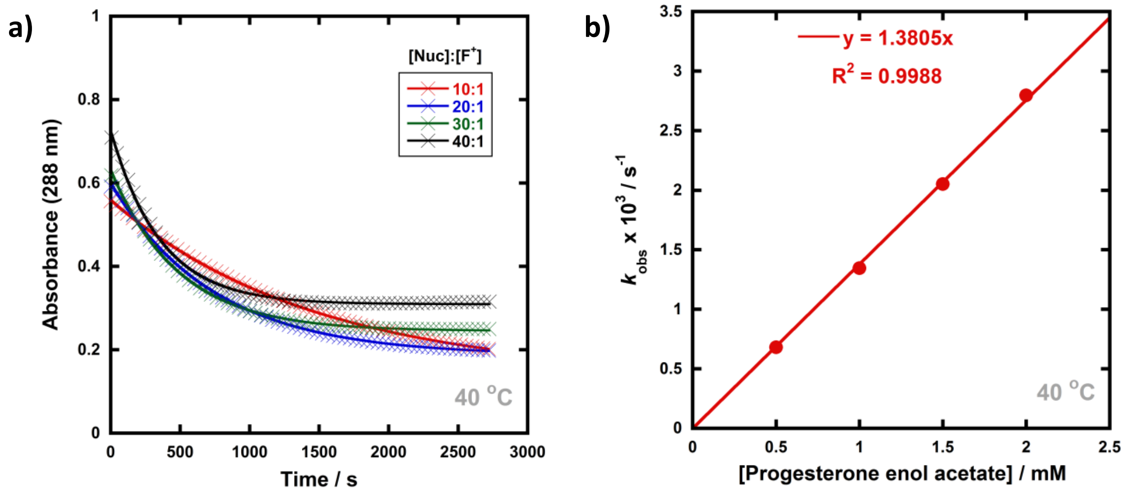

Figure 40: (a) Exponential decays of absorbance of diCl-NFPy TfO⁻ **11a** with different concentrations of **17** in MeCN at 40 °C. (b) Correlation of k_{obs} with [progesterone enol acetate].

Table 24: k_{obs} values at different concentrations of progesterone enol acetate **17** in MeCN at 40 °C. Errors are standard error values.

Experiment	Ratio of Nuc : Elec	[Nuc] : [Elec] / mM	$k_{obs} \times 10^3$ / s^{-1}
**1**	10:1	0.50 : 0.05	0.681 ± 0.002
**2**	20:1	1.00 : 0.05	1.346 ± 0.006
**3**	30:1	1.50 : 0.05	2.05 ± 0.01
**4**	40:1	2.00 : 0.05	2.80 ± 0.03

Summary of rate constants:

Table 25: Summary of second-order rate constants, k_2, for fluorination of progesterone enol acetate **17** using diCl-NFPy TfO⁻ **11a** at 4 different temperatures.

N–F reagent	k_2 (25 °C) / M^{-1} s^{-1}	k_2 (30 °C) / M^{-1} s^{-1}	k_2 (35 °C) / M^{-1} s^{-1}	k_2 (40 °C) / M^{-1} s^{-1}
diCl-NFPy TfO⁻ **11a**	4.72×10^{-1}	7.03×10^{-1}	9.62×10^{-1}	1.38

Eyring plot:

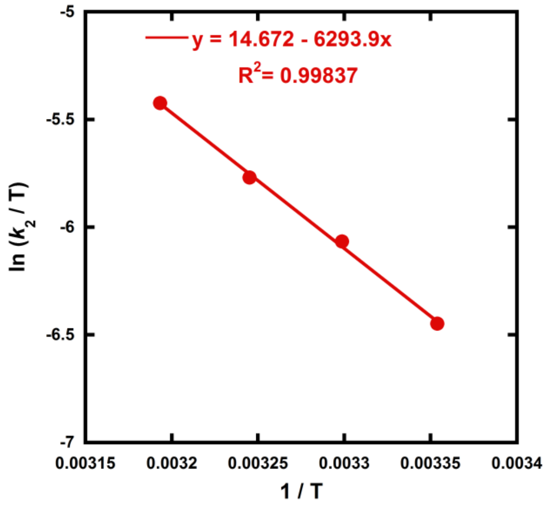

Figure 41: Eyring plot for fluorination of progesterone enol acetate **17** by diCl-NFPy TfO⁻ **11a** at 4 different temperatures.

Activation parameters: determined using Eyring correlation

$\Delta H^{\ddagger}$ = +52.3 kJ mol^{-1}

$\Delta S^{\ddagger}$ = −75.6 J K^{-1} mol^{-1}

$\Delta G^{\ddagger}$ = +74.8 kJ mol^{-1}

2.4.7 Kinetics of fluorination of progesterone enol acetate 17 by diCl NFPy BF_4^- 11b

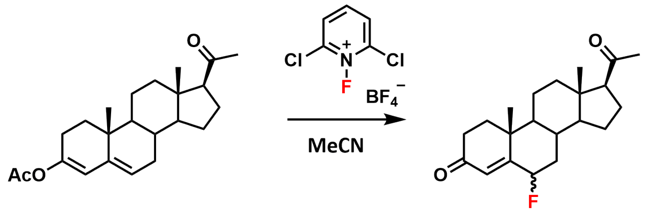

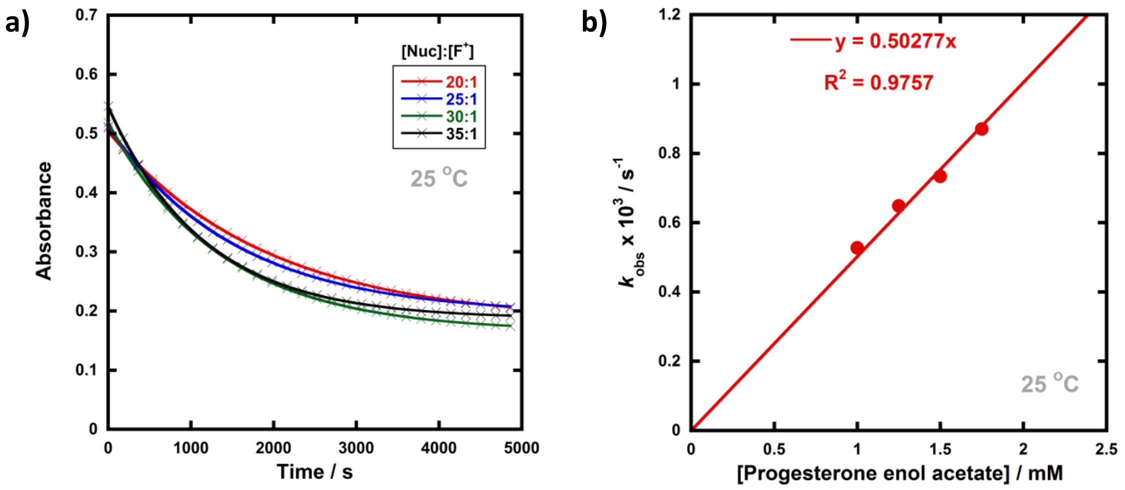

Figure 42: (a) Exponential decays of absorbance of diCl-NFPy BF_4^- **11b** with different concentrations of progesterone enol acetate **17** in MeCN at 25 °C. (b) Correlation of k_{obs} with [progesterone enol acetate].

Table 26: k_{obs} values at different concentrations of progesterone enol acetate **17** in MeCN at 25 °C. Errors are standard error values.

Experiment	Ratio of Nuc : Elec	[Nuc] : [Elec] / mM	$k_{obs} \times 10^3$ / s^{-1}
**1**	20:1	1.00 : 0.05	0.527 ± 0.006
**2**	25:1	1.25 : 0.05	0.648 ± 0.003
**3**	30:1	1.50 : 0.05	0.733 ± 0.002
**4**	35:1	1.75 : 0.05	0.870 ± 0.004

2.4.8 Kinetics of fluorination of progesterone enol acetate 17 by pentaCl NFPy TfO⁻ 12

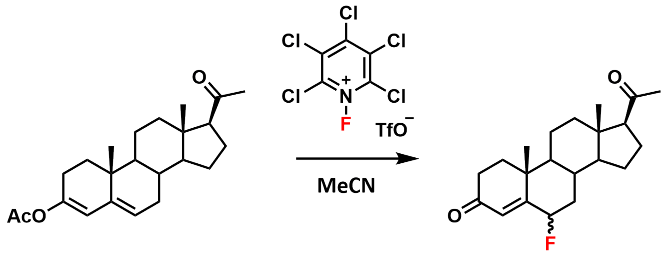

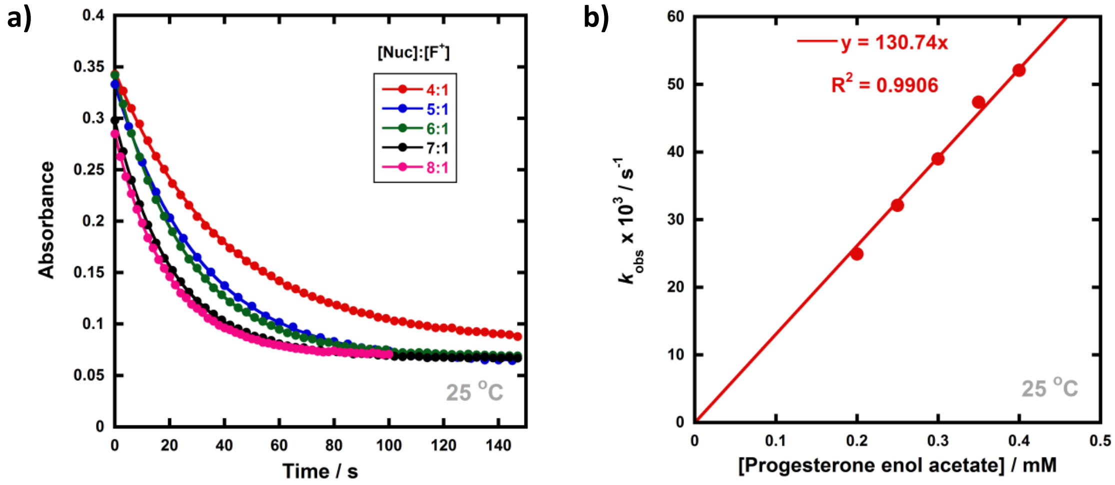

Figure 43: (a) Exponential decays of absorbance of pentaCl-NFPy TfO⁻ **12** with different concentrations of progesterone enol acetate **17** in MeCN at 25 °C. (b) Correlation of k_{obs} with [progesterone enol acetate].

Table 27: k_{obs} values at different concentrations of progesterone enol acetate **17** in MeCN at 25 °C. Errors are standard error values.

Experiment	Ratio of Nuc : Elec	[Nuc] : [Elec] / mM	k_{obs} / s^{-1}
**1**	4:1	0.20 : 0.05	0.0250 ± 0.0001
**2**	5:1	0.25 : 0.05	0.0321 ± 0.0001
**3**	6:1	0.30 : 0.05	0.0390 ± 0.0001
**4**	7:1	0.35 : 0.05	0.0474 ± 0.0002
**5**	8:1	0.40 : 0.05	0.0521 ± 0.0002

2.4.9 Kinetics of fluorination of progesterone enol acetate 17 by NFSI 8

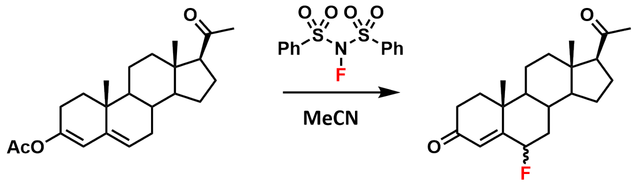

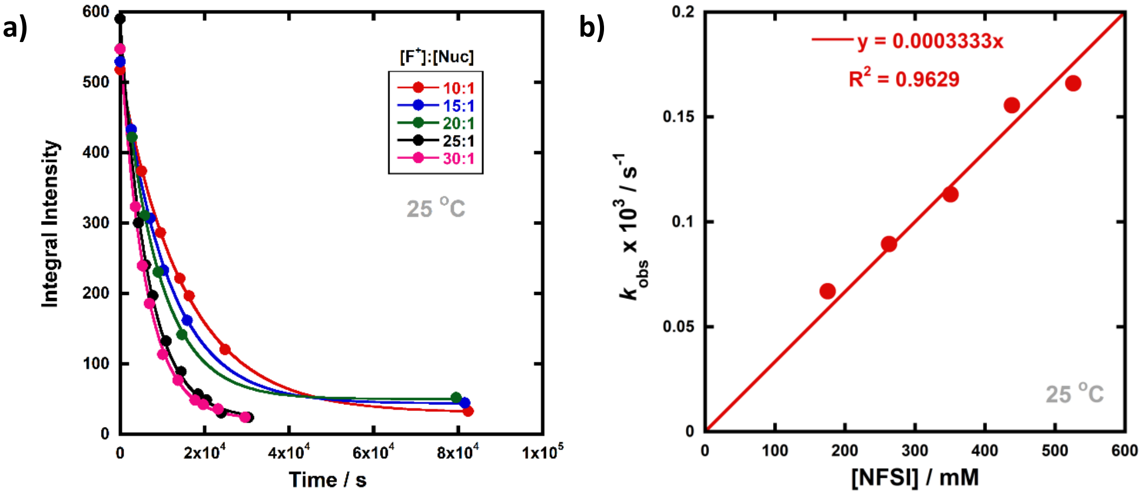

Figure 44: (a) Exponential decays of integral intensity of progesterone enol acetate **17** with different concentrations of NFSI **8** in MeCN-d_3 at 25 °C. (b) Correlation of k_{obs} with [NFSI].

Table 28: k_{obs} values at different concentrations of NFSI **8** in MeCN-d_3 at 25 °C. Errors are standard error values.

Experiment	Ratio of Elec : Nuc	[Elec] : [Nuc] / mM	$k_{obs} \times 10^3$ / s^{-1}
**1**	10:1	175.2 : 17.5	0.067 ± 0.001
**2**	15:1	262.8 : 17.5	0.089 ± 0.002
**3**	20:1	350.8 : 17.5	0.113 ± 0.003
**4**	25:1	438.4 : 17.5	0.156 ± 0.004
**5**	30:1	526.0 : 17.5	0.166 ± 0.004

The k_2 values for consumption of **17** (**Figure 44b**) and appearance of **21-β** (**Figure 45a**) and **21-α** (**Figure 45b**) gave similar values of 3.33 × 10^{-4} M^{-1} s^{-1}, 3.31 × 10^{-4} M^{-1} s^{-1} and 3.42 × 10^{-4} M^{-1} s^{-1}, respectively.

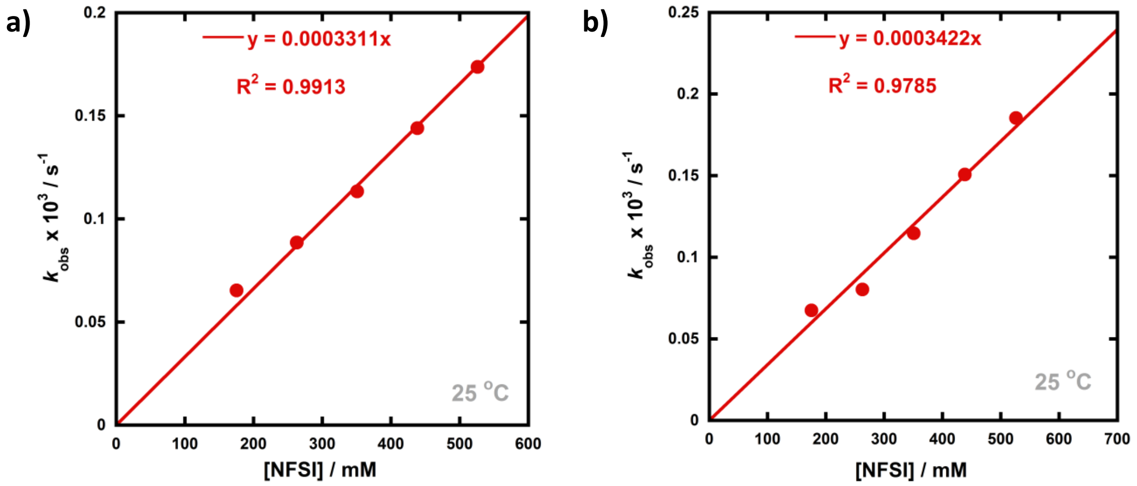

Figure 45: (a) β-isomer; (b) α-isomer.

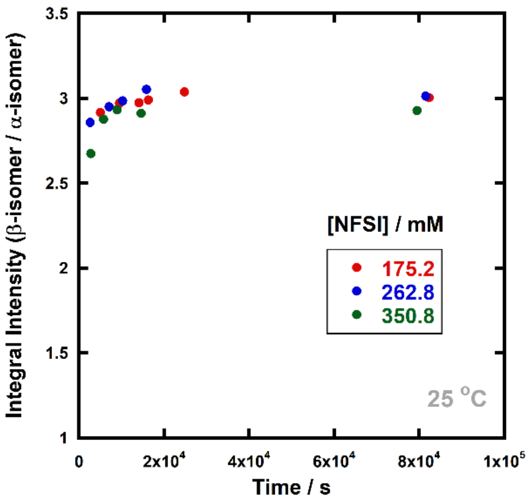

Figure 46: Ratios of β-fluoroprogesterone to α-fluoroprogesterone during the fluorination reactions of progesterone enol acetate **17** (17.5 mM) with NFSI (3 different concentrations) in MeCN-d_3, determined by integrating peaks corresponding to C4H of each isomer at 5.86 ppm and 5.91 ppm.

2.4.10 Kinetics of fluorination of progesterone enol acetate 17 by NFPy TfO⁻ 9

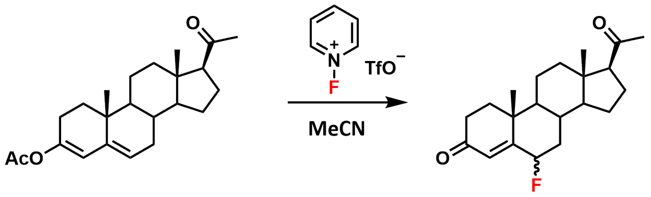

17 **21** α/β

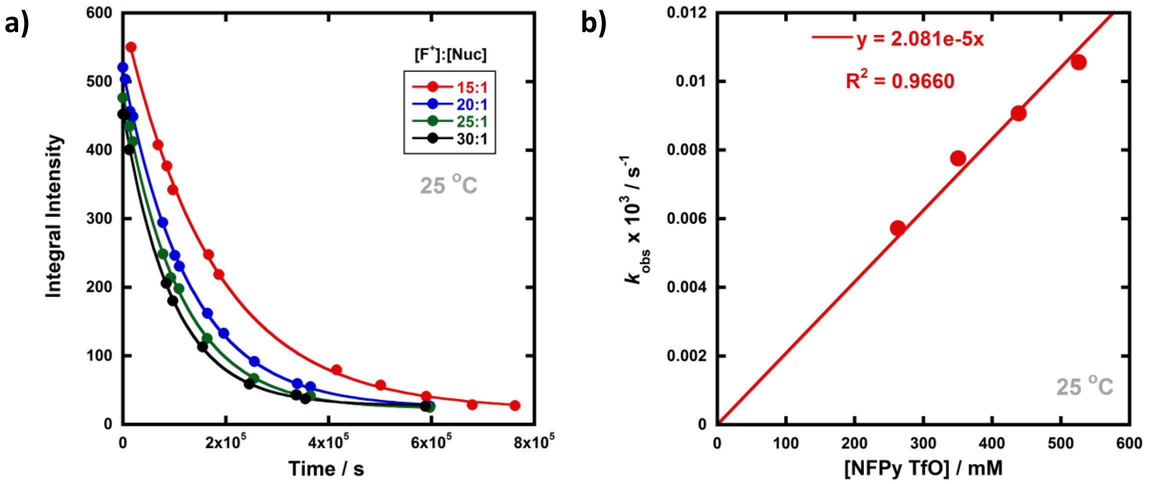

Figure 47: (a) Exponential decays of integral intensity of progesterone enol acetate **17** with different concentrations of NFPy TfO⁻ **9** in MeCN-d_3 at 25 °C. (b) Correlation of k_{obs} with [NFPy TfO⁻ **9**].

Table 29: k_{obs} values at different concentrations of NFPy TfO⁻ **9** in MeCN-d_3 at 25 °C. Errors are standard error values.

Experiment	Ratio of Elec : Nuc	[Elec] : [Nuc] / mM	$k_{obs} \times 10^3$ / s^{-1}
**1**	15:1	263.0 : 17.5	0.0057 ± 0.0001
**2**	20:1	350.5 : 17.5	0.0078 ± 0.0001
**3**	25:1	438.5 : 17.5	0.0091 ± 0.0001
**4**	30:1	526.0 : 17.5	0.0106 ± 0.0001

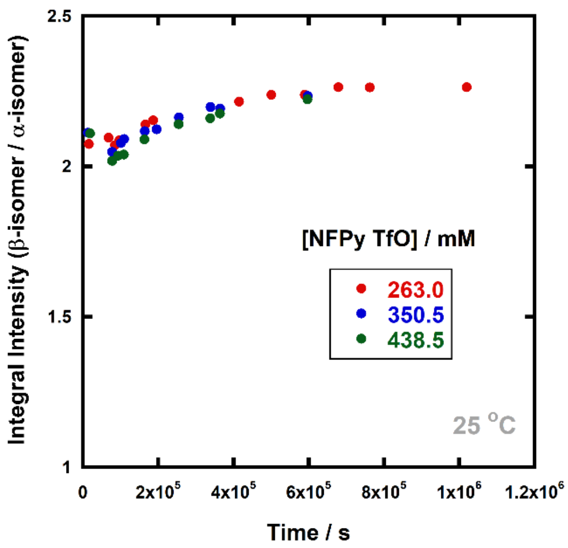

Figure 48: Ratios of β-fluoroprogesterone to α-fluoroprogesterone during the fluorination reactions of progesterone enol acetate **17** (17.5 mM) with NFPy TfO$^-$ (3 different concentrations) in MeCN-d_3, determined by integrating peaks corresponding to C4H of each isomer at 5.86 ppm and 5.90 ppm.

2.4.11 Kinetics of fluorination of progesterone enol acetate 17 by triMe NFPy TfO⁻ 10

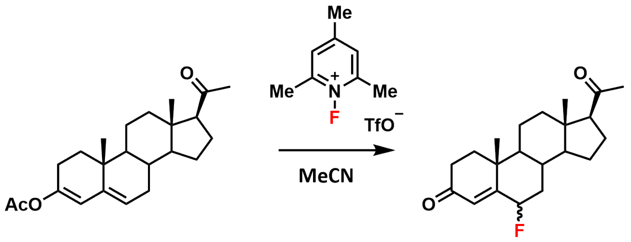

17 **21** α/β

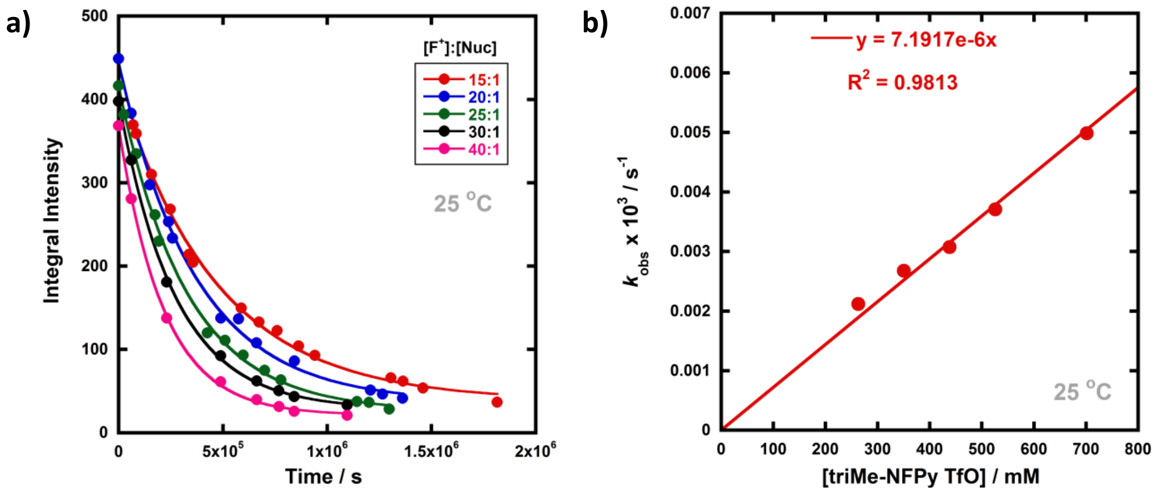

Figure 49: (a) Exponential decays of integral intensity of progesterone enol acetate **17** with different concentrations of triMe-NFPy TfO⁻ **10** in MeCN-d_3 at 25 °C. (b) Correlation of k_{obs} with [triMe-NFPy TfO⁻].

Table 30: k_{obs} values at different concentrations of triMe-NFPy TfO⁻ **10** in MeCN-d_3 at 25 °C. Errors are standard error values.

Experiment	Ratio of Elec : Nuc	[Elec] : [Nuc] / mM	$k_{obs} \times 10^3$ / s^{-1}
**1**	15:1	263.2 : 17.5	0.0021 ± 0.0001
**2**	20:1	350.5 : 17.5	0.0027 ± 0.0001
**3**	25:1	438.2 : 17.5	0.0031 ± 0.0001
**4**	30:1	525.9 : 17.5	0.0037 ± 0.0001
**5**	40:1	701.4 : 17.5	0.0046 ± 0.0001

2.4.12 Kinetics of fluorination of testosterone enol diacetate 18 by Selectfluor™ 7

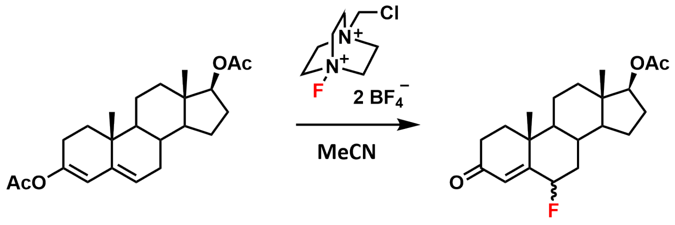

18 22 α/β

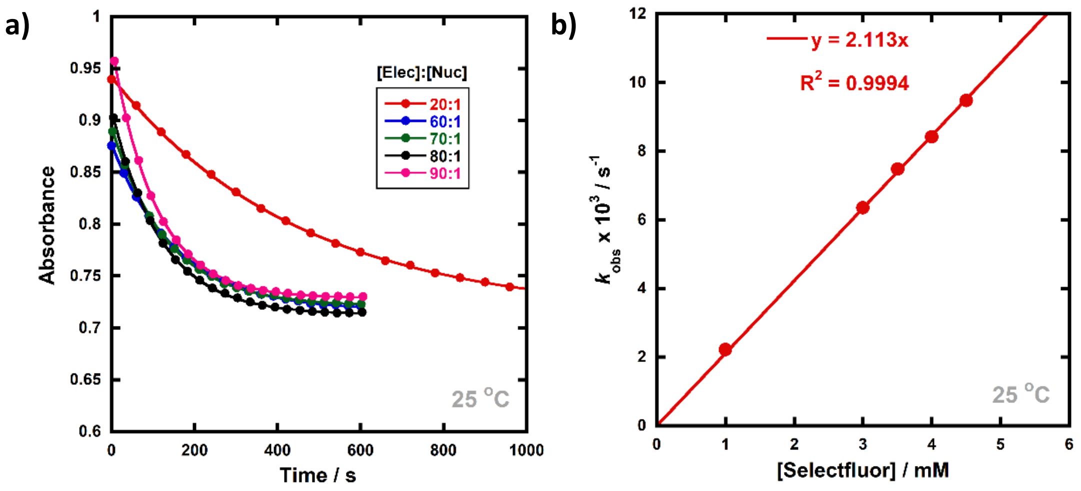

Figure 50: (a) Exponential decays of absorbance of testosterone enol diacetate **18** with different concentrations of Selectfluor™ in MeCN at 25 °C. (b) Correlation of k_{obs} with [Selectfluor™].

Table 31: k_{obs} values at different concentrations of Selectfluor™ at 25 °C. Errors are standard error values.

Experiment	Ratio of Elec : Nuc	[Elec] : [Nuc] / mM	$k_{obs} \times 10^3$ / s^{-1}
**1**	20:1	1.00 : 0.05	2.22 ± 0.01
**2**	60:1	3.00 : 0.05	6.35 ± 0.05
**3**	70.2:1	3.51 : 0.05	7.48 ± 0.04
**4**	80:1	4.00 : 0.05	8.41 ± 0.06
**5**	90.2:1	4.51 : 0.05	9.47 ± 0.05

2.4.13 Kinetics of fluorination of testosterone enol diacetate 18 by diCl-NFPy TfO⁻ 11a

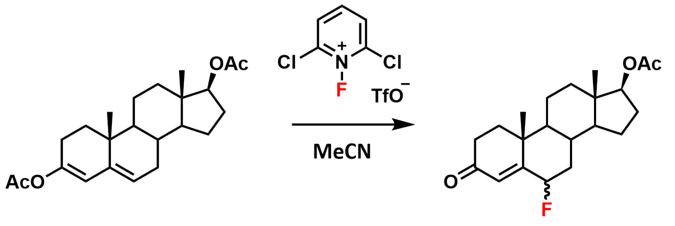

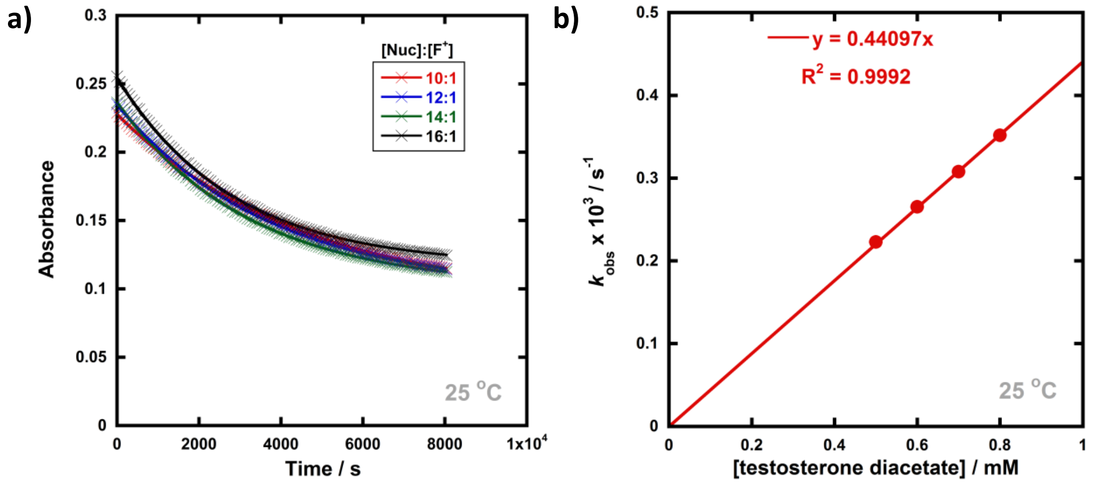

Figure 51: (a) Exponential decays of absorbance of diCl-NFPy TfO⁻ **11a** with different concentrations of testosterone enol diacetate **18** in MeCN at 25 °C. (b) Correlation of k_{obs} with [testosterone enol diacetate **18**].

Table 32: k_{obs} values at different concentrations of testosterone enol diacetate **18** at 25 °C. Errors are standard error values.

Experiment	Ratio of Nuc : Elec	[Nuc] : [Elec] / mM	$k_{obs} \times 10^3$ / s^{-1}
**1**	10:1	0.50 : 0.05	0.223 ± 0.001
**2**	12:1	0.60 : 0.05	0.265 ± 0.001
**3**	14:1	0.70 : 0.05	0.308 ± 0.001
**4**	16:1	0.80 : 0.05	0.352 ± 0.001

2.4.14 Kinetics of fluorination of testosterone enol diacetate 18 using pentaCl-NFPy TfO^- 12

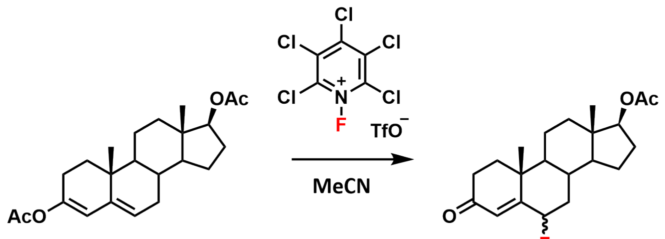

18 **22** α/β

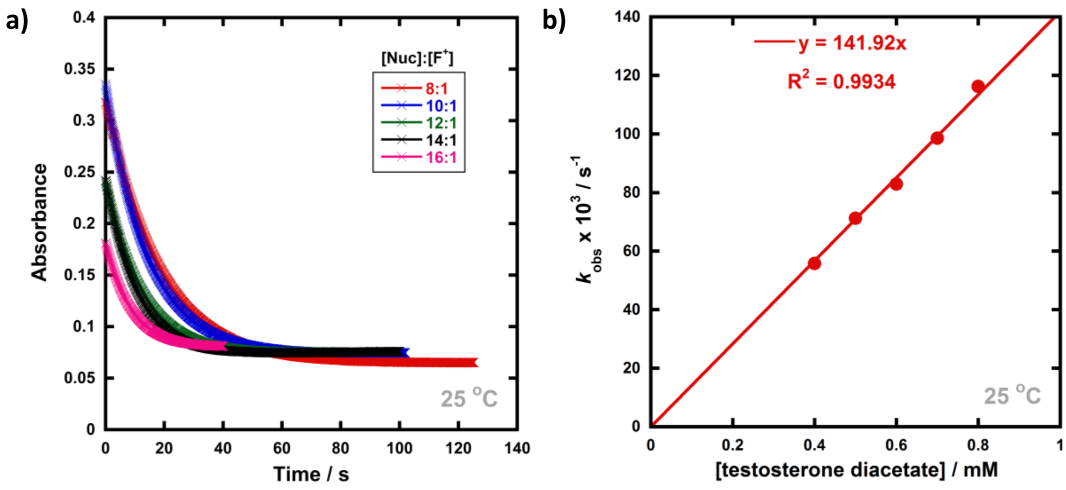

Figure 52: (a) Exponential decays of absorbance of pentaCl-NFPy TfO^- **12** with different concentrations of testosterone enol diacetate **18** in MeCN at 25 °C. (b) Correlation of k_{obs} with [testosterone enol diacetate].

Table 33: k_{obs} values at different concentrations of testosterone enol diacetate **18** in MeCN at 25 °C. Errors are standard error values.

**Experiment**	**Ratio of Nuc : Elec**	**[Nuc] : [Elec] / mM**	$k_{obs} \times 10^3$ / s^{-1}
**1**	8:1	0.40 : 0.05	55.73 ± 0.04
**2**	10:1	0.50 : 0.05	71.20 ± 0.04
**3**	12:1	0.60 : 0.05	82.90 ± 0.08
**4**	14:1	0.70 : 0.05	98.5 ± 0.1
**5**	16:1	0.80 : 0.05	116.3 ± 0.1

2.4.15 Kinetics of fluorination of cholestenone enol acetate 19 by Selectfluor™ 7

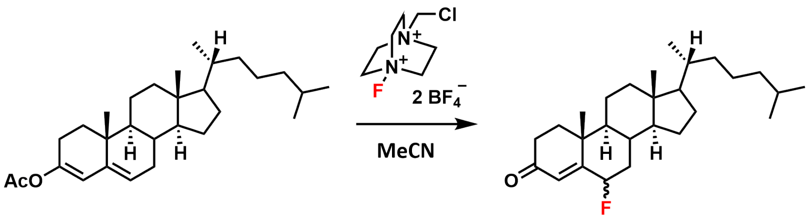

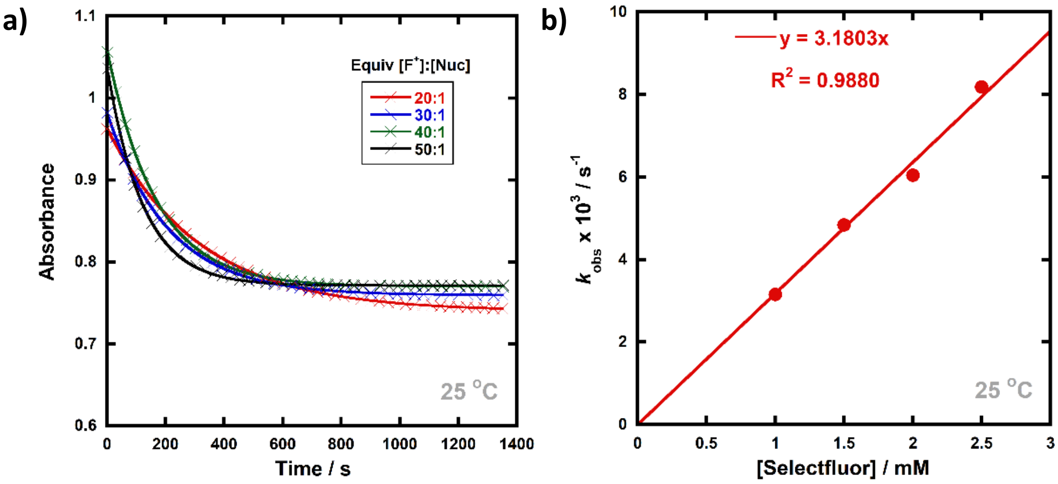

Figure 53: (a) Exponential decays of absorbance of cholestenone enol acetate **19** with different concentrations of Selectfluor™ **7** in MeCN at 25 °C. (b) Correlation of k_{obs} with [Selectfluor™].

Table 34: k_{obs} values at different concentrations of Selectfluor™ **7** in MeCN at 25 °C. Errors are standard error values.

Experiment	Ratio of Elec : Nuc	[Elec] : [Nuc] / mM	$k_{obs} \times 10^3$ / s^{-1}
**1**	20:1	1.00 : 0.05	3.154 ± 0.008
**2**	30:1	1.50 : 0.05	4.83 ± 0.02
**3**	40:1	2.00 : 0.05	6.04 ± 0.01
**4**	50:1	2.50 : 0.05	8.18 ± 0.07

2.4.16 Kinetics of fluorination of cholestenone enol acetate 19 by pentaCl-NFPy TfO⁻ 12

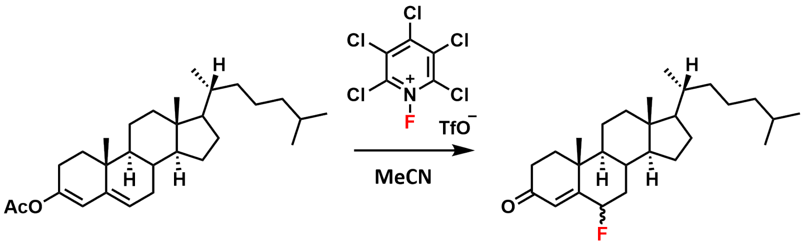

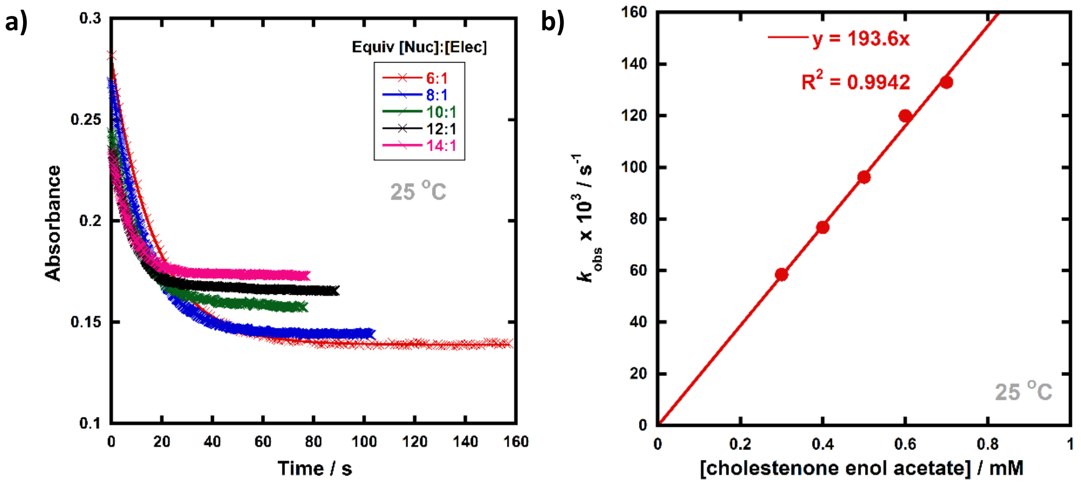

Figure 54: (a) Exponential decays of absorbance of pentaCl-NFPy TfO⁻ **12** with different concentrations of cholestenone enol acetate **19** in MeCN at 25 °C. (b) Correlation of k_{obs} with [cholestenone enol acetate **19**].

Table 35: k_{obs} values at different concentrations of cholestenone enol acetate **19** in MeCN at 25 °C. Errors are standard error values.

Experiment	Ratio of Nuc : Elec	[Nuc] : [Elec] / mM	$k_{obs} \times 10^3$ / s^{-1}
**1**	6:1	0.30 : 0.05	58.4 ± 0.2
**2**	8:1	0.40 : 0.05	76.75 ± 0.07
**3**	10:1	0.50 : 0.05	96.2 ± 0.3
**4**	12:1	0.60 : 0.05	119.9 ± 0.3
**5**	14:1	0.70 : 0.05	133.0 ± 0.3

2.4.17 Kinetics of fluorination of hydrocortisone enol tetraacetate 20 by Selectfluor™ 7

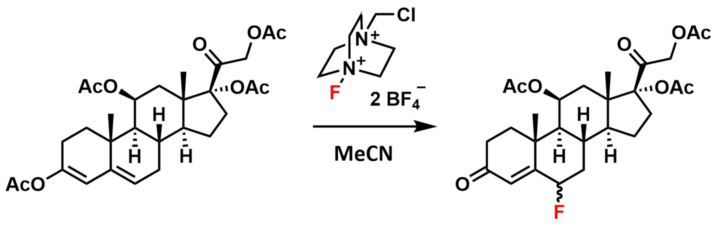

20 **24** α/β

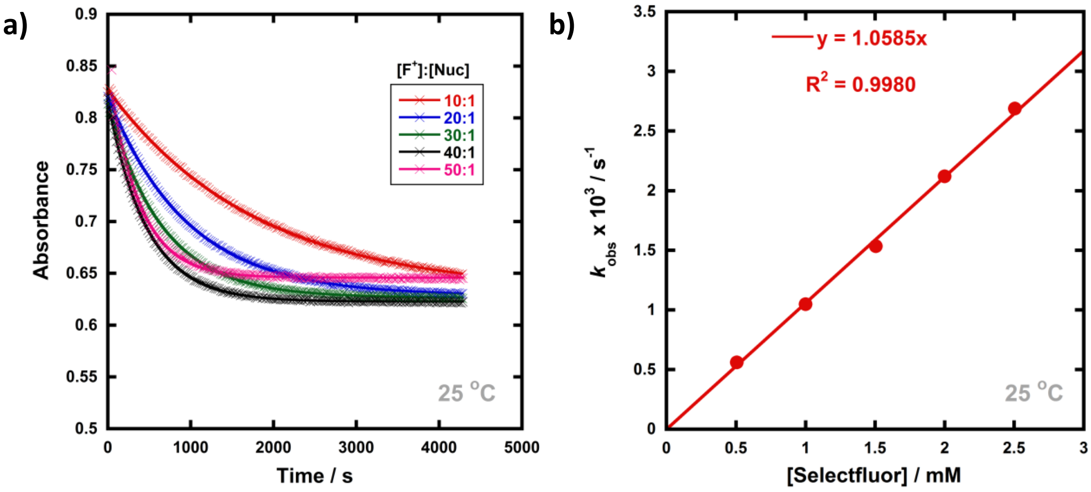

Figure 55: (a) Exponential decays of absorbance of hydrocortisone enol tetraacetate **20** with different concentrations of Selectfluor™ in MeCN at 25 °C. (b) Correlation of k_{obs} with [Selectfluor™].

Table 36: k_{obs} values at different concentrations of Selectfluor™ **7** in MeCN at 25 °C. Errors are standard error values.

**Experiment**	**Ratio of Elec : Nuc**	**[Elec] : [Nuc] / mM**	$k_{obs} \times 10^3$ / s^{-1}
**1**	10:1	0.51 : 0.05	0.559 ± 0.002
**2**	20:1	1.00 : 0.05	1.048 ± 0.002
**3**	30:1	1.51 : 0.05	1.533 ± 0.004
**4**	40:1	2.00 : 0.05	2.120 ± 0.005
**5**	50:1	2.51 : 0.05	2.688 ± 0.002

2.4.18 Kinetics of fluorination of hydrocortisone enol tetraacetate 20 by pentaCl-NFPy TfO$^-$ 12

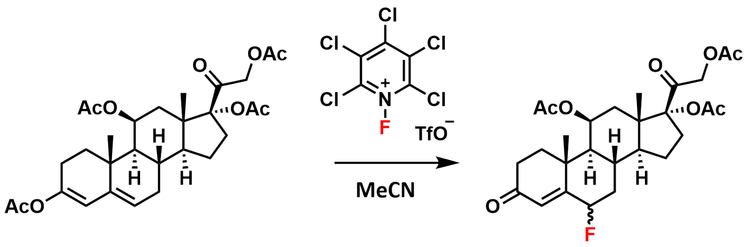

20 24 α/β

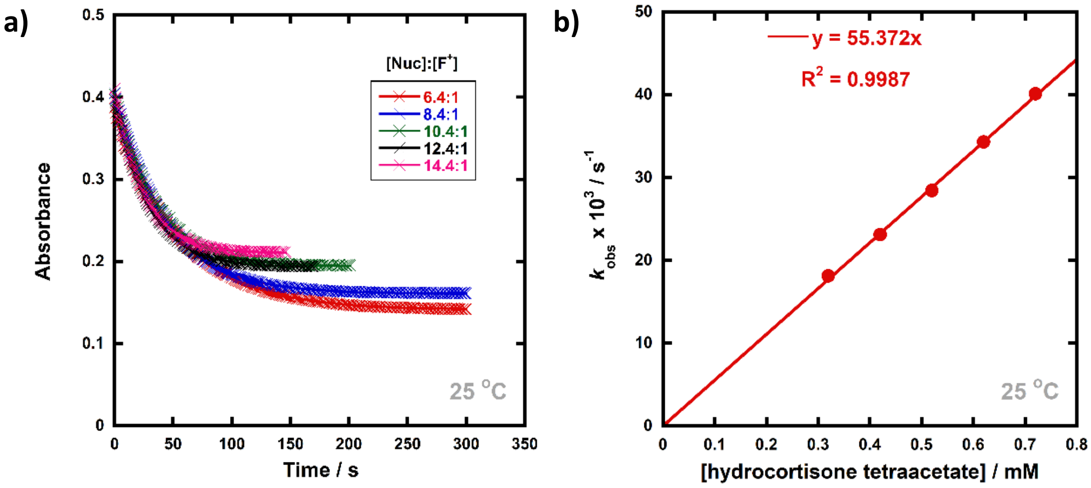

Figure 56: (a) Exponential decays of absorbance of pentaCl-NFPy TfO$^-$ **12** with different concentrations of hydrocortisone enol tetraacetate **20** in MeCN at 25 °C. (b) Correlation of k_{obs} with [hydrocortisone enol tetraacetate **20**].

Table 37: k_{obs} values at different concentrations of hydrocortisone enol tetraacetate **20** in MeCN at 25 °C. Errors are standard error values.

Experiment	Ratio of Nuc : Elec	[Nuc] : [Elec] / mM	$k_{obs} \times 10^3$ / s^{-1}
**1**	6.4:1	0.32 : 0.05	18.11 ± 0.05
**2**	8.4:1	0.42 : 0.05	23.09 ± 0.03
**3**	10.4:1	0.52 : 0.05	28.41 ± 0.07
**4**	12.4:1	0.62 : 0.05	34.29 ± 0.06
**5**	14.4:1	0.72 : 0.05	40.11 ± 0.08

2.5 Comparison of electrophilicities and nucleophilicities

The k_{rel} values of **17** and **26b** for each N−F reagent are in excellent agreement, with differences of only 0.6 to 1.4-fold, reinforcing the predictive nature of our reactivity scale.

Table 38: Summary of rate constants (k_2) for the fluorination of progesterone enol acetate **17** by N−F reagents in MeCN or MeCN-d_3 at 25 °C, and k_{rel} values determined using Equation 2 from the main text. The k_{rel} values for 1,3-dicarbonyl **26b** obtained in our previous publication[1] are included for comparison.

Electrophile	k_2 (**17**) / M^{-1} s^{-1}	k_{rel} (**17**)	k_{rel} (**26b**)	k_{rel} (**17**) / k_{rel} (**26b**)
Selectfluor™ **7**	2.38	1.0	1.0	1.0
NFSI **8**	3.33×10^{-4}	1.4×10^{-4}	2.2×10^{-4}	0.6
NFPy TfO^- **9**	2.08×10^{-5}	8.7×10^{-6}	1.1×10^{-5}	0.8
triMe-NFPy TfO^- **10**	7.19×10^{-6}	3.0×10^{-6}	2.1×10^{-6}	1.4
diCl-NFPy TfO^- **11a**	4.72×10^{-1}	2.0×10^{-1}	2.5×10^{-1}	0.8
diCl-NFPy BF_4^- **11b**	5.03×10^{-1}	2.1×10^{-1}	1.8×10^{-1}	1.2
pentaCl-NFPy TfO^- **12**	1.31×10^{2}	5.5×10^{1}	4.2×10^{1}	1.3

Table 39: The k_{rel}'' values obtained using Equation 4 (from the main text) and second-order rate constants (k_2, obtained in MeCN at 25 °C) which were used to determine k_{rel}''.

Nucleophile	k_2(Selectfluor™) / M^{-1} s^{-1}	k_2(NFSI) / M^{-1} s^{-1}	k_{rel}'' (Selectfluor™)	k_{rel}'' (NFSI)
**26a**, R = H	4.20×10^{-2}	9.87×10^{-6}	1.0	1.0
**26b**, R = OMe	6.43×10^{-1}	1.38×10^{-4}	1.5×10^{1}	1.4×10^{1}
**26c**, R = Cl	1.82×10^{-2}	5.75×10^{-6}	4.3×10^{-1}	5.8×10^{-1}
**26d**, R = CN	1.60×10^{-3}	-	3.8×10^{-2}	-
**26e**, R = NO_2	8.99×10^{-4}	-	2.1×10^{-2}	-
**26f**, R = NMe_2	1.05×10^{2}	1.41×10^{-2}	2.5×10^{3}	1.4×10^{3}
**26g**, R = F	3.28×10^{-2}	8.14×10^{-6}	7.8×10^{-1}	8.2×10^{-1}
**26h**, R = Me	1.17×10^{-1}	3.08×10^{-5}	2.8	3.1
**17**	2.38	3.33×10^{-4}	5.7×10^{1}	3.4×10^{1}

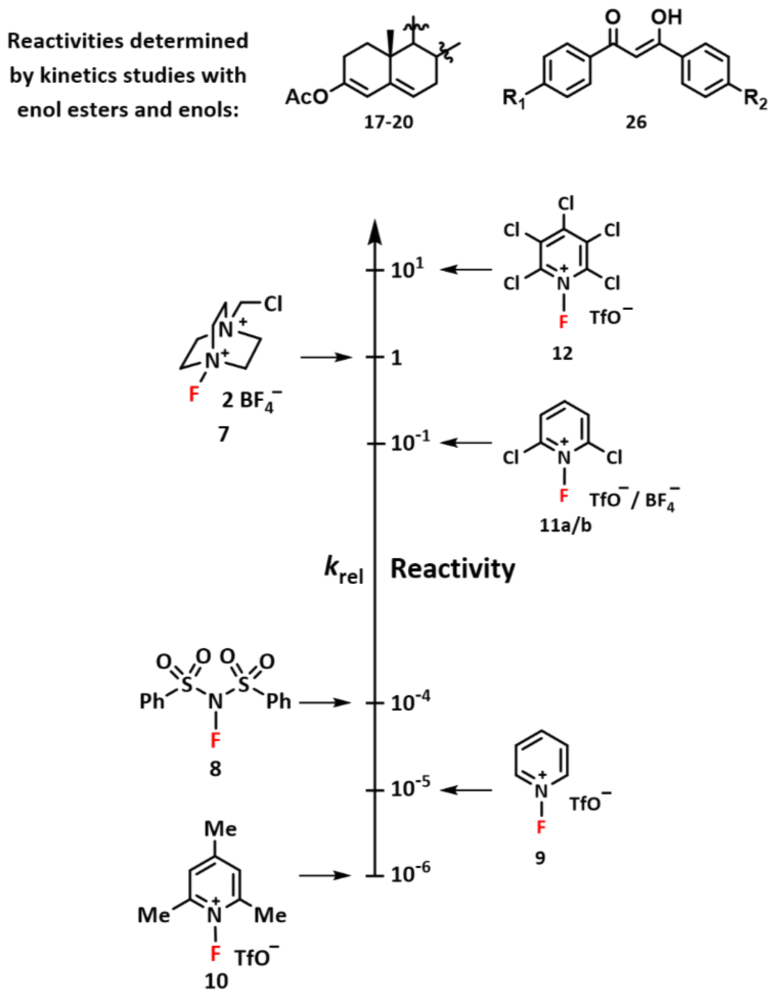

Figure 57: Reactivity scale for N−F reagents **7-12** determined using rate constants, k_2, for fluorination of steroidal enol esters and enolic 1,3-dicarbonyl systems.

2.6 Kinetics studies on epimerisation of β-fluoroprogesterone to α-fluoroprogesterone

In these experiments, hydrogen chloride solution, 1.0 M in acetic acid (Sigma Aldrich-Merck) was used and diluted to the required concentration with acetic acid. Reactions were conducted by adding **21-β** (60 mM) to a solution of HCl in acetic acid (0.25-1.0 M) in NMR tubes equipped with D_2O lock tubes, allowing the reactions to be monitored directly by ^{19}F NMR spectroscopy. Relaxation delays of 10 s were used to achieve quantitative integrals and spectra were acquired every 15 min for several hours until end-points were reached. To confirm that the experiment was quantitative, the integrals at each timepoint were summed and were found to vary by only ~5% throughout the reaction, where this variation is also likely to be due to the drift in NMR shims at later timepoints, as well as the intrinsic signal-to-noise ratios of the integrated signals.

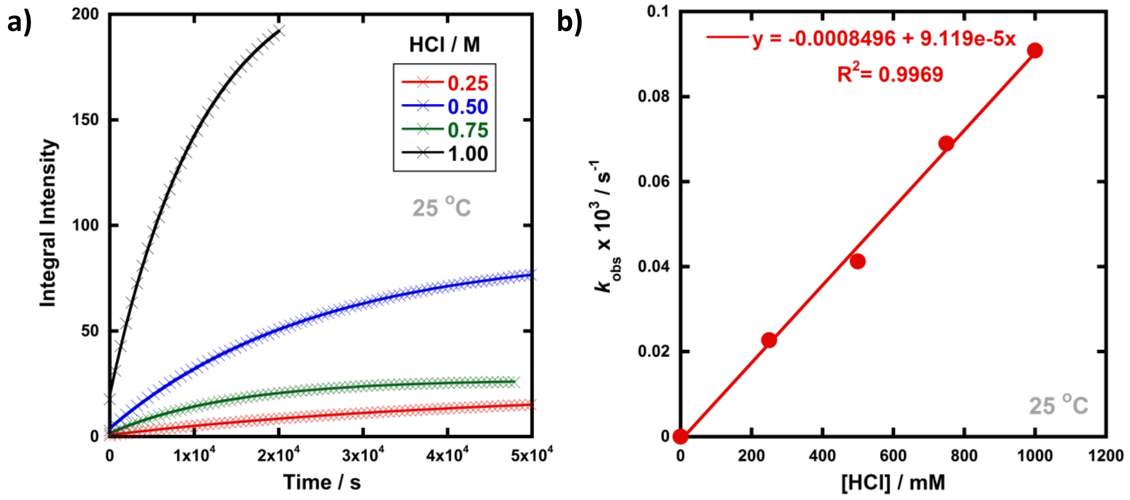

Figure 58: (a) Epimerisation of **21-β** to **21-α** in a solution of HCl in AcOH, with different concentrations of HCl, showing integrals of **21-α** at δ = −183.03 ppm over time. (d) Correlation of k_{obs} values versus [HCl].

All k_{obs} values are summarised in **Table 40**, in addition to those obtained for the rate of formation of the smaller signal at δ = −183.20 ppm. The ratios of the larger peak at −183.03 ppm to the smaller one at −183.20 ppm at the end-points of all reactions was approximately 5:1.

Table 40: The k_{obs} values for epimerisation of **21-β** to **21-α** with different concentrations of HCl in AcOH.

[HCl] / M	k_{obs} (−183.03) × 10^5 / s^{-1}	k_{obs} (−183.20) × 10^5 / s^{-1}
0.25	2.27	-[a]
0.50	4.12	3.75
0.75	6.90	-[a]
1.00	9.08	8.08

[a] Due to overlapping signals and drift in NMR shims as reactions progressed, it was not possible to determine accurate k_{obs} values.

An authentic sample of **21-α** was incubated in solutions of AcOH, 0.50 M HCl in AcOH, and 1.00 M HCl in AcOH for 45 min and ^{19}F NMR spectra were acquired. In the proton-coupled spectra for HCl-AcOH solutions, the smaller peaks at δ = −183.10 and −183.20 ppm adjacent to the major peaks at δ = −183.03 and −183.15 ppm were present (**Figure 59a**), and this was also observed in proton-decoupled spectra (**Figure 59b**). However, signals corresponding to only one species were observed in AcOH with no added HCl. This confirms that the additional peaks are indeed either due to protonation of the fluorosteroid isomers, or the formation of hemiacetals.

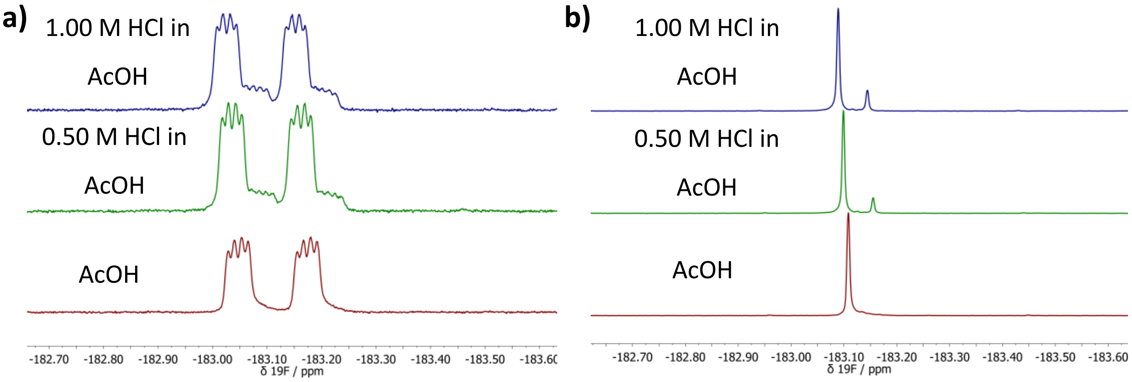

Figure 59: ^{19}F NMR spectra for **21-α** in AcOH, 0.50 M HCl in AcOH and 1.00 M HCl in AcOH, where (a) are proton-coupled; (b) are proton-decoupled.

Figure 60a shows the proton-decoupled ^{19}F NMR spectrum of the epimerisation mixture (with 0.50 M HCl in AcOH) at the end-point of the reaction. Following work-up of the epimerisation mixtures, the crude products were obtained as yellow oils, where NMR analyses showed that the α and β fluoro-isomers were present in 4:1 ratios (**Figure 60b**). The LC-MS chromatogram

trace for the crude products of the reaction performed in 0.50 M HCl in AcOH is shown in **Figure 61**. The largest peak at R_t = 2.66 min corresponds to **21-α** (as confirmed by comparison with the chromatogram of an authentic sample). The adjacent peak at R_t = 2.71 min is due to **21-β**. For the smaller peak at R_t = 2.79 min, the molecular ion signal had the same mass as the fluoroprogesterone isomers. This peak is likely to be associated with that observed in the ^{19}F NMR spectrum of the crude product after work-up at δ = −165.90 ppm (**Figure 60b**). Finally, the peak at R_t = 2.83 min corresponds to progesterone **13**; this could have formed due to loss of the fluorine atom from species **27-β** by dehydrohalogenation, forming HF, which has been previously reported in steroid systems.[10]

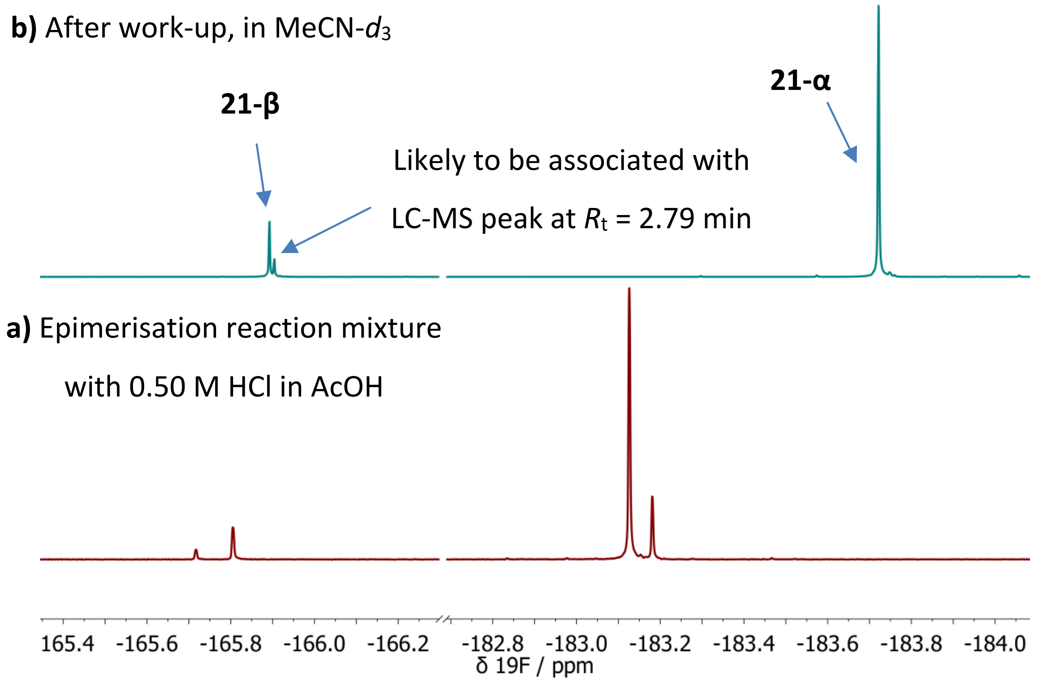

Figure 60: (a) Proton-decoupled ^{19}F NMR spectrum of the reaction mixture at the end of an epimerisation reaction (0.50 M HCl in AcOH). (b) Proton-decoupled ^{19}F NMR spectrum of crude product after work-up, in MeCN-d_3.

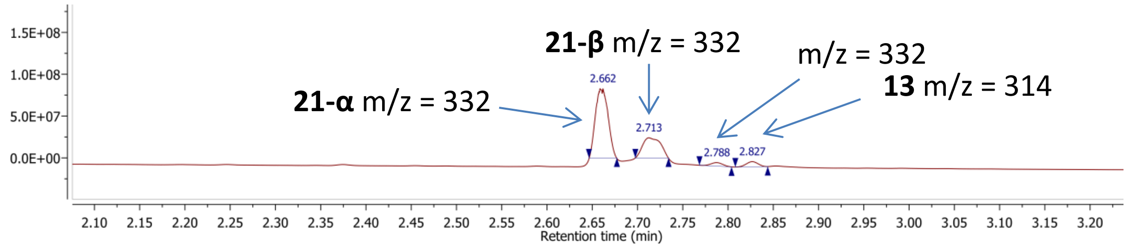

Figure 61: LC-MS chromatogram trace for crude product obtained upon work-up of epimerisation reactions.

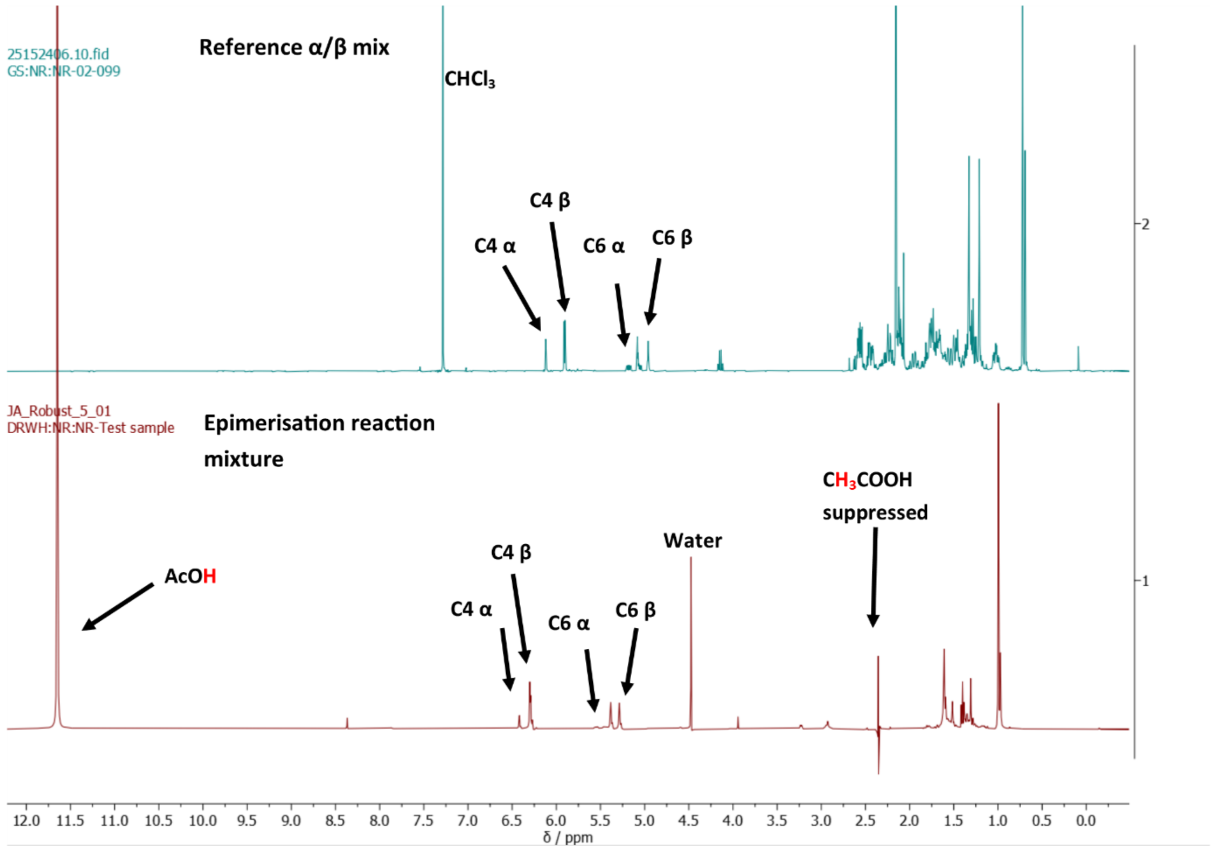

Figure 62: Spectrum 1: ^{1}H NMR spectrum of epimerisation reaction (1.0 M HCl in AcOH) at initial stages of the reaction, with solvent suppression applied to methyl peak of AcOH. Steroid peaks adjacent to the suppressed solvent peak were also suppressed. **Spectrum 2:** ^{1}H NMR spectrum of authentic sample of **21-α/21-β** in $CDCl_3$.

3. References

[1] N. Rozatian, I. W. Ashworth, G. Sandford, D. R. W. Hodgson, *Chem. Sci.* **2018**, *9*, 8692–8702.
[2] N. Rozatian, A. Beeby, I. W. Ashworth, G. Sandford, D. R. W. Hodgson, *Chem. Sci.* **2019**, *10*, 10318–10330.
[3] P. N. Rao, L. R. Axelrod, *J. Org. Chem.* **1961**, *26*, 1607–1608.
[4] A. Bowers, H. J. Ringold, *Tetrahedron* **1958**, *3*, 14–27.
[5] A. Gioiello, R. Sardella, E. Rosatelli, B. M. Sadeghpour, B. Natalini, R. Pellicciari, *Steroids* **2012**, *77*, 250–254.
[6] A. Bowers, E. Denot, R. Becerra, *J. Am. Chem. Soc.* **1960**, *82*, 4007–4012.
[7] A. J. Liston, P. Toft, *J. Org. Chem.* **1968**, *33*, 3109–3113.
[8] R. H. Cox, E. Y. Spencer, *Can. J. Chem.* **1951**, *29*, 398–408.
[9] P. Marwah, A. Marwah, H. A. Lardy, *Tetrahedron* **2003**, *59*, 2273–2287.
[10] V. Reydellet-Casey, D. J. Knoechel, P. M. Herrinton, *Org. Process Res. Dev.* **1997**, *1*, 217–221.
